# An integrative approach to medical laboratory equipment risk management

**DOI:** 10.1038/s41598-024-54334-z

**Published:** 2024-02-19

**Authors:** Neven Saleh, Omnia Gamal, Mohamed A. A. Eldosoky, Abdel Rahman Shaaban

**Affiliations:** 1 Electrical Communication and Electronic Systems Engineering Department, Faculty of Engineering, October University for Modern Sciences and Arts (MSA), 6th of October City, Giza, Egypt; 2 Systems and Biomedical Engineering Department, Higher Institute of Engineering, Shorouk Academy, Al Shorouk City, Cairo, Egypt; 3 Biomedical Engineering Department, Faculty of Engineering, Helwan University, Cairo, Egypt

**Keywords:** Risk management, FMEA, TOPSIS, Machine learning, Medical laboratory, Multi-criteria decision-making, Health care, Risk factors, Engineering

## Abstract

Medical Laboratory Equipment (MLE) is one of the most influential means for diagnosing a patient in healthcare facilities. The accuracy and dependability of clinical laboratory testing is essential for making disease diagnosis. A risk-reduction plan for managing MLE is presented in the study. The methodology was initially based on the Failure Mode and Effects Analysis (FMEA) method. Because of the drawbacks of standard FMEA implementation, a Technique for Ordering Preference by Similarity to the Ideal Solution (TOPSIS) was adopted in addition to the Simple Additive Weighting (SAW) method. Each piece of MLE under investigation was given a risk priority number (RPN), which in turn assigned its risk level. The equipment performance can be improved, and maintenance work can be prioritized using the generated RPN values. Moreover, five machine learning classifiers were employed to classify TOPSIS results for appropriate decision-making. The current study was conducted on 15 various hospitals in Egypt, utilizing a 150 MLE set of data from an actual laboratory, considering three different types of MLE. By applying the TOPSIS and SAW methods, new RPN values were obtained to rank the MLE risk. Because of its stability in ranking the MLE risk value compared to the conventional FMEA and SAW methods, the TOPSIS approach has been accepted. Thus, a prioritized list of MLEs was identified to make decisions related to appropriate incoming maintenance and scrapping strategies according to the guidance of machine learning classifiers.

## Introduction

Risk management has become an essential concept in hospitals to guarantee standard compliance, competency, reliability, and patient safety^1^. Techniques for risk management have an essential role in enhancing work environment. The risk management approach’s initial step is to identify potential hazards. Next, each hazard’s likelihood and degree of severity are evaluated. A risk number is computed to evaluate the risk connected to each hazard; hazards are then ranked to be reduced according to this number. This study aims to manage and mitigate risks including medical laboratory equipment (MLE) because of inadequate use and poor management.

A common technique for evaluating risk is the FMEA which stands for Failure Mode and Effect Analysis. By figuring out the likelihood, impact, and detectability of a failure, risk assessment is carried out using such a method. Utilizing a risk priority number (RPN), potential MLE failure scenarios are assessed. Three indicators on a range of 1 to 5 are multiplied to create the RPN, which is an aggregate index: probability (P), severity (S), and detection (D). Based on the failure’s likelihood, consequence level, and degree of detectability, an integrated risk score is proposed to compare failures^1–3^.

Multi-criteria decision-making (MCDM) is the approach to choosing the most appropriate option. In literature, several MCDM methods have been developed. One form of the MCDM is the Technique for Order Preference by Similarity to the Ideal Solution (TOPSIS) technique^4^. Mainly, it is used to select a superior alternative according to a set of conflict criteria. There are several domains in which the TOPSIS conduct has been used, such as, energy^5^, medicine^6,7^, industrial engineering systems^8^, environmental and safety issues^9,10^, research on water resources^11–13^, and various applications in chemical engineering^11,12^. Simple Additive Weighting (SAW) is one of the earliest methods of MCDM that has proven its consistency in various applications^4^.

The study suggests using both the TOPSIS and the SAW methods to compensate for the drawbacks of the conventional FMEA, which may result in different RPN values depending on the values of P, S, and D. This could be explained as an improper assessment of failures among different experts. Furthermore, the FMEA does not take the decision-making into account. Additionally, to classify the output of utilized MCDM methods, an application of machine learning (ML) was conducted. Therefore, the study’s contributions are shown to be (i) getting beyond the limitations of conventional FMEA through applying the TOPSIS and SAW for MLE risk sorting; (ii) collecting actual set of data that belongs to 3 types of MLE; (iii) proposing new factors for risk assessment; (iv) applying five ML classifiers to classify the MLE according to its real status; and (v) presenting a framework to assist biomedical engineers make decisions on MLE risk management.

This is the structure of the remaining part of the article. A Literature review of the study includes a review of the conducted literature. All explanations and descriptions of the methodology were covered in Materials and Methods. The findings of conducting the study are presented in Results, and the discussion of the results is presented in Discussion. Beside outlining the study, Conclusions offer recommendations for additional work.

## Literature review

In the healthcare sector, more concentration has been placed on risk management to guarantee regulatory compliance, efficacy, stability, and safety. Several strategies are offered for medical purposes to minimize the risks associated with medical devices. The study is an improved version of the work presented in^1^. The authors discuss more details and explanations of the proposed approach. Besides, additional techniques are presented to control the risk of each MLE under investigation.

An ordered weighted aggregation operator was suggested by Parand et al.^2^ to investigate potential hazards related to medical equipment. To compute the RPN, an innovative application was used in the model’s development. In another study, Sally et al.^14^ introduced the dynamic risk and how it could be automatically controlled for different modalities of radiology devices. The study proposed that poor management was the main cause of this kind of risk. The study reduced the risks related to device mismanagement by utilizing cloud applications and FMEA techniques. Behnam Vahdani et al.^15^ conducted a new presentation for the FMEA. It combines the TOPSIS technique and fuzzy belief structure into one paradigm to enhance the risk assessment process.

In developing nations, a dynamic system-based concept for cutting down on medical laboratory turnaround times was introduced by Abeer et al.^16^. Another study introduced the FMEA throughout the life cycle of medical device to assess associated risks. Highlighting key elements of different processes was the aim of the study, and then by calculating the RPN, potential casualties and losses can be avoided^17^. In^18^, for applied FMEA, the Deng entropy weighted risk priority number (DEWRPN) technique was first presented. The model gives a new DEWRPN in relation to the Dempster-Shafer Evidence Theory (DST).

## Materials and methods

**Figure 1:**
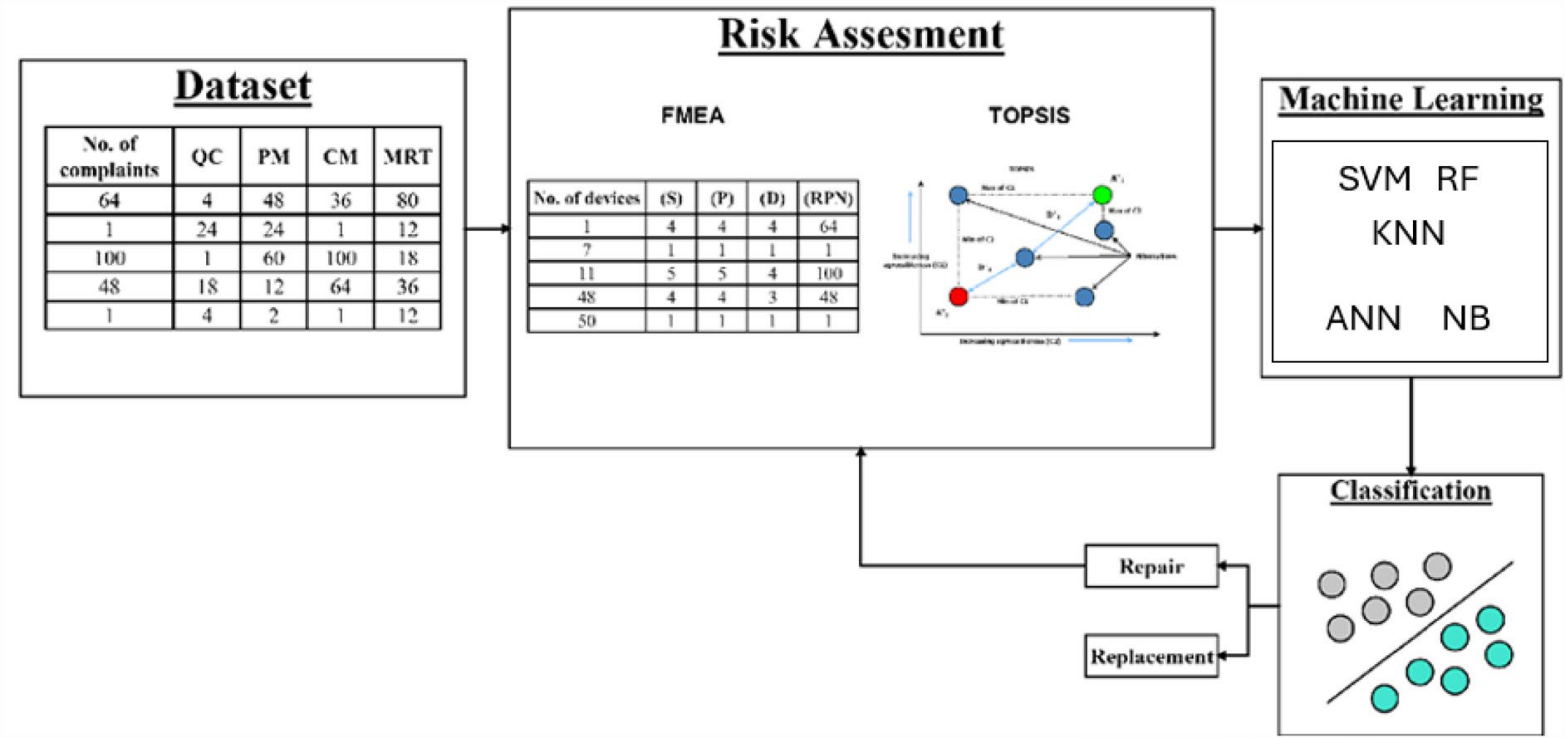
Overall methodology adopted for risk management model applied to medical laboratory equipment.

The study was conducted by applying three methods for risk assessment of MLE: the FMEA, TOPSIS, and SAW. By applying the FMEA, every piece of MLE was ranked according to the determined RPN. Besides, the risk score, which ranked all MLEs under assessment, was computed using the TOPSIS and SAW methods. Considering the results of SAW and TOPSIS, the method that presents the most robust results will be adopted for the next stage. Moreover, five ML classifiers have been employed to classify the output of the best MCDM method based on a specific threshold. The overall methodology is depicted in Fig. 1. The criteria that are proposed by the study are covered and described in the following sub-sections:

### Criteria identification

A series of criteria need to be defined for measuring and evaluating the hazards connected to any process to identify the risk. By implementing this rule, we put out five criteria for figuring out the MLE’s risk level. The annual count of complaints, the annual count of preventive maintenance (PM), the annual count of corrective maintenance (CM), the hourly mean response time (MRT) for visits, and the annual count of quality control (QC) are all noteworthy criteria^1^. As far as we are aware, in contrast to earlier studies, the quantity of QC is a new criterion.

### Methods implementation

In this study, a risk-reduction method for managing MLE is presented. The FMEA was used to calculate each MLE’s RPN. Also, the MCDM approach is used because of the shortcomings of the conventional FMEA. The TOPSIS has been selected for this purpose. It is widely employed in the healthcare domain^19–21^. Finding the opposing criteria and their respective weights is essential to ranking each alternative in the MCDM technique. There are numerous techniques for determining criteria weights, comprising both the CRITIC (Criterion Importance via Inter Criteria Correlation) entropy methods^22^. The entropy method was employed since it is appropriate for the underlined problem. This fitness is due to its ability to measure the degree of uncertainty for each criterion separately, which thereby indicates the differentiation between all criteria^21^.

#### FMEA method

The FMEA can be viewed as a bottom–up analysis method that is used to identify potential failures in a system or a service^23^. In another words, it is an inductive method that includes drawing general conclusions by reasoning through circumstances. The FMEA was employed to identify failures of MLE with high risks to seek proper management action. In its implementation, a questionnaire was conducted through five medical experts to assign steps of the FMEA as follows^1^:

- Determine each MLE failure mode and its associated impact.
- Evaluate each MLE failure mode’s relative risk as well as the consequences (severity).
- For every failure, every likely cause is identified.
- Assess the relative risk of each MLE failure (Occurrence)
- Describe the existed process controls to determine the failure mode.
- Assess the relative risk of all process controls (detection)
- Calculate the values of RPN as described in Eq. (1) and prioritize the list.
  1 $${\text{RPN}} = {\text{P}} \times {\text{S}} \times {\text{D}}$$

As demonstrated in Tables 1, 2, and 3, a range from 1 to 5 on Likert scale is used to score the probability, severity, and detectability indications. The most likely, the worst, but the least noticeable is shown in number five. Conversely, number one offers the least probable, lowest severe, and most noticeable^1^.

**Table 1:** Conventional FMEA likelihood grade ^1^.

Scale	Failure probability	Probability margins
1	Unusual	Probability < 2
2	Minimal	2 ≤ Probability > 3
3	Somewhat	3 ≤ Probability > 4
4	Elevated	4 ≤ Probability > 5
5	Extremely elevated	Probability ≥ 5

**Table 2:** Conventional FMEA severity grade^1^.

Scale	Impact degree	Consequence
1	Minor	No impact
2	Somewhat	There is little impact on the system functioning, thus maintenance might not be necessary
3	Serious	The system’s performance is significantly impacted, necessitating maintenance
4	Major	The system may continue to operate, but its performance will be declined
5	Catastrophic	High failure mode severity, accompanied by a warning, indicates a potentially hazardous outcome

**Table 3:** Conventional FMEA detectability scale^1^.

Scale	Detection degree	Likelihood of detection
1	Almost certain	Failure mode detection and subsequent failure mode will occur
2	High	A high/mechanism’s likelihood of identifying the impending failure mode and its eventual failure mode
3	Moderate	A moderate/mechanism is the subsequent failure mode, and it is possible to identify its possible occurrence
4	Remote	It is possible to identify a failure mode that could occur remotely, via a mechanism, and to identify the failure mode that follows
5	Very remote	Determining the mechanism responsible for the failure mode and its subsequent failure mode is highly improbable

#### Entropy method

The entropy method is used for criteria weighting. It is based on measuring information discrimination among criteria. A high divergence index reflects high criterion dispersion^22^. Based on the next procedures, the entropy method is used^1,4,24^.

*Step 1* The function of normalized decision matrix *N* is given in Eq. (2), where *x* presents alternative *i* against criterion *j.*

2 $${\text{N}} = \left[ {Nij} \right] = \frac{xij}{{\sqrt {\mathop \sum \nolimits_{i = 1}^{m} {\text{xij}}} }},\quad {\text{i}} = {1},{2}, \ldots {\text{m}},\;{\text{j}} = {1},{2}, \ldots ,{\text{n}}$$

*Step 2* Utilizing the Eq. (3) to compute the entropy measure *(E)* for every normalized value, where *m* represents the number of alternatives.

3 $$\begin{aligned} Ej & = - {\text{c}} \mathop \sum \limits_{i = 1}^{m} Nij\;{\text{ln}}\;Nij,\quad {\text{i}} = {1},{2}, \ldots ,{\text{m}} \\ c & = 1/ln\left( m \right) \\ \end{aligned}$$

*Step 3* Computing the relative weight taking into consideration the entropy measure *(E)* as in Eq. (4), where *n* represents the number of criteria.

4 $$Wj = \frac{1 - Ej}{{\sqrt {\mathop \sum \nolimits_{j = 1}^{n} \left( {1 - {\text{Ej}}} \right)} }},\quad {\text{j}} = {1},{2}, \ldots ,{\text{n}}$$

#### TOPSIS method

Of all the MCDM strategies, the most utilized is the distance-based Technique for Order Preference by Similarity to the Ideal Solution (TOPSIS)^25^. Finding the best option should be determined by how far it is from both the superior positive and negative solutions. The following listed procedures are used to implement the TOPSIS technique^1,4,26^.

*Step 1* Define the decision matrix *X* = [*x*_*ij*_] in which each element *x* presents alternative *i* against criterion *j*. Give definitions for both non-beneficial and beneficial factors. Maximum values are the best values under beneficial criteria, whereas lowest numbers are the best numbers under non-beneficial criteria.

*Step 2* Normalized decision matrix is calculated through the Eq. (5), where each element *x*_*ij*_ is mapped.

5 $$R = \left[ {r_{ij} } \right] = \frac{xij}{{\sqrt {\mathop \sum \nolimits_{i = 1}^{n} xij^{2} } }}$$

*Step 3* Determine the decision matrix’s weighted normalized state *S* = [v_ij_] where each element *r*_*ij*_ is assigned a weight based on the computed weight (W) as indicated by Eq. (6).

6 $$S = \left[ {{\text{v}}_{{{\text{ij}}}} } \right] = {\text{rij}} \times W_{{\text{j}}}$$

*Step 4* Calculate the optimal worst and the optimal best values. Assume that S^−^ represents the least desirable option, while S^+^ indicates the most preferred option. Equations (7) and (8) indicate both cases. The V^-^ value presents the minimum value of each weighted normalized element for beneficial criteria and the maximum value of each weighted normalized element for non-beneficial criteria. In contrast, the V^+^ presents the maximum value of each weighted normalized element for the beneficial criteria and the minimum value of each weighted normalized element for the non-beneficial criteria.

7 $$S^{ - } = \left\{ {{\text{V}}^{ - }_{{1}} , \ldots ,{\text{V}}^{*}_{{\text{m}}} } \right\} = \{ ({\text{min}}\;{\text{vij}}|{\text{j}} \in {\text{J}}),({\text{maxi}}\;{\text{vij}}|{\text{j}} \in {\text{J}}\_)\}$$

8 $$S^{ + } = \left\{ {{\text{V}}^{ + }_{{1}} , \ldots ,{\text{V}}^{*}_{{\text{m}}} } \right\} = \{ ({\text{max}}\;{\text{vij}}|{\text{j}} \in {\text{J}}),({\text{mini}}\;{\text{vij}}|{\text{j}} \in {\text{J}}\_)\}$$

*Step 5* Compute the Euclidean distance between the optimal worst and optimal best solutions. The process of deviating from a positive superior solution is referred to as “ideal differentiation,” in accordance with Eq. (9). Conversely, Eq. (10) can be used to calculate “negative ideal differentiation,” which is the process of diverging from a negative ideal solution.

9 $$D_{i}^{ + } = \sqrt {\mathop \sum \limits_{j = 1}^{m} \left( {v_{ij} - v_{j}^{*} } \right)^{2} ,} \quad i = 1, \ldots ,n$$

10 $$D_{i}^{ - } = \sqrt {\mathop \sum \limits_{j = 1}^{m} \left( {v_{ij} - v_{j}^{ - } } \right)^{2} } ,\quad i = 1, \ldots ,n$$

*Step 6* Eq. (11) is used to calculate the relative proximity between each alternative and the best solution for alternative ranking. The option with an RC value that is closest to 1 is the best choice because the RC value falls between 0 and 1.

11 $$RC_{i} = \frac{{D_{i}^{ - } }}{{D_{i}^{*} + D_{i}^{ - } }},\quad i = 1, \ldots ,n$$

Regarding the TOPSIS procedures implementation, the new values of the probability, severity, and detectability are determined, therefore, the new RPN values are calculated. Noting that, RC range is between 0 and 1. The best alternative is with the largest value of RC. In case of TOPSIS method records the best results, and to decide about scrapping or not, the RC for each device is mapped on a new scale called the transformed score value (TSV)^27^, as described by Eq. (12). The term “min” points to the minimum value of the RC, and “max” indicates the maximum value of the RC.

12 $${\text{TSV}} = \left( {{\text{RC}} - {\text{min}}} \right)/\left( {{\text{max}} - {\text{min}}} \right) \times {1}00$$

The selected threshold is taken into consideration when making decisions after applying TSV to TOPSIS results. If the equipment’s evaluated score is at least this amount, it must be repaired or maintained; if not, it should be discarded. Five consultant engineers with an average experience of 15 ± 4.1 years were selected as the number of specialists to serve as a reference guide when choosing the threshold range. As a result, 70% is the ideal cutoff point.

#### SAW method

The definition of Simple Additive Weighting (SAW) is a value function that is established by multiplying the weights by the simple addition of scores that indicate objective achievement under each criterion^4^. Variations in criterion can be offset by it. The SAW method is the traditional, simple, and widely used multi-criteria assessment method that it is also known as the weighing direct merging method. Implementation of this method entails simple two steps as shown below^4,21^.

*Step 1* Calculate the normalized ***r***_***ij***_ value as shown in Eq. (13) for both beneficial and non-beneficial criteria. The parameters x_ij_, ***i****, ****j*** are identical to those explained in the TOPSIS method.

13 $$\left. {\begin{array}{*{20}l} {r_{ij} = \frac{{x_{ij} }}{{{\text{max}}(x_{ij} )}}\left( {{\text{beneficial}}\;{\text{criteria}}} \right)} \hfill \\ { r_{ij} = \frac{{{\text{min}}(x_{ij} )}}{{x_{ij} }}\left( {{\text{non - beneficial}}\;{\text{criteria}}} \right)} \hfill \\ \end{array} } \right\}$$

*Step 2* Assign a preference index (L) for each alternative by calculating total summation of each normalized value multiplied by each criterion weight as presented in Eq. (14). The parameters ***w, r****, ****i****, ****j****, ****and m*** are identical to those explained in the TOPSIS method.

14 $$L_{i} = \mathop \sum \limits_{j = 1}^{m} w_{j} r_{ij}$$

According to a computed preference index for each alternative, all alternatives are ranked. The highest preference index is given to the best alternative, and vice versa for the lowest preference index. If the SAW method yields the best results, Eq. (12) will be applied to SAW output and RC will be replaced with L value.

#### Machine learning classifiers

Machine learning techniques, either supervised or non-supervised, are widely utilized to classify different forms of data. Based on the input data, ML has produced several patterns that can be recognized to make wise decisions. For predicting outcomes, the ML is an especially effective technique. Creating a model that incorporates relationships that result in the most potent out-of-sample predictions involves recognizing patterns or relationships in a sample of data. To find the most potent predictors, the model is run on data subsamples several times, and then it is tested on other data subsamples^28^. In application, many classifiers are employed for various classification tasks; disease diagnosis is an example^28,29^. One advantage of using supervised ML algorithms in classification is their ability to conduct over non-parametric data regardless of the type of relationship among variables^30^. The Support Vector Machine (SVM), Decision Tree (DT), Naïve Bayes (NB), Random Forest (RF), K-Nearest Neighbor (K-NN), and Artificial Neural Network (ANN) are the most common classifiers that fall into this category^30^. The K-NN is a simple and quick algorithm, in addition to its superiority for multimodal classes. The NB algorithm is characterized by its ease implementation for predicting discrete and continuous data. Besides, it requires less data for training and is not influenced by non-contributing features. The SVM can achieve reliable performance with small-scale data. A decision tree is characterized by its special behavior in finding local optimal solutions rather than global optimal solutions. To overcome this shortage, the random forest algorithm is revealed. The ANN mimics the human brain in data processing and analysis. Interactions among neurons control the input–output relationship^31^. As far as the authors know, the application of ML algorithms to classify the risk level for MLE is rarely conducted. In this way, no comparative studies present specific ML classifiers for comparison. The study therefore suggested five distinct ML classifiers employing K-NN, SVM, NB, RF, and ANN to classify the output of the best MCDM model based on the applicability of these classifiers.

Typically used in supervised machine learning, SVM is a classifier powered by creating an appropriate hyperplane for separating distinguished classes^29^. The classification principle of the K-NN is that related objects tend to be near one another^29^. Random forest is an ensemble model that uses decision trees in which a set of data is divided to minimize variability. Each tree selects a random training sample, and then a subset of variables is randomly chosen per tree. Finally, the individual trees are combined to form what is called a random forest for voting^30^. A simple probabilistic classifier known as NB depends on the probability of inputs and whether these inputs are independent^32^. Despite its simplicity, it is widely applied in various areas. The ANN is composed of many highly connected units called neurons, which are connected in specific arrangement to solve a problem. The network’s architecture comprises three layers: input, hidden, and output. A connection weight, which is assigned based on the neurons’ training, controls all connections^31^.

To evaluate the performance of SVM, K-NN, RF, NB, and ANN an array of evaluation metrics has been applied, as presented in Eqs. (15)–(18). The metrics incorporate, *accuracy*, *recall*, *precision*, and *F1-score*^33,34^. Remember that TN stands for true negative, FP for false positive, TP for true positive, and FN abbreviates false negative.

15 $${\text{Accuracy}} = \left( {{\text{TP}} + {\text{TN}}} \right)/\left( {{\text{TP}} + {\text{FN}} + {\text{FP}} + {\text{TN}}} \right)$$

16 $${\text{Precision}} = {\text{TP}}/\left( {{\text{TP}} + {\text{FP}}} \right)$$

17 $${\text{Recall}}\left( {{\text{sensitivity}}} \right) = {\text{TP}}/\left( {{\text{TP}} + {\text{FN}}} \right)$$

18 $${\text{F1 - score}} = \frac{{{\text{Precision}}\left( {{\text{avg}}} \right) \times {\text{Recall}}\left( {{\text{avg}}} \right)}}{{{\text{Precision}}\left( {{\text{avg}}} \right) + {\text{Recall}}\left( {{\text{avg}}} \right)}}$$

## Result

A set of MLEs was selected to test the methodology that integrates the FMEA the TOPSIS, and the SAW. Datasets belonging to 150 MLEs were gathered from true laboratory data from 15 various Egyptian hospitals. It was divided equally among 50 centrifuge devices, 50 hematology analyzers, and 50 chemistry analyzers. The datasets were collected between January 2020 and December 2020, spanning a full year. Considering the small size of data, it was due to a lack of documentation for some hospitals, and not all targeted hospitals have responded to data requirement.

### FMEA results

Five professionals from five public hospitals in Egypt developed the FMEA model. The P, S, and D parameters are first assigned on a scale ranging from 1 to 5 throughout the questionnaire. Subsequently, the average of each parameter for each of the five criteria yields the P, S, and D scores. A data sample comprising four chemistry analyzers is presented as an example for FMEA implementation. Table 4 shows the P, S, and D grades according to experts’ ratings. On the other side, the RPN, average values of complaints (C1), QC (C2), PM (C3), CM (C4), and MRT hours (C5) are determined individually. Table 5 demonstrates only the number of preventive maintenance as a sample. The other four parameters are calculated exactly as in Table 5. The highest value of the RPN among the five parameters is considered the equipment RPN score. Table 6 illustrates the RPN calculations for the sample under investigation.

**Table 4:** The chemistry analyzers sample demonstrated the values P, S, and D according to five experts’ ratings.

		Experts severity					Experts probability					Experts detection				
Device No.	Criterion	#1	#2	#3	#4	#5	#1	#2	#3	#4	#5	#1	#2	#3	#4	#5
1	C1	5	4	5	4	5	5	4	5	4	5	2	1	3	2	2
7		4	5	3	4	4	3	2	4	3	3	2	1	3	2	2
25		3	2	4	3	3	3	2	4	3	3	2	2	1	2	3
50		5	4	5	4	5	5	4	5	4	5	3	3	3	2	4
1	C2	5	5	4	4	5	5	5	4	4	5	1	1	1	1	2
7		2	2	1	2	3	3	3	3	2	4	2	2	1	2	3
25		4	5	3	4	4	4	5	3	4	4	1	1	1	1	2
50		5	4	5	4	5	5	4	5	4	5	2	1	3	2	2
1	C3	4	5	3	4	4	4	5	3	4	4	2	2	1	2	3
7		4	5	3	4	4	4	5	3	4	4	2	2	1	2	3
25		3	3	3	2	4	3	3	3	2	4	2	2	1	2	3
50		4	5	3	4	4	4	5	3	4	4	2	1	3	2	2
1	C4	5	4	5	4	5	4	3	4	4	5	2	2	1	2	3
7		3	3	3	2	4	3	3	3	2	4	3	2	4	3	3
25		5	4	5	4	5	5	4	5	4	5	2	1	3	2	2
50		4	5	3	4	4	4	5	3	4	4	2	2	1	2	3
1	C5	3	3	3	2	4	3	3	3	2	4	2	2	1	2	3
7		5	4	5	4	5	5	4	5	4	5	1	1	1	1	2
25		4	3	4	4	5	4	3	4	4	5	2	1	3	2	2
50		5	4	5	4	5	5	4	5	4	5	1	1	1	1	2

**Table 5:** Computed RPN considering number of PM (C3) criterion.

Device No.	(S)	(P)	(D)	(RPN)
1	4	4	2	32
7	4	4	2	32
25	3	3	2	18
50	3	3	2	18

**Table 6:** The computed RPN values for the data sample of the chemistry analyzer for the five criteria illustrating the highest RPN and rank.

Device No.	C1	C2	C3	C4	C5	Highest RPN	Rank
1	50	25	32	40	18	50	9
7	24	12	32	36	50	50	9
25	18	16	18	12	32	32	34
50	75	50	18	50	50	75	1

### TOPSIS results

Using the TOPSIS technique to address the duplicate RPN issue that the FMEA resolves. Firstly, usage of the entropy method yielded the weights of the employed criteria as described in “Entropy method” section. Obviously, the criteria are categorized as non-beneficial and beneficial. Beneficial criteria are QC counting and PM counting, with resultant weights of 0.143939791 and 0.224379498, respectively. On the other hand, non-beneficial criteria are CM counting, complaint counting, and MRT in hours, with weights of 0.268065177, 0.248527193, and 0.115088341, respectively. As a result of applying the TOPSIS stages as described in “TOPSIS method” section, the new P, S, D, and RPN are computed. Table 7 presents the resultant RND based on the displayed P, S, and D related to the chemistry analyzer sample.

**Table 7:** Results of new RPN by applying TOPSIS method on a sample of chemistry analyzers.

Device No.	S (new)	P (new)	D (new)	RPN (new)	Rank (new)
1	0.738980	0.707362	0.449630	0.235034	14
7	0.701298	0.493844	0.507733	0.179189	23
25	0.389198	0.495668	0.449630	0.08674	40
50	0.736124	0.723508	0.637227	0.339382	2

### SAW results

The SAW method was used to conquer the problem of repeatable RPNs due to the usage of the FMEA method. Like the TOPSIS method, new RPNs were calculated for each MLE. Using the entropy weighting methods, all alternatives were ranked based on the calculated RPN. Table 8 illustrates results of the new RPN according to the application of the SAW method to the chemistry analyzer.

**Table 8:** Results of new RPN by applying SAW method on a sample of chemistry analyzer.

Device No.	S (new)	P (new)	D (new)	RPN (new)	Rank (new)
1	0.409316167	0.425046395	0.732772994	0.07728763	48
7	0.350437339	0.431868863	0.696739575	0.10544664	37
25	0.484879454	0.432445362	0.732772994	0.153650678	14
50	0.370512198	0.370512198	0.746246614	0.102444204	38

As noted in Table 8, the results of the SAW method were less convenient than the results of the TOPSIS method. For this reason, only the results of the TOPSIS method were approved to continue for the next stage, in which ML classifiers should be applied to classify the risk level of MLEs.

### Machine learning classifiers results

Machine learning classifiers have been applied to the TOPSIS output to classify each MLE into three classes: stable, maintenance, and scrapping. Two scenarios were conducted for this purpose, using the output of the TOPSIS directly and using the TSV values. Due to the small size of the data, a fivefold cross-validation test was carried out for both scenarios. For the first scenario, the five classifiers were used on the 150 MLE’s TOPSIS output without being mapped to the TSV values as shown in Table 9. It is worth mentioning that the TOPSIS method’s results have been labeled for training purposes by two experts from various hospitals. Additionally, feature vectors that are given to all classifiers comprise the five criteria (C1–C5), the RPN value for the first scenario, and TSV value for the second scenario.

**Table 9:** Results of the evaluation performance metrics for ML classifiers for the first scenario.

	SVM (%)	K-NN (%)	NB (%)	RF (%)	ANN (%)
Accuracy	92.00	87.56	90.22	92.44	91.11
Sensitivity	82.21	76.26	84.38	84.66	83.78
Precision	87.06	78.24	83.81	87.79	84.15
F1-Score	84.36	77.19	84.08	86.05	83.93

For the second scenario, all ML classifiers have been applied to devices whose TSV is less than or equal to 70%. Each classifier classified the device into two classes: replacement and scrapping. WEKA is a cutting-edge platform for developing and applying machine learning algorithms. It provides visual tools for data processing and visualization. Also, it is open-source software licensed under a public license. This study has used WEKA program version 3.8.6 for implementation. The performance metrics results for the second scenario are introduced in Table 10. According to the proposed algorithms, the K number for K-NN was 3, for SVM, a linear kernel function was used, and 6 trees were selected for training RF. As shown in Tables 9 and 10, the RF algorithm yields the best classification results for both scenarios.

**Table 10:** Results of the evaluation performance metrics for ML classifiers for the second scenario.

	SVM (%)	K-NN (%)	NB (%)	RF (%)	ANN (%)
Accuracy	97.67	94.57	96.12	99.22	96.90
Sensitivity	97.71	94.52	96.32	99.18	96.89
Precision	96.92	95.29	92.54	100.00	96.89
F1-Score	97.31	94.90	94.36	99.59	96.89

**Figure 2:**
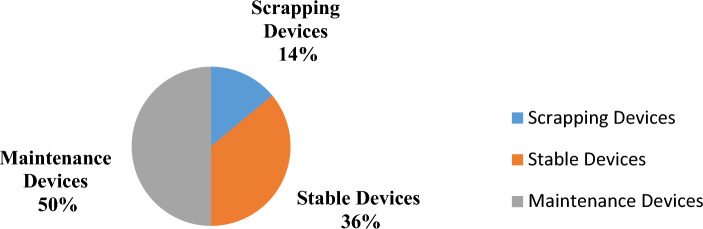
Classification results of total MLE after applying TOPSIS and RF models.

According to the results of classification, each device under investigation should be placed in one of three categories: stable device, replacement required, and scrapping or risk reassessment. According to the dataset that belongs to 150 MLE, results of RF for the second scenario revealed that 54 devices (36%) would be stable, 75 devices (50%) would be serviceable, and 21 devices (14%) would be scrapped (those with TSV ≥ 70%), as shown in Fig. 2. In summary, Table 11 provides an overview of the raw data of an MLE sample along with the categorization status that was obtained.

**Table 11:** A sample of investigated MLE with its raw data of the five criteria and resultant TSV values with their categories.

Device	C1	C2	C3	C4	C5	TSV%	Devices status
Hematology analyzer	1	6	6	1	48	9.8649	Stable
Chemistry analyzer	8	6	2	6	84	65.7752	Maintenance
Centrifuge	12	10	2	12	60	100	Scrapping

## Discussion

The study presented three methods for evaluating the risk number for MLE, and this number is used to determine the priority of maintenance and risk reduction. In this application, three methods were used: the FMEA, the TOPSIS, and the SAW. The shortcomings in typical FMEA were alleviated using TOPSIS and SAW by avoiding repeatable RPNs. However, the results of TOPSIS have demonstrated convenient performance compared to the SAW method, leading to the adoption of TOPSIS. Consequently, the TOPSIS technique gives each MLE a distinct RPN, leading to greater consistency in outcomes. By applying the FMEA, it is noted that there are similarities in RPN value. For hematology analyzers, for instance, devices 11 and 15 are ranked first and have the same RPN. Devices 5 and 7 in the chemistry analyzer category are ranked ninth and share the same RPN. In terms of centrifuge analyzers, devices 9 and 27 are placed 34th and each has an identical RPN. Even though the number of complaints is different for all of them.

To prevent this issue, the TOPSIS technique was used. For example, for hematology analyzers, device 11 is ranked first, while device 15 is ranked at 16. For chemistry analyzers, devices 5 and 7 are ranked at 15 and 23, respectively. Similarly, centrifuge analyzers, devices 9 and 27, have different values of the RPN, and consequently different rankings being 3 for device 9 and 41 for device 27. Besides that, applying ML classifiers is a robust solution to categorize the MLE into discard, and scrapping/risk reassessment. For this, two scenarios were run: one using the TSV values and the other directly using the TOPSIS result. According to the results, using the TSV values yielded robust performance. This solution has been applied to only risky MLEs based on the calculated TSV percentage. As a result, three categories of MLE are presented in the study. The RF classifier produced the most convenient results for classification, as shown in Table 10. This can be interpreted as reliability of RF for training small databases. Also, RF is appropriate for handling a mixture of categorical and numerical features^34^. Sometimes it works outside the box (Supplementary information).

According to the results of the paradigm, 36% of the devices are stable, and 50% of them require service or risk reassessment. For model validation, two professionals with twelve years of combined experience were asked to assess a total of 86% of the devices as either stable or serviceable. As a result, depending on the given criteria for investigated devices, they classified the devices into 68 stable devices (46%), and 61 serviceable devices (40%). Comparing the paradigm’s results with the experts’ result, we notice how accurately the paradigm classifies the MLEs. Furthermore, the paradigm enlarges the margin of serviceable MLEs by 10%, which leads to improving the safety of selected MLEs.

Since the problem underlined is rarely focused on literature, few articles could be considered for benchmarking. We found that our study’s objective is like that of the *Sally *et al*.*^14^ study when we compared it to prior studies, but it differs in methodology and medical equipment type. Although our study is relevant to Vahdani et al.^15^ in methodology, the fuzzy TOPSIS was employed instead of standard TOPSIS. Furthermore, the application was steel production, not medical devices.

## Conclusions

The study concerns risk assessment in medical laboratory equipment management, using two distinct methodologies. Both the MCDM approach in the forms of the TOPSIS and the SAW and the risk analysis tool, the FMEA, are used. The drawbacks of the FMEA are resolved by TOPSIS and SAW; however, TOPSIS was the best. A unique RPN is generated for each piece of equipment to identify the risk priority. The ranking of the MLE list is rational with respect to equipment raw data. It guides the proper incoming action for the decision-maker. For example, maintenance and scrapping phases are investigated for further improved strategies. For this reason, machine learning classifiers were applied to distinguish risky devices as either reusable or disposable. The RF classifier was approved as robust for solving this problem. Also, the proposed criteria have reflected their significance in identifying the risk priority of the MLE. Some criteria are highlighted because they affect risk levels, such as complaint registration and quality control recording. To this point, quality control work must be done constantly for the devices, and the response to complaints must be speedy within 48 h. Moreover, the PM should be frequently carried out at least four times a year. This study’s future work will encompass the use of Evaluation based on Distance from Average Solution (EDAS) as an alternative MCDM paradigm. Furthermore, additional criteria, such as mean time between failures, downtime, and failure rate, could be examined. Besides, more devices from different types of MLE should be placed under investigation. In this way, other machine learning classifiers would be tested.

### Supplementary Information

Supplementary Information.

## Data Availability

All data generated or analyzed during this study are included in this published article [and its supplementary information files].
