# Supplementary material for: An integrative approach to medical laboratory equipment risk management
Source: Sci Rep. 2024 Feb 19;14:4045. doi: 10.1038/s41598-024-54334-z (PMC10876531; doi:10.1038/s41598-024-54334-z)
Supplement: Supplementary file 1 — Supplementary Information. [file 41598_2024_54334_MOESM1_ESM.docx]

**Manuscript Title:** An Integrative Approach to Medical Laboratory Equipment Risk Management

Raw data for underlined medical laboratory equipment

The haematology analyzer’s data

| Device no. | SN | Type | Model | No.of complaints per Year | PM (preventive maintenance) per Year | CM (Corrective maintenance) per Year | Response time for visit per Hour | Quality control (Qc) per Year |
| --- | --- | --- | --- | --- | --- | --- | --- | --- |
| 1 | B3220 | Sysmex | KX-21 | 8 | 2 | 6 | 84 | 6 |
| 2 | B7292 | Sysmex | KX-21N | 6 | 3 | 4 | 48 | 3 |
| 3 | P2559 | Sysmex | XP-100 | 4 | 3 | 4 | 72 | 4 |
| 4 | P2575 | Sysmex | XP-300 | 4 | 6 | 3 | 36 | 6 |
| 5 | A2040 | Sysmex | CA-540 | 5 | 3 | 4 | 72 | 3 |
| 6 | 15865 | Sysmex | XS-500i | 10 | 2 | 8 | 96 | 4 |
| 7 | B4700 | Sysmex | KX-21 | 2 | 3 | 2 | 36 | 3 |
| 8 | RM-32103720 | Mindary | BC-3200 | 9 | 2 | 9 | 24 | 6 |
| 9 | RP-16106963 | Mindary | BC-2800 | 6 | 2 | 5 | 36 | 3 |
| 10 | RJ-39115793 | Mindary | BC-3000Plus | 9 | 2 | 8 | 36 | 8 |
| 11 | RJ-36115208 | Mindary | BC-3000Plus | 12 | 2 | 12 | 60 | 10 |
| 12 | RQ-91101239 | Mindary | BC-2600 | 10 | 2 | 9 | 84 | 6 |
| 13 | RJ-18109420 | Mindary | BC-3000Plus | 6 | 2 | 4 | 84 | 4 |
| 14 | RP-95102666 | Mindary | BC-2800 | 9 | 2 | 9 | 84 | 6 |
| 15 | H33o01E1285 | Hemax | Hemax330 | 7 | 1 | 6 | 96 | 4 |
| 16 | 12462 | NIHON KOHDEN | MEK-6410k | 5 | 3 | 4 | 48 | 4 |
| 17 | 4802 | NIHON KOHDEN | MEK-6510k | 4 | 4 | 4 | 48 | 4 |
| 18 | 4574 | NIHON KOHDEN | MEK-6510k | 3 | 4 | 3 | 48 | 4 |
| 19 | 101295 | Swelab | Alfa piuse | 9 | 1 | 9 | 48 | 9 |
| 20 | A2728 | Sysmex | CA-540 | 3 | 6 | 2 | 48 | 6 |
| 21 | RP-94102633 | Mindary | BC-2800 | 4 | 3 | 3 | 96 | 3 |
| 22 | 4782 | NIHON KOHDENCelltac Alpha | MEK-6510k | 3 | 4 | 3 | 72 | 4 |
| 23 | RJ-45117803 | Mindary | BC-3000Plus | 4 | 2 | 4 | 72 | 4 |
| 24 | 11419 | Sysmex | XS-500i | 6 | 2 | 5 | 72 | 5 |
| 25 | RP-45113544 | Mindary | BC-2800 | 3 | 3 | 3 | 84 | 3 |
| 26 | 4067 | NIHON KOHDENCelltac Alpha | MEK-6510k | 4 | 6 | 4 | 60 | 6 |
| 27 | RM-87100489 | Mindary | BC-3200 | 10 | 2 | 10 | 36 | 8 |
| 28 | 13345 | NIHON KOHDENCelltac Alpha | MEK-6410k | 3 | 4 | 3 | 72 | 4 |
| 29 | RP-01104127 | Mindary | BC-2800 | 8 | 1 | 8 | 72 | 8 |
| 30 | RM-24103330 | Mindary | BC-3200 | 6 | 2 | 4 | 60 | 4 |
| 31 | RJ-36115207 | Mindary | BC-3000Plus | 5 | 3 | 5 | 84 | 3 |
| 32 | 1150 | NIHON KOHDENCelltac Alpha | MEK-6510k | 4 | 2 | 4 | 72 | 4 |
| 33 | 1677 | NIHON KOHDENCelltac Alpha | MEK-7300k | 3 | 4 | 3 | 84 | 4 |
| 34 | 4795 | NIHON KOHDENCelltac Alpha | MEK-6510k | 2 | 4 | 2 | 84 | 4 |
| 35 | 3920 | NIHON KOHDENCelltac Alpha | MEK-6318k | 5 | 2 | 4 | 36 | 4 |
| 36 | 4805 | NIHON KOHDENCelltac Alpha | MEK-6510k | 3 | 2 | 3 | 36 | 3 |
| 37 | RM-8510022 | Mindary | BC-3200 | 6 | 2 | 5 | 24 | 5 |
| 38 | 30654 | ERMA Inc | PCE-210N | 10 | 2 | 8 | 72 | 8 |
| 39 | 606-OT-80690 | HORIBA ABX | Micros 60 | 6 | 1 | 4 | 72 | 4 |
| 40 | 101291 | Swelab | Alfa | 12 | 1 | 12 | 72 | 12 |
| 41 | 24559 | ERMA Inc. | PCE-210N | 6 | 1 | 4 | 72 | 4 |
| 42 | 15800 | Sysmex | XS-500i | 3 | 2 | 3 | 96 | 3 |
| 43 | 607OT81-135 | Horiba ABX | Micros 60- OT | 6 | 2 | 5 | 84 | 5 |
| 44 | 4148 | Nihon kohden | MEK-6510K | 2 | 3 | 2 | 72 | 3 |
| 45 | 15871 | Sysmex | XS-500i | 1 | 6 | 1 | 48 | 6 |
| 46 | 4801 | NIHON KOHDENCelltac Alpha | MEK-6510k | 3 | 2 | 3 | 36 | 3 |
| 47 | 1414 | NIHON KOHDENCelltac Alpha | MEK-6510k | 4 | 2 | 3 | 84 | 3 |
| 48 | 11921 | NIHON KOHDENCelltac Alpha | MEK-6410k | 8 | 4 | 8 | 72 | 4 |
| 49 | 12366 | NIHON KOHDENCelltac Alpha | MEK-6410k | 4 | 4 | 4 | 24 | 4 |
| 50 | 4785 | NIHON KOHDENCelltac Alpha | MEK-6510k | 1 | 6 | 1 | 48 | 6 |

The chemistry analyzer’s data

| Device no. | SN | Type | Model | No.of complaints per Year | PM (preventive maintenance) per Year | CM (Corrective maintenance) per Year | Response time for visit per Hour | Quality control (Qc) |
| --- | --- | --- | --- | --- | --- | --- | --- | --- |
| 1 | WN-23103421 | mindray | BS-200 | 10 | 2 | 10 | 72 | 4 |
| 2 | WN-24103561 | mindray | BS-200 | 6 | 2 | 6 | 108 | 12 |
| 3 | WS-000924 | mindray | BS-380 | 12 | 1 | 12 | 84 | 4 |
| 4 | WS-39001715 | mindray | BS-380 | 8 | 3 | 8 | 120 | 12 |
| 5 | XP-36000285 | mindray | BS-800 | 12 | 1 | 8 | 72 | 6 |
| 6 | XP-36000283 | mindray | BS-800 | 9 | 2 | 6 | 84 | 12 |
| 7 | R5634110 | Roche | Cobas E411 | 6 | 2 | 6 | 96 | 12 |
| 8 | R5633115 | Roche | Cobas C311 | 4 | 2 | 4 | 84 | 12 |
| 9 | 16-5096 | Elitech | Microlab 300 | 1 | 4 | 1 | 36 | 12 |
| 10 | WN-02101335 | mindray | BS-200 | 10 | 2 | 10 | 48 | 12 |
| 11 | 19-43043 | Elitech Group | Microlab 300 | 2 | 3 | 2 | 48 | 12 |
| 12 | WN-02101118 | mindray | BS-200 | 8 | 2 | 8 | 84 | 6 |
| 13 | 920501 | Diasys | Respons 920 | 12 | 2 | 12 | 72 | 4 |
| 14 | 1773674 | siemens | Dimension | 12 | 1 | 9 | 48 | 12 |
| 15 | 3300441 | Apel | PD-303s | 2 | 3 | 2 | 48 | 12 |
| 16 | 14-7595 | Elitech | SELECTRA PRO M | 6 | 2 | 5 | 48 | 12 |
| 17 | 18-46 513 | Elitech | Microlab 300 | 4 | 2 | 4 | 96 | 12 |
| 18 | 19-46733 | Elitech | Microlab 300 | 3 | 3 | 3 | 84 | 12 |
| 19 | WR-93000324 | mindray | BA-88A | 2 | 3 | 2 | 72 | 12 |
| 20 | 13-9727 | Elitech | Flexor EL150 | 5 | 2 | 5 | 96 | 12 |
| 21 | 12-745 8 | Elitech | Flexor EL200 | 4 | 2 | 4 | 84 | 6 |
| 22 | WK-34103149 | mindray | BS-120 | 6 | 2 | 6 | 84 | 6 |
| 23 | 11-94 88 | Elitech | Flexor EL180 | 4 | 3 | 4 | 96 | 4 |
| 24 | 19-46733 | Elitech | Microlab 300 | 3 | 2 | 3 | 72 | 12 |
| 25 | 11-74 97 | Elitech | Flexor EL200 | 4 | 3 | 4 | 84 | 6 |
| 26 | 16-52 01 | Elitech | Microlab 300 | 5 | 2 | 4 | 120 | 6 |
| 27 | WN-02101228 | mindray | BS-200 | 6 | 2 | 6 | 84 | 12 |
| 28 | WR-97000965 | mindray | BA-88A | 4 | 3 | 4 | 84 | 12 |
| 29 | 97201 | ERMA | Inc AE-600N | 8 | 1 | 8 | 84 | 12 |
| 30 | 8787 | BECKMAN | CX9 Pro | 10 | 2 | 10 | 72 | 6 |
| 31 | 920501 | Diasys | Respons 920 | 12 | 1 | 12 | 84 | 4 |
| 32 | C1817391 | Thermo Scientific | Konelab 60i | 9 | 2 | 9 | 84 | 12 |
| 33 | 8845 | BECKMAN | CX9PRO | 5 | 3 | 5 | 72 | 12 |
| 34 | 604C40308 | Horiba medical ABX | Pentra 400 ISE | 12 | 3 | 12 | 96 | 12 |
| 35 | 1810 | BASIC | Secoman | 12 | 1 | 10 | 96 | 12 |
| 36 | 13-7521 | Elitech | Flexor EL200 | 6 | 2 | 6 | 120 | 4 |
| 37 | 14-7601 | Elitech | SELECTRA PRO M | 8 | 2 | 8 | 84 | 12 |
| 38 | 14-7597 | Elitech | SELECTRA PRO M | 6 | 2 | 5 | 84 | 12 |
| 39 | 15-5079 | Elitech | Microlab 300 | 4 | 2 | 4 | 108 | 12 |
| 40 | 14-6031 | Elitech | Microlab 300 | 3 | 2 | 3 | 84 | 12 |
| 41 | 14-9635 | Elitech | Flexor EL150 | 6 | 2 | 5 | 108 | 12 |
| 42 | 11-94 89 | Elitech | Flexor EL180 | 5 | 2 | 5 | 120 | 12 |
| 43 | 14-97 59 | Elitech | SELECTRA PRO S | 6 | 3 | 6 | 84 | 12 |
| 44 | 19-46189 | Elitech | Microlab 300 | 6 | 2 | 6 | 84 | 6 |
| 45 | 13-8314 | Elitech | Flexor xl | 10 | 3 | 8 | 120 | 12 |
| 46 | WR-97000865 | mindray | BA-88A | 2 | 4 | 2 | 96 | 12 |
| 47 | 10-53 35 | Elitech | Microlab 300 | 1 | 6 | 1 | 72 | 12 |
| 48 | 13-9749 | Elitech | Flexor pro150 | 8 | 2 | 6 | 96 | 12 |
| 49 | 19-46780 | Elitech | Microlab 300 | 1 | 3 | 1 | 96 | 12 |
| 50 | 13-7560 | Elitech | Flexor EL200 | 12 | 3 | 12 | 108 | 4 |

The centrifuge data

| Device no. | SN | Type | Model | No.of complaints per Year | PM (preventive maintenance) per Year | CM (Corrective maintenance) per Year | Response time for visit in Hour |
| --- | --- | --- | --- | --- | --- | --- | --- |
| 1 | H456890 | Hitachi | TD4 | 1 | 1 | 1 | 36 |
| 2 | H456885 | Hitachi | TD4 | 1 | 1 | 1 | 72 |
| 3 | H456895 | Hitachi | TD4 | 1 | 1 | 1 | 48 |
| 4 | H456888 | Hitachi | TD4 | 1 | 1 | 1 | 84 |
| 5 | H456897 | Hitachi | TD4 | 2 | 1 | 1 | 36 |
| 6 | 3992665 | Jouan B4 | B4 | 3 | 1 | 3 | 36 |
| 7 | 73449 | B Braun BioTech | B16 | 4 | 1 | 4 | 72 |
| 8 | 42701339 | Thermo | CL4 | 2 | 1 | 2 | 96 |
| 9 | 43260 | Nuve | CN 180 | 4 | 1 | 4 | 60 |
| 10 | ALS98M25 | BECKMAN | ALLEGRA-6 | 1 | 1 | 1 | 84 |
| 11 | 52650000 | NAHITA | 2650 | 2 | 1 | 2 | 84 |
| 12 | 91205 | HORIZON | 755VES | 1 | 1 | 1 | 84 |
| 13 | 91108 | HORIZON | 755VES | 1 | 1 | 1 | 96 |
| 14 | 81025356 | Scientific System | 80 -1 | 1 | 1 | 1 | 72 |
| 15 | ALS98M18 | BECKMAN | ALLEGRA-6 | 1 | 1 | 1 | 48 |
| 16 | CJD00G03 | BECKMAN | J6-HC | 1 | 1 | 1 | 84 |
| 17 | 9000020984 | BECKMAN | 6S | 4 | 1 | 4 | 96 |
| 18 | 81025465 | Scientific System | 80 -1 | 3 | 1 | 3 | 84 |
| 19 | 42165 | Nuve | CN 180 | 2 | 1 | 2 | 72 |
| 20 | 41010651 | Nuve | NF 800 | 1 | 1 | 1 | 84 |
| 21 | 91350 | HORIZON | 755VES | 1 | 1 | 1 | 72 |
| 22 | 81025450 | Scientific System | 80 -1 | 1 | 1 | 1 | 96 |
| 23 | 16110420 | Tehtnica | Centric350 | 1 | 1 | 1 | 84 |
| 24 | 104115 | Gemmy | PLC-012E | 1 | 1 | 1 | 96 |
| 25 | 42701440 | Thermo | CL2 | 1 | 2 | 1 | 84 |
| 26 | 43465 | Nuve | CN 180 | 1 | 2 | 1 | 84 |
| 27 | 216811-22 | Centurion scientific | PRO-Hospital.8 | 1 | 1 | 1 | 72 |
| 28 | 5702AL028638 | eppendorf | 5702 | 1 | 2 | 1 | 48 |
| 29 | 41010562 | Nuve | NF 800 | 1 | 2 | 1 | 84 |
| 30 | 42701229 | Thermo | CL2 | 1 | 2 | 1 | 36 |
| 31 | 42701219 | IEC | CL2 | 1 | 2 | 1 | 48 |
| 32 | 43810 | Nuve | CN 180 | 1 | 2 | 1 | 84 |
| 33 | 1510366 | PLC Series | PLC-05 | 1 | 2 | 1 | 36 |
| 34 | 320 -0001727 | Grifoils | DGSPIN | 1 | 2 | 1 | 84 |
| 35 | 43169 | Nuve | CN 180 | 1 | 2 | 1 | 84 |
| 36 | 41153 | Nuve | CN 180 | 1 | 2 | 1 | 84 |
| 37 | 61001900 | Ortho Work Station | 6100 | 1 | 2 | 1 | 84 |
| 38 | 16110216 | Tehtnica | Centric350 | 1 | 1 | 1 | 84 |
| 39 | 16110217 | Tehtnica | Centric351 | 1 | 1 | 1 | 84 |
| 40 | 2001865 | BIORAD | 24S | 1 | 1 | 1 | 84 |
| 41 | 104105 | Gemmy | PLC-012E | 1 | 1 | 1 | 108 |
| 42 | 320-0001830 | Grifols | DG therm | 1 | 1 | 1 | 84 |
| 43 | 2001852 | BIORAD | 24S | 1 | 1 | 1 | 84 |
| 44 | 41950071 | Thermo Scientific | SL40R | 1 | 1 | 1 | 96 |
| 45 | 16110214 | Tehtnica | Centric350 | 1 | 1 | 1 | 84 |
| 46 | 3992885 | Jouan B4 | B4 | 1 | 2 | 1 | 108 |
| 47 | 41010865 | Nuve | NF 800 | 1 | 2 | 1 | 96 |
| 48 | 2001885 | BIORAD | 24S | 1 | 1 | 1 | 108 |
| 49 | CJD00G09 | BECKMAN | J6-HC | 3 | 1 | 3 | 84 |
| 50 | 9000020885 | BECKMAN | 6S | 2 | 1 | 2 | 96 |

Survey for hematology analyzer.

| First Expert Name:Eng Ahmed Hamed Mahmoud Your experience in the field of medical laboratory equipment: 20 Year Hospital: Al-Mabarrah Hospital  Second Expert Name: Eng Ibrahim Sabry Your experience in the field of medical laboratory equipment: 15 Year Hospital: Al Ahrar Hospital  Thrid Expert Name: Eng Ashraf Mohamed Your experience in the field of medical laboratory equipment: 12 Year Hospital: Abukbir Almarkazi Hospital  Fourth Expert Name: Eng Ahmed Amer Your experience in the field of medical laboratory equipment: 18 Year Hospital: Zagazig University Hospital  Fifth Expert Name: Eng Ahmed Hamdy Your experience in the field of medical laboratory equipment: 10 Year Hospital: Kafr Saqr Almarkazi Hospital | | | | | | | | | | | | | | | | | | | | | | |
| --- | --- | --- | --- | --- | --- | --- | --- | --- | --- | --- | --- | --- | --- | --- | --- | --- | --- | --- | --- | --- | --- | --- |
| Experts ratings for S, P, and D for hematology analyzers | | | | | | | | | | | | | | | | | | | | | | |
|  |  |  |  | Experts Severity | | | | |  | Experts Probability | | | | | Experts Detection | | | | |  |  |  |
| Function | Device no. | Failure mode | Effects of Failure | E1 | E2 | E3 | E4 | E5 | Average | Causes of Failure | E1 | E2 | E3 | E4 | E5 | Average | E1 | E2 | E3 | E4 | E5 | Average |
| urately diagnose the patient's condition | 1 | Some devices are not working | pressure on working devices | 4 | 3 | 4 | 5 | 4 | 4 | number of complaints | 4 | 4 | 3 | 4 | 5 | 4 | 4 | 3 | 4 | 4 | 5 | 4 |
|  | 2 |  |  | 3 | 2 | 3 | 4 | 3 | 3 |  | 3 | 2 | 3 | 3 | 3 | 3 | 3 | 2 | 3 | 4 | 3 | 3 |
|  | 3 |  |  | 3 | 2 | 3 | 3 | 3 | 3 |  | 2 | 1 | 2 | 3 | 2 | 2 | 2 | 1 | 2 | 3 | 2 | 2 |
|  | 4 |  |  | 2 | 1 | 2 | 3 | 2 | 2 |  | 2 | 1 | 3 | 2 | 2 | 2 | 2 | 1 | 3 | 2 | 2 | 2 |
|  | 5 |  |  | 3 | 3 | 4 | 4 | 3 | 3 |  | 3 | 2 | 3 | 3 | 3 | 3 | 3 | 2 | 3 | 3 | 3 | 3 |
|  | 6 |  |  | 5 | 4 | 5 | 5 | 4 | 5 |  | 4 | 3 | 4 | 5 | 4 | 4 | 3 | 2 | 4 | 3 | 3 | 3 |
|  | 7 |  |  | 1 | 1 | 2 | 1 | 2 | 1 |  | 1 | 1 | 2 | 1 | 2 | 1 | 1 | 1 | 2 | 1 | 2 | 1 |
|  | 8 |  |  | 4 | 4 | 3 | 4 | 5 | 4 |  | 4 | 4 | 3 | 4 | 5 | 4 | 4 | 3 | 4 | 4 | 5 | 4 |
|  | 9 |  |  | 3 | 2 | 3 | 3 | 3 | 3 |  | 3 | 2 | 3 | 3 | 3 | 3 | 3 | 2 | 3 | 4 | 3 | 3 |
|  | 10 |  |  | 4 | 3 | 4 | 4 | 5 | 4 |  | 4 | 3 | 4 | 5 | 4 | 4 | 4 | 3 | 4 | 4 | 5 | 4 |
|  | 11 |  |  | 5 | 4 | 5 | 5 | 4 | 5 |  | 5 | 4 | 5 | 4 | 5 | 5 | 4 | 3 | 4 | 5 | 4 | 4 |
|  | 12 |  |  | 5 | 4 | 5 | 4 | 5 | 5 |  | 4 | 4 | 3 | 4 | 5 | 4 | 3 | 2 | 3 | 4 | 3 | 3 |
|  | 13 |  |  | 3 | 2 | 3 | 4 | 3 | 3 |  | 3 | 2 | 3 | 3 | 3 | 3 | 2 | 2 | 1 | 2 | 3 | 2 |
|  | 14 |  |  | 4 | 3 | 4 | 5 | 4 | 4 |  | 4 | 3 | 4 | 5 | 4 | 4 | 3 | 2 | 3 | 4 | 3 | 3 |
|  | 15 |  |  | 5 | 5 | 4 | 4 | 5 | 5 |  | 4 | 4 | 3 | 4 | 5 | 4 | 2 | 2 | 1 | 2 | 3 | 2 |
|  | 16 |  |  | 3 | 2 | 3 | 3 | 3 | 3 |  | 3 | 2 | 3 | 3 | 3 | 3 | 3 | 2 | 3 | 4 | 3 | 3 |
|  | 17 |  |  | 2 | 1 | 2 | 2 | 3 | 2 |  | 2 | 1 | 3 | 2 | 2 | 2 | 2 | 2 | 1 | 2 | 3 | 2 |
|  | 18 |  |  | 2 | 1 | 3 | 2 | 2 | 2 |  | 2 | 1 | 2 | 3 | 2 | 2 | 1 | 1 | 2 | 1 | 2 | 1 |
|  | 19 |  |  | 4 | 3 | 4 | 5 | 4 | 4 |  | 4 | 3 | 4 | 5 | 4 | 4 | 3 | 2 | 3 | 3 | 3 | 3 |
|  | 20 |  |  | 2 | 2 | 1 | 2 | 3 | 2 |  | 2 | 1 | 2 | 2 | 3 | 2 | 3 | 2 | 4 | 3 | 3 | 3 |
|  | 21 |  |  | 3 | 2 | 3 | 4 | 3 | 3 |  | 2 | 1 | 3 | 2 | 2 | 2 | 3 | 2 | 3 | 4 | 3 | 3 |
|  | 22 |  |  | 2 | 1 | 2 | 2 | 3 | 2 |  | 2 | 2 | 1 | 2 | 3 | 2 | 3 | 2 | 3 | 3 | 3 | 3 |
|  | 23 |  |  | 2 | 1 | 3 | 2 | 2 | 2 |  | 2 | 1 | 3 | 2 | 2 | 2 | 3 | 2 | 4 | 3 | 3 | 3 |
|  | 24 |  |  | 3 | 2 | 3 | 3 | 3 | 3 |  | 3 | 2 | 3 | 4 | 3 | 3 | 3 | 2 | 3 | 4 | 3 | 3 |
|  | 25 |  |  | 2 | 2 | 1 | 2 | 3 | 2 |  | 2 | 2 | 1 | 2 | 3 | 2 | 3 | 2 | 3 | 3 | 3 | 3 |
|  | 26 |  |  | 2 | 1 | 3 | 2 | 2 | 2 |  | 2 | 1 | 3 | 2 | 2 | 2 | 3 | 2 | 4 | 3 | 3 | 3 |
|  | 27 |  |  | 5 | 4 | 5 | 4 | 5 | 5 |  | 4 | 3 | 4 | 5 | 4 | 4 | 3 | 2 | 3 | 4 | 3 | 3 |
|  | 28 |  |  | 2 | 1 | 2 | 2 | 3 | 2 |  | 2 | 1 | 2 | 2 | 3 | 2 | 3 | 2 | 3 | 3 | 3 | 3 |
|  | 29 |  |  | 3 | 2 | 3 | 4 | 3 | 3 |  | 4 | 3 | 4 | 5 | 4 | 4 | 3 | 2 | 4 | 3 | 3 | 3 |
|  | 30 |  |  | 3 | 2 | 3 | 3 | 3 | 3 |  | 3 | 2 | 3 | 3 | 3 | 3 | 3 | 2 | 3 | 4 | 3 | 3 |
|  | 31 |  |  | 3 | 2 | 3 | 3 | 2 | 3 |  | 3 | 2 | 3 | 3 | 2 | 3 | 3 | 2 | 3 | 3 | 3 | 3 |
|  | 32 |  |  | 2 | 2 | 1 | 2 | 3 | 2 |  | 2 | 2 | 1 | 2 | 3 | 2 | 2 | 2 | 1 | 2 | 3 | 2 |
|  | 33 |  |  | 2 | 1 | 2 | 2 | 3 | 2 |  | 2 | 1 | 2 | 2 | 3 | 2 | 3 | 2 | 3 | 3 | 3 | 3 |
|  | 34 |  |  | 1 | 1 | 1 | 2 | 2 | 1 |  | 1 | 1 | 1 | 2 | 2 | 1 | 3 | 2 | 4 | 3 | 3 | 3 |
|  | 35 |  |  | 3 | 2 | 3 | 3 | 3 | 3 |  | 3 | 2 | 3 | 3 | 3 | 3 | 3 | 2 | 3 | 3 | 3 | 3 |
|  | 36 |  |  | 2 | 1 | 2 | 2 | 3 | 2 |  | 2 | 1 | 2 | 2 | 3 | 2 | 3 | 2 | 4 | 3 | 3 | 3 |
|  | 37 |  |  | 3 | 2 | 3 | 4 | 3 | 3 |  | 3 | 2 | 3 | 3 | 3 | 3 | 3 | 2 | 3 | 3 | 3 | 3 |
|  | 38 |  |  | 4 | 3 | 4 | 5 | 4 | 4 |  | 4 | 3 | 4 | 5 | 4 | 4 | 3 | 2 | 4 | 3 | 3 | 3 |
|  | 39 |  |  | 3 | 2 | 3 | 3 | 4 | 3 |  | 3 | 2 | 3 | 4 | 3 | 3 | 3 | 2 | 3 | 3 | 3 | 3 |
|  | 40 |  |  | 5 | 4 | 5 | 4 | 5 | 5 |  | 5 | 4 | 5 | 4 | 5 | 5 | 3 | 2 | 4 | 3 | 3 | 3 |
|  | 41 |  |  | 3 | 2 | 3 | 3 | 4 | 3 |  | 3 | 2 | 3 | 3 | 3 | 3 | 3 | 2 | 3 | 3 | 3 | 3 |
|  | 42 |  |  | 2 | 1 | 3 | 2 | 2 | 2 |  | 2 | 1 | 2 | 2 | 3 | 2 | 3 | 2 | 4 | 3 | 3 | 3 |
|  | 43 |  |  | 3 | 2 | 3 | 3 | 3 | 3 |  | 3 | 2 | 3 | 4 | 3 | 3 | 3 | 2 | 3 | 3 | 3 | 3 |
|  | 44 |  |  | 1 | 1 | 2 | 1 | 2 | 1 |  | 1 | 1 | 2 | 1 | 2 | 1 | 3 | 2 | 4 | 3 | 3 | 3 |
|  | 45 |  |  | 1 | 2 | 1 | 1 | 2 | 1 |  | 1 | 2 | 1 | 1 | 2 | 1 | 1 | 1 | 2 | 1 | 2 | 1 |
|  | 46 |  |  | 2 | 1 | 3 | 2 | 2 | 2 |  | 2 | 2 | 1 | 2 | 3 | 2 | 3 | 2 | 3 | 3 | 3 | 3 |
|  | 47 |  |  | 2 | 1 | 2 | 3 | 2 | 2 |  | 2 | 1 | 2 | 2 | 3 | 2 | 3 | 2 | 4 | 3 | 3 | 3 |
|  | 48 |  |  | 4 | 3 | 4 | 5 | 4 | 4 |  | 4 | 3 | 4 | 5 | 4 | 4 | 3 | 2 | 3 | 3 | 3 | 3 |
|  | 49 |  |  | 2 | 1 | 3 | 2 | 2 | 2 |  | 2 | 1 | 3 | 2 | 2 | 2 | 3 | 2 | 4 | 3 | 3 | 3 |
|  | 50 |  |  | 1 | 1 | 1 | 1 | 2 | 1 |  | 1 | 1 | 1 | 1 | 2 | 1 | 1 | 1 | 2 | 1 | 2 | 1 |
|  | 1 |  | Inaccuracy of the results | 2 | 1 | 3 | 2 | 2 | 2 | Quality Control | 2 | 1 | 3 | 2 | 2 | 2 | 1 | 2 | 1 | 1 | 2 | 1 |
|  | 2 |  |  | 4 | 4 | 3 | 5 | 4 | 4 |  | 3 | 2 | 3 | 3 | 3 | 3 | 2 | 2 | 1 | 2 | 3 | 2 |
|  | 3 |  |  | 3 | 2 | 4 | 3 | 3 | 3 |  | 3 | 2 | 4 | 3 | 3 | 3 | 1 | 1 | 2 | 1 | 2 | 1 |
|  | 4 |  |  | 2 | 1 | 3 | 2 | 2 | 2 |  | 2 | 1 | 3 | 2 | 2 | 2 | 1 | 1 | 1 | 1 | 2 | 1 |
|  | 5 |  |  | 4 | 3 | 4 | 4 | 5 | 4 |  | 3 | 2 | 3 | 3 | 3 | 3 | 2 | 2 | 1 | 2 | 3 | 2 |
|  | 6 |  |  | 3 | 2 | 4 | 3 | 3 | 3 |  | 3 | 2 | 3 | 3 | 4 | 3 | 1 | 1 | 2 | 1 | 2 | 1 |
|  | 7 |  |  | 4 | 3 | 4 | 4 | 5 | 4 |  | 3 | 2 | 4 | 3 | 3 | 3 | 2 | 2 | 1 | 2 | 3 | 2 |
|  | 8 |  |  | 2 | 1 | 3 | 2 | 2 | 2 |  | 2 | 1 | 3 | 2 | 2 | 2 | 1 | 1 | 2 | 1 | 2 | 1 |
|  | 9 |  |  | 4 | 3 | 4 | 4 | 5 | 4 |  | 3 | 2 | 3 | 3 | 3 | 3 | 2 | 2 | 1 | 2 | 3 | 2 |
|  | 10 |  |  | 2 | 1 | 3 | 2 | 2 | 2 |  | 1 | 1 | 2 | 1 | 2 | 1 | 1 | 1 | 2 | 1 | 2 | 1 |
|  | 11 |  |  | 2 | 1 | 1 | 1 | 2 | 2 |  | 1 | 1 | 1 | 1 | 2 | 1 | 1 | 1 | 2 | 1 | 2 | 1 |
|  | 12 |  |  | 2 | 1 | 3 | 2 | 2 | 2 |  | 2 | 1 | 3 | 2 | 2 | 2 | 1 | 1 | 2 | 1 | 2 | 1 |
|  | 13 |  |  | 3 | 2 | 4 | 3 | 3 | 3 |  | 3 | 2 | 4 | 3 | 3 | 3 | 2 | 2 | 1 | 2 | 3 | 2 |
|  | 14 |  |  | 2 | 1 | 3 | 2 | 2 | 2 |  | 2 | 1 | 3 | 2 | 2 | 2 | 1 | 1 | 1 | 1 | 2 | 1 |
|  | 15 |  |  | 4 | 3 | 4 | 4 | 5 | 4 |  | 3 | 2 | 4 | 3 | 3 | 3 | 2 | 1 | 2 | 3 | 2 | 2 |
|  | 16 |  |  | 3 | 2 | 4 | 3 | 3 | 3 |  | 3 | 2 | 4 | 3 | 3 | 3 | 2 | 1 | 3 | 2 | 2 | 2 |
|  | 17 |  |  | 2 | 1 | 3 | 2 | 2 | 2 |  | 3 | 2 | 4 | 3 | 3 | 3 | 2 | 2 | 1 | 2 | 3 | 2 |
|  | 18 |  |  | 3 | 2 | 4 | 3 | 3 | 3 |  | 3 | 2 | 4 | 3 | 3 | 3 | 2 | 1 | 3 | 2 | 2 | 2 |
|  | 19 |  |  | 2 | 1 | 3 | 2 | 2 | 2 |  | 1 | 1 | 1 | 1 | 2 | 1 | 1 | 1 | 2 | 1 | 2 | 1 |
|  | 20 |  |  | 2 | 2 | 1 | 2 | 3 | 2 |  | 2 | 2 | 1 | 2 | 3 | 2 | 1 | 1 | 1 | 1 | 2 | 1 |
|  | 21 |  |  | 4 | 3 | 4 | 4 | 5 | 4 |  | 3 | 2 | 4 | 3 | 3 | 3 | 2 | 1 | 2 | 3 | 2 | 2 |
|  | 22 |  |  | 3 | 2 | 4 | 3 | 3 | 3 |  | 3 | 4 | 3 | 3 | 2 | 3 | 2 | 1 | 3 | 2 | 2 | 2 |
|  | 23 |  |  | 3 | 3 | 3 | 2 | 4 | 3 |  | 3 | 2 | 4 | 3 | 3 | 3 | 2 | 2 | 1 | 2 | 3 | 2 |
|  | 24 |  |  | 3 | 4 | 3 | 3 | 2 | 3 |  | 2 | 2 | 1 | 2 | 3 | 2 | 2 | 1 | 2 | 3 | 2 | 2 |
|  | 25 |  |  | 4 | 3 | 4 | 4 | 5 | 4 |  | 3 | 2 | 4 | 3 | 3 | 3 | 2 | 1 | 3 | 2 | 2 | 2 |
|  | 26 |  |  | 2 | 1 | 3 | 2 | 2 | 2 |  | 2 | 2 | 1 | 2 | 3 | 2 | 1 | 1 | 2 | 1 | 2 | 1 |
|  | 27 |  |  | 2 | 3 | 2 | 1 | 2 | 2 |  | 1 | 1 | 1 | 1 | 2 | 1 | 1 | 1 | 1 | 1 | 2 | 1 |
|  | 28 |  |  | 3 | 2 | 4 | 3 | 3 | 3 |  | 3 | 2 | 4 | 3 | 3 | 3 | 2 | 2 | 1 | 2 | 3 | 2 |
|  | 29 |  |  | 2 | 2 | 1 | 2 | 3 | 2 |  | 1 | 1 | 1 | 1 | 2 | 1 | 1 | 1 | 1 | 1 | 2 | 1 |
|  | 30 |  |  | 3 | 2 | 4 | 3 | 3 | 3 |  | 3 | 2 | 4 | 3 | 3 | 3 | 3 | 2 | 3 | 3 | 3 | 3 |
|  | 31 |  |  | 4 | 3 | 4 | 4 | 5 | 4 |  | 3 | 3 | 3 | 2 | 4 | 3 | 2 | 2 | 1 | 2 | 3 | 2 |
|  | 32 |  |  | 3 | 2 | 4 | 3 | 3 | 3 |  | 3 | 4 | 3 | 3 | 2 | 3 | 3 | 2 | 3 | 3 | 3 | 3 |
|  | 33 |  |  | 3 | 2 | 4 | 3 | 3 | 3 |  | 3 | 2 | 4 | 3 | 3 | 3 | 2 | 2 | 1 | 2 | 3 | 2 |
|  | 34 |  |  | 3 | 3 | 3 | 2 | 4 | 3 |  | 3 | 2 | 4 | 3 | 3 | 3 | 3 | 2 | 3 | 3 | 3 | 3 |
|  | 35 |  |  | 3 | 4 | 3 | 3 | 2 | 3 |  | 3 | 3 | 3 | 2 | 4 | 3 | 2 | 2 | 1 | 2 | 3 | 2 |
|  | 36 |  |  | 4 | 4 | 3 | 5 | 4 | 4 |  | 3 | 4 | 3 | 3 | 2 | 3 | 2 | 1 | 2 | 3 | 2 | 2 |
|  | 37 |  |  | 3 | 2 | 4 | 3 | 3 | 3 |  | 2 | 2 | 1 | 2 | 3 | 2 | 2 | 1 | 3 | 2 | 2 | 2 |
|  | 38 |  |  | 2 | 2 | 1 | 2 | 3 | 2 |  | 1 | 1 | 1 | 1 | 2 | 1 | 1 | 1 | 1 | 1 | 2 | 1 |
|  | 39 |  |  | 3 | 2 | 4 | 3 | 3 | 3 |  | 3 | 2 | 4 | 3 | 3 | 3 | 2 | 2 | 1 | 2 | 3 | 2 |
|  | 40 |  |  | 1 | 1 | 1 | 1 | 2 | 1 |  | 1 | 1 | 1 | 1 | 2 | 1 | 1 | 1 | 1 | 1 | 2 | 1 |
|  | 41 |  |  | 3 | 2 | 4 | 3 | 3 | 3 |  | 3 | 2 | 3 | 4 | 3 | 3 | 2 | 1 | 2 | 3 | 2 | 2 |
|  | 42 |  |  | 4 | 4 | 3 | 5 | 4 | 4 |  | 3 | 2 | 3 | 3 | 3 | 3 | 2 | 1 | 3 | 2 | 2 | 2 |
|  | 43 |  |  | 3 | 2 | 4 | 3 | 3 | 3 |  | 2 | 2 | 1 | 2 | 3 | 2 | 2 | 1 | 2 | 3 | 2 | 2 |
|  | 44 |  |  | 4 | 3 | 4 | 4 | 5 | 4 |  | 3 | 2 | 4 | 3 | 3 | 3 | 2 | 1 | 3 | 2 | 2 | 2 |
|  | 45 |  |  | 1 | 1 | 1 | 1 | 2 | 1 |  | 2 | 1 | 3 | 2 | 2 | 2 | 1 | 1 | 1 | 1 | 2 | 1 |
|  | 46 |  |  | 4 | 3 | 4 | 4 | 5 | 4 |  | 3 | 2 | 4 | 3 | 3 | 3 | 2 | 1 | 2 | 3 | 2 | 2 |
|  | 47 |  |  | 4 | 4 | 3 | 5 | 4 | 4 |  | 3 | 3 | 3 | 2 | 4 | 3 | 2 | 1 | 3 | 2 | 2 | 2 |
|  | 48 |  |  | 3 | 3 | 3 | 2 | 4 | 3 |  | 3 | 4 | 3 | 3 | 2 | 3 | 2 | 1 | 2 | 3 | 2 | 2 |
|  | 49 |  |  | 3 | 2 | 4 | 3 | 3 | 3 |  | 3 | 2 | 4 | 3 | 3 | 3 | 2 | 1 | 3 | 2 | 2 | 2 |
|  | 50 |  |  | 2 | 2 | 1 | 2 | 3 | 2 |  | 2 | 2 | 1 | 2 | 3 | 2 | 1 | 1 | 1 | 1 | 2 | 1 |
|  | 1 | Delayed test result | Patient dissatisfaction | 4 | 3 | 4 | 4 | 5 | 4 | Number Of Preventive Maintenance | 4 | 4 | 3 | 5 | 4 | 4 | 3 | 2 | 3 | 3 | 3 | 3 |
|  | 2 |  |  | 4 | 4 | 3 | 5 | 4 | 4 |  | 3 | 2 | 3 | 4 | 3 | 3 | 2 | 1 | 2 | 3 | 2 | 2 |
|  | 3 |  |  | 3 | 2 | 4 | 3 | 3 | 3 |  | 3 | 2 | 3 | 3 | 3 | 3 | 2 | 1 | 3 | 2 | 2 | 2 |
|  | 4 |  |  | 2 | 2 | 1 | 2 | 3 | 2 |  | 2 | 2 | 1 | 2 | 3 | 2 | 1 | 1 | 1 | 1 | 2 | 1 |
|  | 5 |  |  | 4 | 4 | 3 | 5 | 4 | 4 |  | 3 | 3 | 3 | 2 | 4 | 3 | 2 | 2 | 1 | 2 | 3 | 2 |
|  | 6 |  |  | 5 | 4 | 5 | 4 | 5 | 5 |  | 4 | 4 | 3 | 5 | 4 | 4 | 3 | 2 | 3 | 3 | 3 | 3 |
|  | 7 |  |  | 4 | 4 | 3 | 5 | 4 | 4 |  | 3 | 3 | 3 | 2 | 4 | 3 | 2 | 2 | 1 | 2 | 3 | 2 |
|  | 8 |  |  | 4 | 3 | 4 | 4 | 5 | 4 |  | 4 | 3 | 4 | 4 | 5 | 4 | 3 | 4 | 3 | 3 | 2 | 3 |
|  | 9 |  |  | 4 | 5 | 3 | 4 | 4 | 4 |  | 4 | 4 | 3 | 5 | 4 | 4 | 3 | 3 | 3 | 2 | 4 | 3 |
|  | 10 |  |  | 4 | 4 | 3 | 5 | 4 | 4 |  | 4 | 5 | 3 | 4 | 4 | 4 | 3 | 4 | 3 | 3 | 2 | 3 |
|  | 11 |  |  | 5 | 4 | 5 | 4 | 5 | 5 |  | 4 | 4 | 3 | 5 | 4 | 4 | 3 | 3 | 3 | 2 | 4 | 3 |
|  | 12 |  |  | 3 | 3 | 3 | 2 | 4 | 3 |  | 4 | 5 | 3 | 4 | 4 | 4 | 3 | 2 | 3 | 3 | 3 | 3 |
|  | 13 |  |  | 3 | 3 | 3 | 2 | 4 | 3 |  | 4 | 5 | 3 | 4 | 4 | 4 | 3 | 4 | 3 | 3 | 2 | 3 |
|  | 14 |  |  | 4 | 3 | 4 | 4 | 5 | 4 |  | 4 | 4 | 3 | 5 | 4 | 4 | 3 | 3 | 3 | 2 | 4 | 3 |
|  | 15 |  |  | 4 | 4 | 3 | 5 | 4 | 4 |  | 5 | 4 | 5 | 4 | 5 | 5 | 4 | 3 | 4 | 5 | 4 | 4 |
|  | 16 |  |  | 4 | 5 | 3 | 4 | 4 | 4 |  | 3 | 2 | 3 | 4 | 3 | 3 | 2 | 1 | 2 | 3 | 2 | 2 |
|  | 17 |  |  | 3 | 3 | 3 | 2 | 4 | 3 |  | 3 | 2 | 3 | 3 | 3 | 3 | 2 | 1 | 3 | 2 | 2 | 2 |
|  | 18 |  |  | 3 | 4 | 3 | 3 | 2 | 3 |  | 3 | 3 | 3 | 2 | 4 | 3 | 2 | 2 | 1 | 2 | 3 | 2 |
|  | 19 |  |  | 4 | 4 | 3 | 5 | 4 | 4 |  | 5 | 4 | 5 | 4 | 5 | 5 | 4 | 3 | 4 | 5 | 4 | 4 |
|  | 20 |  |  | 1 | 1 | 1 | 1 | 2 | 1 |  | 2 | 2 | 1 | 2 | 3 | 2 | 1 | 1 | 1 | 1 | 2 | 1 |
|  | 21 |  |  | 3 | 3 | 3 | 2 | 4 | 3 |  | 3 | 2 | 3 | 3 | 4 | 3 | 2 | 1 | 2 | 3 | 2 | 2 |
|  | 22 |  |  | 1 | 1 | 1 | 1 | 2 | 1 |  | 3 | 3 | 3 | 2 | 4 | 3 | 2 | 1 | 3 | 2 | 2 | 2 |
|  | 23 |  |  | 4 | 3 | 4 | 4 | 5 | 4 |  | 4 | 5 | 3 | 4 | 4 | 4 | 3 | 4 | 3 | 3 | 2 | 3 |
|  | 24 |  |  | 4 | 4 | 3 | 5 | 4 | 4 |  | 4 | 4 | 3 | 5 | 4 | 4 | 3 | 3 | 3 | 2 | 4 | 3 |
|  | 25 |  |  | 3 | 3 | 3 | 2 | 4 | 3 |  | 3 | 3 | 3 | 2 | 4 | 3 | 2 | 2 | 1 | 2 | 3 | 2 |
|  | 26 |  |  | 1 | 1 | 1 | 1 | 2 | 1 |  | 2 | 2 | 1 | 2 | 3 | 2 | 4 | 3 | 4 | 5 | 4 | 4 |
|  | 27 |  |  | 4 | 4 | 3 | 5 | 4 | 4 |  | 2 | 2 | 1 | 2 | 3 | 2 | 3 | 2 | 3 | 3 | 3 | 3 |
|  | 28 |  |  | 2 | 2 | 1 | 2 | 3 | 2 |  | 3 | 3 | 3 | 2 | 4 | 3 | 2 | 2 | 1 | 2 | 3 | 2 |
|  | 29 |  |  | 4 | 4 | 3 | 5 | 4 | 4 |  | 5 | 4 | 5 | 4 | 5 | 5 | 4 | 3 | 4 | 5 | 4 | 4 |
|  | 30 |  |  | 3 | 2 | 3 | 3 | 4 | 3 |  | 4 | 4 | 3 | 5 | 4 | 4 | 3 | 2 | 3 | 3 | 3 | 3 |
|  | 31 |  |  | 3 | 3 | 3 | 2 | 4 | 3 |  | 3 | 3 | 3 | 2 | 4 | 3 | 2 | 2 | 1 | 2 | 3 | 2 |
|  | 32 |  |  | 4 | 4 | 3 | 5 | 4 | 4 |  | 4 | 4 | 3 | 5 | 4 | 4 | 3 | 2 | 3 | 3 | 3 | 3 |
|  | 33 |  |  | 2 | 2 | 1 | 2 | 3 | 2 |  | 3 | 2 | 3 | 4 | 3 | 3 | 2 | 1 | 2 | 3 | 2 | 2 |
|  | 34 |  |  | 3 | 3 | 3 | 2 | 4 | 3 |  | 3 | 2 | 3 | 3 | 3 | 3 | 2 | 1 | 3 | 2 | 2 | 2 |
|  | 35 |  |  | 4 | 4 | 3 | 5 | 4 | 4 |  | 4 | 5 | 3 | 4 | 4 | 4 | 3 | 2 | 3 | 3 | 3 | 3 |
|  | 36 |  |  | 5 | 4 | 5 | 4 | 5 | 5 |  | 4 | 4 | 3 | 5 | 4 | 4 | 3 | 4 | 3 | 3 | 2 | 3 |
|  | 37 |  |  | 4 | 3 | 4 | 4 | 5 | 4 |  | 4 | 3 | 4 | 4 | 5 | 4 | 3 | 3 | 3 | 2 | 4 | 3 |
|  | 38 |  |  | 3 | 3 | 3 | 2 | 4 | 3 |  | 4 | 4 | 3 | 5 | 4 | 4 | 3 | 2 | 3 | 3 | 3 | 3 |
|  | 39 |  |  | 4 | 4 | 3 | 5 | 4 | 4 |  | 5 | 4 | 5 | 4 | 5 | 5 | 4 | 3 | 4 | 5 | 4 | 4 |
|  | 40 |  |  | 5 | 4 | 5 | 4 | 5 | 5 |  | 5 | 4 | 5 | 4 | 5 | 5 | 4 | 3 | 4 | 4 | 5 | 4 |
|  | 41 |  |  | 4 | 3 | 4 | 4 | 5 | 4 |  | 5 | 4 | 5 | 4 | 5 | 5 | 4 | 5 | 3 | 4 | 4 | 4 |
|  | 42 |  |  | 3 | 3 | 3 | 2 | 4 | 3 |  | 4 | 3 | 4 | 4 | 5 | 4 | 3 | 4 | 3 | 3 | 2 | 3 |
|  | 43 |  |  | 4 | 4 | 3 | 5 | 4 | 4 |  | 4 | 4 | 3 | 5 | 4 | 4 | 3 | 3 | 3 | 2 | 4 | 3 |
|  | 44 |  |  | 3 | 3 | 3 | 2 | 4 | 3 |  | 3 | 3 | 3 | 2 | 4 | 3 | 2 | 1 | 2 | 3 | 2 | 2 |
|  | 45 |  |  | 1 | 1 | 1 | 1 | 2 | 1 |  | 2 | 2 | 1 | 2 | 3 | 2 | 1 | 1 | 1 | 1 | 2 | 1 |
|  | 46 |  |  | 4 | 3 | 4 | 4 | 5 | 4 |  | 4 | 3 | 4 | 4 | 5 | 4 | 3 | 4 | 3 | 3 | 2 | 3 |
|  | 47 |  |  | 4 | 4 | 3 | 5 | 4 | 4 |  | 4 | 4 | 3 | 5 | 4 | 4 | 3 | 3 | 3 | 2 | 4 | 3 |
|  | 48 |  |  | 2 | 2 | 1 | 2 | 3 | 2 |  | 3 | 2 | 3 | 4 | 3 | 3 | 2 | 1 | 2 | 3 | 2 | 2 |
|  | 49 |  |  | 3 | 3 | 3 | 2 | 4 | 3 |  | 3 | 2 | 3 | 3 | 3 | 3 | 2 | 1 | 3 | 2 | 2 | 2 |
|  | 50 |  |  | 1 | 1 | 2 | 1 | 2 | 1 |  | 2 | 1 | 3 | 2 | 2 | 2 | 1 | 1 | 1 | 1 | 2 | 1 |
|  | 1 |  | Failure to diagnose the patient quickly | 4 | 3 | 4 | 4 | 5 | 4 | Number Of Corrective Maintenance | 3 | 3 | 3 | 2 | 4 | 3 | 3 | 2 | 3 | 3 | 3 | 3 |
|  | 2 |  |  | 3 | 3 | 3 | 2 | 4 | 3 |  | 2 | 1 | 3 | 2 | 2 | 2 | 2 | 1 | 2 | 3 | 2 | 2 |
|  | 3 |  |  | 2 | 1 | 3 | 2 | 2 | 2 |  | 2 | 2 | 1 | 2 | 3 | 2 | 2 | 1 | 3 | 2 | 2 | 2 |
|  | 4 |  |  | 2 | 3 | 2 | 1 | 2 | 2 |  | 2 | 2 | 1 | 2 | 3 | 2 | 2 | 1 | 2 | 3 | 2 | 2 |
|  | 5 |  |  | 3 | 3 | 3 | 2 | 4 | 3 |  | 2 | 2 | 1 | 2 | 3 | 2 | 2 | 1 | 3 | 2 | 2 | 2 |
|  | 6 |  |  | 4 | 3 | 4 | 4 | 5 | 4 |  | 4 | 4 | 3 | 5 | 4 | 4 | 4 | 3 | 4 | 5 | 4 | 4 |
|  | 7 |  |  | 1 | 1 | 2 | 1 | 2 | 1 |  | 1 | 1 | 1 | 1 | 2 | 1 | 1 | 1 | 1 | 2 | 2 | 1 |
|  | 8 |  |  | 4 | 4 | 3 | 5 | 4 | 4 |  | 4 | 4 | 3 | 5 | 4 | 4 | 4 | 3 | 4 | 5 | 4 | 4 |
|  | 9 |  |  | 3 | 3 | 3 | 2 | 4 | 3 |  | 3 | 3 | 3 | 2 | 4 | 3 | 3 | 2 | 3 | 3 | 3 | 3 |
|  | 10 |  |  | 4 | 4 | 3 | 5 | 4 | 4 |  | 4 | 4 | 3 | 5 | 4 | 4 | 4 | 3 | 4 | 5 | 4 | 4 |
|  | 11 |  |  | 5 | 4 | 5 | 4 | 5 | 5 |  | 5 | 4 | 5 | 4 | 5 | 5 | 4 | 3 | 4 | 4 | 5 | 4 |
|  | 12 |  |  | 4 | 4 | 3 | 5 | 4 | 4 |  | 4 | 4 | 3 | 5 | 4 | 4 | 4 | 5 | 3 | 4 | 4 | 4 |
|  | 13 |  |  | 3 | 3 | 3 | 2 | 4 | 3 |  | 2 | 2 | 1 | 2 | 3 | 2 | 2 | 2 | 1 | 2 | 3 | 2 |
|  | 14 |  |  | 4 | 4 | 3 | 5 | 4 | 4 |  | 4 | 4 | 3 | 5 | 4 | 4 | 4 | 3 | 4 | 5 | 4 | 4 |
|  | 15 |  |  | 3 | 3 | 3 | 2 | 4 | 3 |  | 3 | 3 | 3 | 2 | 4 | 3 | 3 | 2 | 3 | 3 | 3 | 3 |
|  | 16 |  |  | 3 | 2 | 3 | 3 | 4 | 3 |  | 2 | 2 | 1 | 2 | 3 | 2 | 2 | 1 | 2 | 3 | 2 | 2 |
|  | 17 |  |  | 3 | 3 | 3 | 2 | 4 | 3 |  | 2 | 2 | 1 | 2 | 3 | 2 | 2 | 1 | 3 | 2 | 2 | 2 |
|  | 18 |  |  | 2 | 3 | 2 | 1 | 2 | 2 |  | 2 | 1 | 3 | 2 | 2 | 2 | 2 | 2 | 1 | 2 | 3 | 2 |
|  | 19 |  |  | 4 | 3 | 4 | 4 | 5 | 4 |  | 4 | 4 | 3 | 5 | 4 | 4 | 4 | 3 | 4 | 5 | 4 | 4 |
|  | 20 |  |  | 1 | 1 | 1 | 1 | 2 | 1 |  | 1 | 1 | 1 | 1 | 2 | 1 | 1 | 1 | 1 | 1 | 2 | 1 |
|  | 21 |  |  | 2 | 1 | 3 | 2 | 2 | 2 |  | 2 | 2 | 1 | 2 | 3 | 2 | 2 | 1 | 2 | 3 | 2 | 2 |
|  | 22 |  |  | 2 | 3 | 2 | 1 | 2 | 2 |  | 2 | 2 | 1 | 2 | 3 | 2 | 2 | 1 | 3 | 2 | 2 | 2 |
|  | 23 |  |  | 3 | 2 | 3 | 3 | 4 | 3 |  | 2 | 1 | 3 | 2 | 2 | 2 | 2 | 2 | 1 | 2 | 3 | 2 |
|  | 24 |  |  | 3 | 3 | 3 | 2 | 4 | 3 |  | 3 | 3 | 3 | 2 | 4 | 3 | 3 | 2 | 3 | 3 | 3 | 3 |
|  | 25 |  |  | 2 | 2 | 1 | 2 | 3 | 2 |  | 2 | 1 | 3 | 2 | 2 | 2 | 2 | 1 | 2 | 3 | 2 | 2 |
|  | 26 |  |  | 3 | 3 | 3 | 2 | 4 | 3 |  | 2 | 1 | 3 | 2 | 2 | 2 | 2 | 1 | 3 | 2 | 2 | 2 |
|  | 27 |  |  | 4 | 4 | 3 | 5 | 4 | 4 |  | 5 | 4 | 5 | 4 | 5 | 5 | 4 | 3 | 4 | 5 | 4 | 4 |
|  | 28 |  |  | 2 | 2 | 1 | 2 | 3 | 2 |  | 2 | 1 | 3 | 2 | 2 | 2 | 2 | 2 | 1 | 2 | 3 | 2 |
|  | 29 |  |  | 3 | 2 | 3 | 3 | 4 | 3 |  | 4 | 4 | 3 | 5 | 4 | 4 | 4 | 3 | 4 | 5 | 4 | 4 |
|  | 30 |  |  | 3 | 3 | 3 | 2 | 4 | 3 |  | 2 | 2 | 1 | 2 | 3 | 2 | 2 | 1 | 2 | 3 | 2 | 2 |
|  | 31 |  |  | 3 | 2 | 3 | 3 | 4 | 3 |  | 3 | 3 | 3 | 2 | 4 | 3 | 3 | 2 | 3 | 3 | 3 | 3 |
|  | 32 |  |  | 3 | 3 | 3 | 2 | 4 | 3 |  | 2 | 2 | 1 | 2 | 3 | 2 | 2 | 1 | 2 | 3 | 2 | 2 |
|  | 33 |  |  | 2 | 2 | 1 | 2 | 3 | 2 |  | 2 | 2 | 1 | 2 | 3 | 2 | 2 | 1 | 3 | 2 | 2 | 2 |
|  | 34 |  |  | 1 | 1 | 1 | 1 | 2 | 1 |  | 1 | 1 | 1 | 1 | 2 | 1 | 1 | 1 | 1 | 1 | 2 | 1 |
|  | 35 |  |  | 3 | 3 | 3 | 2 | 4 | 3 |  | 2 | 2 | 1 | 2 | 3 | 2 | 2 | 1 | 2 | 3 | 2 | 2 |
|  | 36 |  |  | 2 | 3 | 2 | 1 | 2 | 2 |  | 2 | 2 | 1 | 2 | 3 | 2 | 2 | 1 | 3 | 2 | 2 | 2 |
|  | 37 |  |  | 3 | 3 | 3 | 2 | 4 | 3 |  | 3 | 3 | 3 | 2 | 4 | 3 | 3 | 2 | 3 | 3 | 3 | 3 |
|  | 38 |  |  | 4 | 3 | 4 | 4 | 5 | 4 |  | 4 | 4 | 3 | 5 | 4 | 4 | 4 | 3 | 4 | 5 | 4 | 4 |
|  | 39 |  |  | 3 | 3 | 3 | 2 | 4 | 3 |  | 2 | 1 | 3 | 2 | 2 | 2 | 2 | 2 | 1 | 2 | 3 | 2 |
|  | 40 |  |  | 5 | 4 | 5 | 4 | 5 | 5 |  | 5 | 4 | 5 | 4 | 5 | 5 | 4 | 3 | 4 | 5 | 4 | 4 |
|  | 41 |  |  | 3 | 3 | 3 | 2 | 4 | 3 |  | 2 | 2 | 1 | 2 | 3 | 2 | 2 | 1 | 2 | 3 | 2 | 2 |
|  | 42 |  |  | 2 | 2 | 1 | 2 | 3 | 2 |  | 2 | 2 | 1 | 2 | 3 | 2 | 2 | 1 | 3 | 2 | 2 | 2 |
|  | 43 |  |  | 3 | 3 | 3 | 2 | 4 | 3 |  | 3 | 3 | 3 | 2 | 4 | 3 | 3 | 2 | 3 | 3 | 3 | 3 |
|  | 44 |  |  | 1 | 1 | 1 | 1 | 2 | 1 |  | 1 | 1 | 1 | 1 | 2 | 1 | 1 | 1 | 2 | 1 | 2 | 1 |
|  | 45 |  |  | 1 | 1 | 1 | 1 | 2 | 1 |  | 1 | 1 | 1 | 1 | 2 | 1 | 1 | 1 | 1 | 1 | 2 | 1 |
|  | 46 |  |  | 3 | 3 | 3 | 2 | 4 | 3 |  | 2 | 2 | 1 | 2 | 3 | 2 | 2 | 1 | 2 | 3 | 2 | 2 |
|  | 47 |  |  | 2 | 2 | 1 | 2 | 3 | 2 |  | 2 | 2 | 1 | 2 | 3 | 2 | 2 | 1 | 3 | 2 | 2 | 2 |
|  | 48 |  |  | 4 | 5 | 3 | 4 | 4 | 4 |  | 4 | 4 | 3 | 5 | 4 | 4 | 4 | 3 | 4 | 5 | 4 | 4 |
|  | 49 |  |  | 3 | 3 | 3 | 2 | 4 | 3 |  | 2 | 1 | 3 | 2 | 2 | 2 | 2 | 1 | 2 | 3 | 2 | 2 |
|  | 50 |  |  | 2 | 1 | 1 | 1 | 2 | 2 |  | 1 | 1 | 1 | 1 | 2 | 1 | 1 | 2 | 1 | 1 | 2 | 1 |
|  | 1 | The results of the tests are not accurate | Misdiagnosis of the patient's condition | 4 | 3 | 4 | 4 | 5 | 4 | Response time for visit in Hour | 4 | 4 | 3 | 5 | 4 | 4 | 5 | 4 | 5 | 4 | 5 | 5 |
|  | 2 |  |  | 2 | 2 | 1 | 2 | 3 | 2 |  | 2 | 2 | 1 | 2 | 3 | 2 | 3 | 2 | 3 | 3 | 3 | 3 |
|  | 3 |  |  | 3 | 3 | 3 | 2 | 4 | 3 |  | 3 | 3 | 3 | 2 | 4 | 3 | 4 | 3 | 4 | 5 | 4 | 4 |
|  | 4 |  |  | 2 | 2 | 1 | 2 | 3 | 2 |  | 2 | 2 | 1 | 2 | 3 | 2 | 3 | 4 | 3 | 3 | 2 | 3 |
|  | 5 |  |  | 3 | 3 | 3 | 2 | 4 | 3 |  | 3 | 3 | 3 | 2 | 4 | 3 | 4 | 3 | 4 | 5 | 4 | 4 |
|  | 6 |  |  | 4 | 4 | 3 | 5 | 4 | 4 |  | 5 | 4 | 5 | 4 | 5 | 5 | 5 | 4 | 5 | 4 | 5 | 5 |
|  | 7 |  |  | 2 | 2 | 1 | 2 | 3 | 2 |  | 2 | 2 | 1 | 2 | 3 | 2 | 3 | 4 | 3 | 3 | 2 | 3 |
|  | 8 |  |  | 1 | 1 | 1 | 1 | 2 | 1 |  | 1 | 1 | 1 | 1 | 2 | 1 | 3 | 3 | 3 | 2 | 4 | 3 |
|  | 9 |  |  | 2 | 1 | 3 | 2 | 2 | 2 |  | 2 | 2 | 1 | 2 | 3 | 2 | 3 | 2 | 3 | 3 | 3 | 3 |
|  | 10 |  |  | 2 | 3 | 2 | 1 | 2 | 2 |  | 2 | 2 | 1 | 2 | 3 | 2 | 3 | 4 | 3 | 3 | 2 | 3 |
|  | 11 |  |  | 2 | 2 | 1 | 2 | 3 | 2 |  | 3 | 3 | 3 | 2 | 4 | 3 | 3 | 3 | 3 | 2 | 4 | 3 |
|  | 12 |  |  | 4 | 5 | 3 | 4 | 4 | 4 |  | 4 | 3 | 4 | 4 | 5 | 4 | 4 | 3 | 4 | 5 | 4 | 4 |
|  | 13 |  |  | 4 | 4 | 3 | 5 | 4 | 4 |  | 4 | 5 | 3 | 4 | 4 | 4 | 2 | 2 | 1 | 2 | 3 | 2 |
|  | 14 |  |  | 4 | 3 | 4 | 4 | 5 | 4 |  | 4 | 4 | 3 | 5 | 4 | 4 | 3 | 2 | 3 | 3 | 3 | 3 |
|  | 15 |  |  | 4 | 5 | 3 | 4 | 4 | 4 |  | 5 | 4 | 5 | 4 | 5 | 5 | 5 | 4 | 5 | 4 | 5 | 5 |
|  | 16 |  |  | 2 | 2 | 1 | 2 | 3 | 2 |  | 2 | 2 | 1 | 2 | 3 | 2 | 3 | 4 | 3 | 3 | 2 | 3 |
|  | 17 |  |  | 2 | 1 | 3 | 2 | 2 | 2 |  | 2 | 2 | 1 | 2 | 3 | 2 | 3 | 3 | 3 | 2 | 4 | 3 |
|  | 18 |  |  | 2 | 3 | 2 | 1 | 2 | 2 |  | 2 | 2 | 1 | 2 | 3 | 2 | 3 | 2 | 3 | 3 | 3 | 3 |
|  | 19 |  |  | 2 | 2 | 1 | 2 | 3 | 2 |  | 2 | 1 | 3 | 2 | 2 | 2 | 3 | 4 | 3 | 3 | 2 | 3 |
|  | 20 |  |  | 2 | 2 | 1 | 2 | 3 | 2 |  | 2 | 3 | 2 | 1 | 2 | 2 | 3 | 3 | 3 | 2 | 4 | 3 |
|  | 21 |  |  | 4 | 3 | 4 | 4 | 5 | 4 |  | 5 | 4 | 5 | 4 | 5 | 5 | 5 | 4 | 5 | 4 | 5 | 5 |
|  | 22 |  |  | 3 | 3 | 3 | 2 | 4 | 3 |  | 3 | 3 | 3 | 2 | 4 | 3 | 4 | 3 | 4 | 5 | 4 | 4 |
|  | 23 |  |  | 3 | 3 | 3 | 2 | 4 | 3 |  | 3 | 3 | 3 | 2 | 4 | 3 | 4 | 3 | 4 | 4 | 5 | 4 |
|  | 24 |  |  | 3 | 3 | 3 | 2 | 4 | 3 |  | 3 | 3 | 3 | 2 | 4 | 3 | 4 | 5 | 3 | 4 | 4 | 4 |
|  | 25 |  |  | 4 | 5 | 3 | 4 | 4 | 4 |  | 4 | 3 | 4 | 4 | 5 | 4 | 5 | 4 | 5 | 4 | 5 | 5 |
|  | 26 |  |  | 2 | 1 | 3 | 2 | 2 | 2 |  | 3 | 3 | 3 | 2 | 4 | 3 | 3 | 4 | 3 | 3 | 2 | 3 |
|  | 27 |  |  | 2 | 2 | 1 | 2 | 3 | 2 |  | 2 | 2 | 1 | 2 | 3 | 2 | 3 | 3 | 3 | 2 | 4 | 3 |
|  | 28 |  |  | 3 | 3 | 3 | 2 | 4 | 3 |  | 3 | 3 | 3 | 2 | 4 | 3 | 4 | 3 | 4 | 4 | 5 | 4 |
|  | 29 |  |  | 3 | 3 | 3 | 2 | 4 | 3 |  | 3 | 3 | 3 | 2 | 4 | 3 | 4 | 5 | 3 | 4 | 4 | 4 |
|  | 30 |  |  | 2 | 2 | 1 | 2 | 3 | 2 |  | 3 | 3 | 3 | 2 | 4 | 3 | 3 | 2 | 3 | 3 | 3 | 3 |
|  | 31 |  |  | 4 | 3 | 4 | 4 | 5 | 4 |  | 4 | 3 | 4 | 4 | 5 | 4 | 5 | 4 | 5 | 4 | 5 | 5 |
|  | 32 |  |  | 3 | 3 | 3 | 2 | 4 | 3 |  | 3 | 3 | 3 | 2 | 4 | 3 | 4 | 3 | 4 | 5 | 4 | 4 |
|  | 33 |  |  | 4 | 4 | 3 | 5 | 4 | 4 |  | 4 | 3 | 4 | 4 | 5 | 4 | 5 | 4 | 5 | 4 | 5 | 5 |
|  | 34 |  |  | 4 | 3 | 4 | 4 | 5 | 4 |  | 4 | 3 | 4 | 4 | 5 | 4 | 5 | 5 | 4 | 4 | 5 | 5 |
|  | 35 |  |  | 1 | 1 | 1 | 1 | 2 | 1 |  | 2 | 2 | 1 | 2 | 3 | 2 | 3 | 2 | 3 | 3 | 3 | 3 |
|  | 36 |  |  | 1 | 1 | 1 | 1 | 2 | 1 |  | 2 | 2 | 1 | 2 | 3 | 2 | 3 | 4 | 3 | 3 | 2 | 3 |
|  | 37 |  |  | 1 | 1 | 1 | 1 | 2 | 1 |  | 1 | 1 | 1 | 1 | 2 | 1 | 3 | 3 | 3 | 2 | 4 | 3 |
|  | 38 |  |  | 3 | 2 | 4 | 3 | 3 | 3 |  | 3 | 2 | 4 | 3 | 3 | 3 | 4 | 3 | 4 | 5 | 4 | 4 |
|  | 39 |  |  | 3 | 3 | 3 | 2 | 4 | 3 |  | 3 | 3 | 3 | 2 | 4 | 3 | 4 | 3 | 4 | 4 | 5 | 4 |
|  | 40 |  |  | 3 | 4 | 3 | 3 | 2 | 3 |  | 3 | 4 | 3 | 3 | 2 | 3 | 4 | 5 | 3 | 4 | 4 | 4 |
|  | 41 |  |  | 3 | 3 | 3 | 2 | 4 | 3 |  | 3 | 3 | 3 | 2 | 4 | 3 | 4 | 3 | 4 | 5 | 4 | 4 |
|  | 42 |  |  | 4 | 4 | 3 | 5 | 4 | 4 |  | 5 | 4 | 5 | 4 | 5 | 5 | 5 | 4 | 5 | 4 | 5 | 5 |
|  | 43 |  |  | 4 | 3 | 4 | 4 | 5 | 4 |  | 4 | 3 | 4 | 4 | 5 | 4 | 5 | 5 | 4 | 4 | 5 | 5 |
|  | 44 |  |  | 3 | 2 | 4 | 3 | 3 | 3 |  | 3 | 3 | 3 | 2 | 4 | 3 | 4 | 3 | 4 | 5 | 4 | 4 |
|  | 45 |  |  | 1 | 1 | 1 | 1 | 2 | 1 |  | 2 | 2 | 1 | 2 | 3 | 2 | 3 | 4 | 3 | 3 | 2 | 3 |
|  | 46 |  |  | 1 | 1 | 1 | 1 | 2 | 1 |  | 2 | 2 | 1 | 2 | 3 | 2 | 3 | 3 | 3 | 2 | 4 | 3 |
|  | 47 |  |  | 3 | 3 | 4 | 2 | 3 | 3 |  | 4 | 3 | 4 | 4 | 5 | 4 | 5 | 4 | 5 | 4 | 5 | 5 |
|  | 48 |  |  | 3 | 2 | 4 | 3 | 3 | 3 |  | 3 | 3 | 3 | 2 | 4 | 3 | 4 | 3 | 4 | 5 | 4 | 4 |
|  | 49 |  |  | 1 | 1 | 1 | 1 | 2 | 1 |  | 1 | 1 | 1 | 1 | 2 | 1 | 3 | 4 | 3 | 3 | 2 | 3 |
|  | 50 |  |  | 2 | 2 | 1 | 2 | 3 | 2 |  | 2 | 2 | 1 | 2 | 3 | 2 | 3 | 3 | 3 | 2 | 4 | 3 |

Survey for chemical analyzer

| Experts ratings for S, P, and D for chemical analyzers | | | | | | | | | | | | | | | | | | | | | | |
| --- | --- | --- | --- | --- | --- | --- | --- | --- | --- | --- | --- | --- | --- | --- | --- | --- | --- | --- | --- | --- | --- | --- |
|  |  |  |  | Experts Severity | | | | |  | Experts Probability | | | | | Experts Detection | | | | |  |  |  |
| Function | Device no. | Failure mode | Effects of Failure | E1 | E2 | E3 | E4 | E5 | Average | Causes of Failure | E1 | E2 | E3 | E4 | E5 | Average | E1 | E2 | E3 | E4 | E5 | Average |
| Accurately diagnose the patient's condition | 1 | Some devices are not working | pressure on working devices | 5 | 4 | 5 | 4 | 5 | 5 | number of complaints | 5 | 4 | 5 | 4 | 5 | 5 | 2 | 1 | 3 | 2 | 2 | 2 |
|  | 2 |  |  | 4 | 3 | 4 | 4 | 5 | 4 |  | 3 | 3 | 3 | 2 | 4 | 3 | 2 | 2 | 1 | 2 | 3 | 2 |
|  | 3 |  |  | 5 | 4 | 5 | 4 | 5 | 5 |  | 5 | 4 | 5 | 4 | 5 | 5 | 3 | 2 | 4 | 3 | 3 | 3 |
|  | 4 |  |  | 4 | 3 | 4 | 4 | 5 | 4 |  | 4 | 3 | 4 | 4 | 5 | 4 | 3 | 3 | 3 | 2 | 4 | 3 |
|  | 5 |  |  | 5 | 4 | 5 | 4 | 5 | 5 |  | 5 | 4 | 5 | 4 | 5 | 5 | 2 | 2 | 1 | 2 | 3 | 2 |
|  | 6 |  |  | 4 | 3 | 4 | 4 | 5 | 4 |  | 4 | 3 | 4 | 4 | 5 | 4 | 3 | 3 | 3 | 2 | 4 | 3 |
|  | 7 |  |  | 4 | 5 | 3 | 4 | 4 | 4 |  | 3 | 2 | 4 | 3 | 3 | 3 | 2 | 1 | 3 | 2 | 2 | 2 |
|  | 8 |  |  | 3 | 3 | 3 | 2 | 4 | 3 |  | 3 | 3 | 3 | 2 | 4 | 3 | 2 | 2 | 1 | 2 | 3 | 2 |
|  | 9 |  |  | 1 | 1 | 1 | 1 | 2 | 1 |  | 1 | 1 | 1 | 1 | 2 | 1 | 1 | 1 | 1 | 1 | 2 | 1 |
|  | 10 |  |  | 5 | 4 | 5 | 4 | 5 | 5 |  | 5 | 4 | 5 | 4 | 5 | 5 | 2 | 2 | 1 | 2 | 3 | 2 |
|  | 11 |  |  | 1 | 1 | 1 | 1 | 2 | 1 |  | 1 | 1 | 1 | 1 | 2 | 1 | 3 | 3 | 3 | 2 | 4 | 3 |
|  | 12 |  |  | 4 | 3 | 4 | 4 | 5 | 4 |  | 4 | 3 | 4 | 4 | 5 | 4 | 2 | 2 | 1 | 2 | 3 | 2 |
|  | 13 |  |  | 5 | 4 | 5 | 4 | 5 | 5 |  | 5 | 4 | 5 | 4 | 5 | 5 | 3 | 3 | 3 | 2 | 4 | 3 |
|  | 14 |  |  | 5 | 5 | 4 | 4 | 5 | 5 |  | 5 | 5 | 4 | 4 | 5 | 5 | 2 | 1 | 3 | 2 | 2 | 2 |
|  | 15 |  |  | 1 | 1 | 1 | 1 | 2 | 1 |  | 1 | 1 | 1 | 1 | 2 | 1 | 2 | 2 | 1 | 2 | 3 | 2 |
|  | 16 |  |  | 4 | 3 | 4 | 4 | 5 | 4 |  | 3 | 2 | 4 | 3 | 3 | 3 | 3 | 3 | 3 | 2 | 4 | 3 |
|  | 17 |  |  | 3 | 3 | 3 | 2 | 4 | 3 |  | 3 | 3 | 3 | 2 | 4 | 3 | 2 | 1 | 3 | 2 | 2 | 2 |
|  | 18 |  |  | 2 | 1 | 3 | 2 | 2 | 2 |  | 2 | 2 | 1 | 2 | 3 | 2 | 2 | 2 | 1 | 2 | 3 | 2 |
|  | 19 |  |  | 2 | 2 | 1 | 2 | 3 | 2 |  | 1 | 1 | 1 | 1 | 2 | 1 | 1 | 1 | 1 | 1 | 2 | 1 |
|  | 20 |  |  | 3 | 2 | 4 | 3 | 3 | 3 |  | 3 | 2 | 4 | 3 | 3 | 3 | 2 | 1 | 3 | 2 | 2 | 2 |
|  | 21 |  |  | 3 | 3 | 3 | 2 | 4 | 3 |  | 3 | 3 | 3 | 2 | 4 | 3 | 2 | 1 | 3 | 2 | 2 | 2 |
|  | 22 |  |  | 4 | 5 | 3 | 4 | 4 | 4 |  | 3 | 2 | 4 | 3 | 3 | 3 | 2 | 2 | 1 | 2 | 3 | 2 |
|  | 23 |  |  | 3 | 3 | 3 | 2 | 4 | 3 |  | 3 | 3 | 3 | 2 | 4 | 3 | 3 | 3 | 3 | 2 | 4 | 3 |
|  | 24 |  |  | 2 | 1 | 3 | 2 | 2 | 2 |  | 2 | 2 | 1 | 2 | 3 | 2 | 3 | 2 | 4 | 3 | 3 | 3 |
|  | 25 |  |  | 3 | 2 | 4 | 3 | 3 | 3 |  | 3 | 2 | 4 | 3 | 3 | 3 | 2 | 2 | 1 | 2 | 3 | 2 |
|  | 26 |  |  | 3 | 3 | 3 | 2 | 4 | 3 |  | 3 | 3 | 3 | 2 | 4 | 3 | 3 | 3 | 3 | 2 | 4 | 3 |
|  | 27 |  |  | 4 | 5 | 3 | 4 | 4 | 4 |  | 3 | 2 | 4 | 3 | 3 | 3 | 2 | 2 | 1 | 2 | 3 | 2 |
|  | 28 |  |  | 3 | 3 | 3 | 2 | 4 | 3 |  | 3 | 3 | 3 | 2 | 4 | 3 | 3 | 3 | 3 | 2 | 4 | 3 |
|  | 29 |  |  | 4 | 3 | 4 | 4 | 5 | 4 |  | 4 | 5 | 3 | 4 | 4 | 4 | 2 | 1 | 3 | 2 | 2 | 2 |
|  | 30 |  |  | 5 | 4 | 5 | 4 | 5 | 5 |  | 5 | 4 | 5 | 4 | 5 | 5 | 2 | 2 | 1 | 2 | 3 | 2 |
|  | 31 |  |  | 5 | 5 | 4 | 4 | 5 | 5 |  | 5 | 5 | 4 | 4 | 5 | 5 | 2 | 1 | 3 | 2 | 2 | 2 |
|  | 32 |  |  | 4 | 3 | 4 | 4 | 5 | 4 |  | 4 | 3 | 4 | 4 | 5 | 4 | 2 | 2 | 1 | 2 | 3 | 2 |
|  | 33 |  |  | 3 | 3 | 3 | 2 | 4 | 3 |  | 3 | 3 | 3 | 2 | 4 | 3 | 3 | 3 | 3 | 2 | 4 | 3 |
|  | 34 |  |  | 5 | 4 | 5 | 4 | 5 | 5 |  | 5 | 4 | 5 | 4 | 5 | 5 | 2 | 2 | 1 | 2 | 3 | 2 |
|  | 35 |  |  | 5 | 5 | 4 | 4 | 5 | 5 |  | 5 | 5 | 4 | 4 | 5 | 5 | 3 | 3 | 3 | 2 | 4 | 3 |
|  | 36 |  |  | 4 | 5 | 3 | 4 | 4 | 4 |  | 3 | 3 | 3 | 2 | 4 | 3 | 3 | 2 | 4 | 3 | 3 | 3 |
|  | 37 |  |  | 4 | 5 | 3 | 4 | 4 | 4 |  | 4 | 3 | 4 | 4 | 5 | 4 | 2 | 1 | 3 | 2 | 2 | 2 |
|  | 38 |  |  | 4 | 4 | 3 | 5 | 4 | 4 |  | 3 | 2 | 4 | 3 | 3 | 3 | 2 | 2 | 1 | 2 | 3 | 2 |
|  | 39 |  |  | 3 | 3 | 3 | 2 | 4 | 3 |  | 3 | 3 | 3 | 2 | 4 | 3 | 2 | 2 | 1 | 2 | 3 | 2 |
|  | 40 |  |  | 2 | 1 | 3 | 2 | 2 | 2 |  | 2 | 2 | 1 | 2 | 3 | 2 | 1 | 1 | 1 | 1 | 2 | 1 |
|  | 41 |  |  | 4 | 3 | 4 | 4 | 5 | 4 |  | 3 | 2 | 4 | 3 | 3 | 3 | 2 | 1 | 3 | 2 | 2 | 2 |
|  | 42 |  |  | 3 | 3 | 3 | 2 | 4 | 3 |  | 3 | 3 | 3 | 2 | 4 | 3 | 2 | 1 | 3 | 2 | 2 | 2 |
|  | 43 |  |  | 4 | 5 | 3 | 4 | 4 | 4 |  | 3 | 2 | 4 | 3 | 3 | 3 | 2 | 2 | 1 | 2 | 3 | 2 |
|  | 44 |  |  | 4 | 4 | 3 | 5 | 4 | 4 |  | 3 | 3 | 3 | 2 | 4 | 3 | 3 | 2 | 4 | 3 | 3 | 3 |
|  | 45 |  |  | 5 | 4 | 5 | 4 | 5 | 5 |  | 5 | 4 | 5 | 4 | 5 | 5 | 3 | 3 | 3 | 2 | 4 | 3 |
|  | 46 |  |  | 1 | 1 | 1 | 1 | 2 | 1 |  | 1 | 1 | 1 | 1 | 2 | 1 | 2 | 2 | 1 | 2 | 3 | 2 |
|  | 47 |  |  | 1 | 1 | 1 | 1 | 2 | 1 |  | 1 | 1 | 1 | 1 | 2 | 1 | 1 | 1 | 1 | 1 | 2 | 1 |
|  | 48 |  |  | 4 | 3 | 4 | 4 | 5 | 4 |  | 4 | 3 | 4 | 4 | 5 | 4 | 2 | 2 | 1 | 2 | 3 | 2 |
|  | 49 |  |  | 1 | 1 | 1 | 1 | 2 | 1 |  | 1 | 1 | 1 | 1 | 2 | 1 | 1 | 1 | 1 | 1 | 2 | 1 |
|  | 50 |  |  | 5 | 4 | 5 | 4 | 5 | 5 |  | 5 | 4 | 5 | 4 | 5 | 5 | 3 | 3 | 3 | 2 | 4 | 3 |
|  | 1 |  | Inaccuracy of the results | 5 | 5 | 4 | 4 | 5 | 5 | Quality Control | 5 | 5 | 4 | 4 | 5 | 5 | 1 | 1 | 1 | 1 | 2 | 1 |
|  | 2 |  |  | 2 | 1 | 3 | 2 | 2 | 2 |  | 3 | 3 | 3 | 2 | 4 | 3 | 2 | 2 | 1 | 2 | 3 | 2 |
|  | 3 |  |  | 5 | 4 | 5 | 4 | 5 | 5 |  | 5 | 4 | 5 | 4 | 5 | 5 | 1 | 1 | 1 | 1 | 2 | 1 |
|  | 4 |  |  | 2 | 1 | 3 | 2 | 2 | 2 |  | 3 | 3 | 3 | 2 | 4 | 3 | 2 | 2 | 1 | 2 | 3 | 2 |
|  | 5 |  |  | 4 | 3 | 4 | 4 | 5 | 4 |  | 4 | 3 | 4 | 4 | 5 | 4 | 1 | 1 | 1 | 1 | 2 | 1 |
|  | 6 |  |  | 2 | 1 | 3 | 2 | 2 | 2 |  | 3 | 2 | 4 | 3 | 3 | 3 | 2 | 1 | 3 | 2 | 2 | 2 |
|  | 7 |  |  | 2 | 2 | 1 | 2 | 3 | 2 |  | 3 | 3 | 3 | 2 | 4 | 3 | 2 | 2 | 1 | 2 | 3 | 2 |
|  | 8 |  |  | 2 | 1 | 3 | 2 | 2 | 2 |  | 3 | 2 | 4 | 3 | 3 | 3 | 2 | 1 | 3 | 2 | 2 | 2 |
|  | 9 |  |  | 2 | 2 | 1 | 2 | 3 | 2 |  | 3 | 3 | 3 | 2 | 4 | 3 | 2 | 2 | 1 | 2 | 3 | 2 |
|  | 10 |  |  | 2 | 1 | 3 | 2 | 2 | 2 |  | 3 | 2 | 4 | 3 | 3 | 3 | 2 | 1 | 3 | 2 | 2 | 2 |
|  | 11 |  |  | 2 | 2 | 1 | 2 | 3 | 2 |  | 3 | 3 | 3 | 2 | 4 | 3 | 2 | 1 | 3 | 2 | 2 | 2 |
|  | 12 |  |  | 4 | 3 | 4 | 4 | 5 | 4 |  | 4 | 3 | 4 | 4 | 5 | 4 | 2 | 2 | 1 | 2 | 3 | 2 |
|  | 13 |  |  | 5 | 4 | 5 | 4 | 5 | 5 |  | 5 | 4 | 5 | 4 | 5 | 5 | 1 | 1 | 1 | 1 | 2 | 1 |
|  | 14 |  |  | 2 | 1 | 3 | 2 | 2 | 2 |  | 3 | 2 | 4 | 3 | 3 | 3 | 2 | 1 | 3 | 2 | 2 | 2 |
|  | 15 |  |  | 2 | 1 | 3 | 2 | 2 | 2 |  | 3 | 3 | 3 | 2 | 4 | 3 | 2 | 2 | 1 | 2 | 3 | 2 |
|  | 16 |  |  | 2 | 2 | 1 | 2 | 3 | 2 |  | 3 | 2 | 4 | 3 | 3 | 3 | 2 | 1 | 3 | 2 | 2 | 2 |
|  | 17 |  |  | 2 | 1 | 3 | 2 | 2 | 2 |  | 3 | 3 | 3 | 2 | 4 | 3 | 2 | 2 | 1 | 2 | 3 | 2 |
|  | 18 |  |  | 2 | 2 | 1 | 2 | 3 | 2 |  | 3 | 2 | 4 | 3 | 3 | 3 | 2 | 1 | 3 | 2 | 2 | 2 |
|  | 19 |  |  | 2 | 1 | 3 | 2 | 2 | 2 |  | 3 | 3 | 3 | 2 | 4 | 3 | 2 | 1 | 3 | 2 | 2 | 2 |
|  | 20 |  |  | 2 | 2 | 1 | 2 | 3 | 2 |  | 3 | 3 | 3 | 2 | 4 | 3 | 2 | 2 | 1 | 2 | 3 | 2 |
|  | 21 |  |  | 4 | 5 | 3 | 4 | 4 | 4 |  | 4 | 5 | 3 | 4 | 4 | 4 | 1 | 1 | 1 | 1 | 2 | 1 |
|  | 22 |  |  | 4 | 4 | 3 | 5 | 4 | 4 |  | 4 | 4 | 3 | 5 | 4 | 4 | 1 | 1 | 1 | 1 | 2 | 1 |
|  | 23 |  |  | 5 | 4 | 5 | 4 | 5 | 5 |  | 5 | 4 | 5 | 4 | 5 | 5 | 1 | 1 | 1 | 1 | 2 | 1 |
|  | 24 |  |  | 2 | 1 | 3 | 2 | 2 | 2 |  | 3 | 3 | 3 | 2 | 4 | 3 | 1 | 1 | 1 | 1 | 2 | 1 |
|  | 25 |  |  | 4 | 5 | 3 | 4 | 4 | 4 |  | 4 | 5 | 3 | 4 | 4 | 4 | 1 | 1 | 1 | 1 | 2 | 1 |
|  | 26 |  |  | 4 | 4 | 3 | 5 | 4 | 4 |  | 4 | 4 | 3 | 5 | 4 | 4 | 1 | 1 | 1 | 1 | 2 | 1 |
|  | 27 |  |  | 2 | 1 | 3 | 2 | 2 | 2 |  | 3 | 2 | 4 | 3 | 3 | 3 | 2 | 1 | 3 | 2 | 2 | 2 |
|  | 28 |  |  | 2 | 2 | 1 | 2 | 3 | 2 |  | 3 | 3 | 3 | 2 | 4 | 3 | 2 | 2 | 1 | 2 | 3 | 2 |
|  | 29 |  |  | 2 | 2 | 1 | 2 | 3 | 2 |  | 3 | 3 | 3 | 2 | 4 | 3 | 2 | 1 | 3 | 2 | 2 | 2 |
|  | 30 |  |  | 4 | 3 | 4 | 4 | 5 | 4 |  | 2 | 2 | 1 | 2 | 3 | 2 | 2 | 2 | 1 | 2 | 3 | 2 |
|  | 31 |  |  | 5 | 4 | 5 | 4 | 5 | 5 |  | 5 | 4 | 5 | 4 | 5 | 5 | 2 | 1 | 3 | 2 | 2 | 2 |
|  | 32 |  |  | 2 | 1 | 3 | 2 | 2 | 2 |  | 3 | 2 | 4 | 3 | 3 | 3 | 2 | 2 | 1 | 2 | 3 | 2 |
|  | 33 |  |  | 2 | 2 | 1 | 2 | 3 | 2 |  | 3 | 3 | 3 | 2 | 4 | 3 | 2 | 1 | 3 | 2 | 2 | 2 |
|  | 34 |  |  | 2 | 1 | 3 | 2 | 2 | 2 |  | 3 | 2 | 4 | 3 | 3 | 3 | 2 | 2 | 1 | 2 | 3 | 2 |
|  | 35 |  |  | 2 | 2 | 1 | 2 | 3 | 2 |  | 3 | 3 | 3 | 2 | 4 | 3 | 2 | 1 | 3 | 2 | 2 | 2 |
|  | 36 |  |  | 5 | 4 | 5 | 4 | 5 | 5 |  | 5 | 4 | 5 | 4 | 5 | 5 | 2 | 2 | 1 | 2 | 3 | 2 |
|  | 37 |  |  | 2 | 1 | 3 | 2 | 2 | 2 |  | 3 | 2 | 4 | 3 | 3 | 3 | 2 | 1 | 3 | 2 | 2 | 2 |
|  | 38 |  |  | 3 | 3 | 3 | 2 | 4 | 3 |  | 3 | 2 | 4 | 3 | 3 | 3 | 2 | 2 | 1 | 2 | 3 | 2 |
|  | 39 |  |  | 2 | 1 | 3 | 2 | 2 | 2 |  | 3 | 3 | 3 | 2 | 4 | 3 | 2 | 1 | 3 | 2 | 2 | 2 |
|  | 40 |  |  | 2 | 1 | 2 | 2 | 3 | 2 |  | 3 | 2 | 4 | 3 | 3 | 3 | 2 | 2 | 1 | 2 | 3 | 2 |
|  | 41 |  |  | 3 | 3 | 3 | 2 | 4 | 3 |  | 3 | 3 | 3 | 2 | 4 | 3 | 2 | 1 | 3 | 2 | 2 | 2 |
|  | 42 |  |  | 2 | 1 | 2 | 2 | 3 | 2 |  | 3 | 2 | 4 | 3 | 3 | 3 | 2 | 2 | 1 | 2 | 3 | 2 |
|  | 43 |  |  | 3 | 3 | 3 | 2 | 4 | 3 |  | 3 | 3 | 3 | 2 | 4 | 3 | 2 | 1 | 3 | 2 | 2 | 2 |
|  | 44 |  |  | 4 | 3 | 4 | 4 | 5 | 4 |  | 3 | 3 | 3 | 2 | 4 | 3 | 2 | 2 | 1 | 2 | 3 | 2 |
|  | 45 |  |  | 2 | 1 | 3 | 2 | 2 | 2 |  | 3 | 2 | 4 | 3 | 3 | 3 | 2 | 1 | 3 | 2 | 2 | 2 |
|  | 46 |  |  | 2 | 2 | 1 | 2 | 3 | 2 |  | 3 | 3 | 3 | 2 | 4 | 3 | 2 | 2 | 1 | 2 | 3 | 2 |
|  | 47 |  |  | 2 | 1 | 3 | 2 | 2 | 2 |  | 3 | 2 | 4 | 3 | 3 | 3 | 2 | 1 | 3 | 2 | 2 | 2 |
|  | 48 |  |  | 2 | 1 | 3 | 2 | 2 | 2 |  | 3 | 2 | 4 | 3 | 3 | 3 | 2 | 1 | 3 | 2 | 2 | 2 |
|  | 49 |  |  | 2 | 2 | 1 | 2 | 3 | 2 |  | 3 | 3 | 3 | 2 | 4 | 3 | 2 | 2 | 1 | 2 | 3 | 2 |
|  | 50 |  |  | 5 | 4 | 5 | 4 | 5 | 5 |  | 5 | 4 | 5 | 4 | 5 | 5 | 2 | 1 | 3 | 2 | 2 | 2 |
|  | 1 | Delayed test result | Patient dissatisfaction | 4 | 5 | 3 | 4 | 4 | 4 | Number Of Preventive Maintenance | 4 | 5 | 3 | 4 | 4 | 4 | 2 | 2 | 1 | 2 | 3 | 2 |
|  | 2 |  |  | 4 | 4 | 3 | 5 | 4 | 4 |  | 4 | 4 | 3 | 5 | 4 | 4 | 2 | 1 | 3 | 2 | 2 | 2 |
|  | 3 |  |  | 5 | 4 | 5 | 4 | 5 | 5 |  | 5 | 4 | 5 | 4 | 5 | 5 | 2 | 2 | 1 | 2 | 3 | 2 |
|  | 4 |  |  | 3 | 3 | 3 | 2 | 4 | 3 |  | 3 | 3 | 3 | 2 | 4 | 3 | 2 | 1 | 3 | 2 | 2 | 2 |
|  | 5 |  |  | 5 | 4 | 5 | 4 | 5 | 5 |  | 5 | 4 | 5 | 4 | 5 | 5 | 2 | 2 | 1 | 2 | 3 | 2 |
|  | 6 |  |  | 4 | 3 | 4 | 4 | 5 | 4 |  | 4 | 4 | 3 | 5 | 4 | 4 | 2 | 1 | 3 | 2 | 2 | 2 |
|  | 7 |  |  | 4 | 5 | 3 | 4 | 4 | 4 |  | 4 | 5 | 3 | 4 | 4 | 4 | 2 | 2 | 1 | 2 | 3 | 2 |
|  | 8 |  |  | 4 | 4 | 3 | 5 | 4 | 4 |  | 4 | 4 | 3 | 5 | 4 | 4 | 2 | 1 | 3 | 2 | 2 | 2 |
|  | 9 |  |  | 2 | 1 | 2 | 2 | 3 | 2 |  | 2 | 2 | 1 | 2 | 3 | 2 | 2 | 2 | 1 | 2 | 3 | 2 |
|  | 10 |  |  | 4 | 3 | 4 | 4 | 5 | 4 |  | 4 | 3 | 4 | 4 | 5 | 4 | 2 | 1 | 3 | 2 | 2 | 2 |
|  | 11 |  |  | 3 | 3 | 3 | 2 | 4 | 3 |  | 3 | 3 | 3 | 2 | 4 | 3 | 2 | 2 | 1 | 2 | 3 | 2 |
|  | 12 |  |  | 4 | 5 | 3 | 4 | 4 | 4 |  | 4 | 5 | 3 | 4 | 4 | 4 | 2 | 2 | 1 | 2 | 3 | 2 |
|  | 13 |  |  | 4 | 4 | 3 | 5 | 4 | 4 |  | 4 | 4 | 3 | 5 | 4 | 4 | 2 | 1 | 3 | 2 | 2 | 2 |
|  | 14 |  |  | 5 | 4 | 5 | 4 | 5 | 5 |  | 5 | 4 | 5 | 4 | 5 | 5 | 2 | 2 | 1 | 2 | 3 | 2 |
|  | 15 |  |  | 3 | 3 | 3 | 2 | 4 | 3 |  | 3 | 3 | 3 | 2 | 4 | 3 | 2 | 1 | 3 | 2 | 2 | 2 |
|  | 16 |  |  | 4 | 5 | 3 | 4 | 4 | 4 |  | 4 | 5 | 3 | 4 | 4 | 4 | 2 | 2 | 1 | 2 | 3 | 2 |
|  | 17 |  |  | 4 | 4 | 3 | 5 | 4 | 4 |  | 4 | 4 | 3 | 5 | 4 | 4 | 2 | 1 | 3 | 2 | 2 | 2 |
|  | 18 |  |  | 3 | 2 | 4 | 3 | 3 | 3 |  | 3 | 2 | 4 | 3 | 3 | 3 | 2 | 2 | 1 | 2 | 3 | 2 |
|  | 19 |  |  | 3 | 3 | 3 | 2 | 4 | 3 |  | 3 | 3 | 3 | 2 | 4 | 3 | 1 | 1 | 1 | 1 | 2 | 1 |
|  | 20 |  |  | 4 | 5 | 3 | 4 | 4 | 4 |  | 4 | 5 | 3 | 4 | 4 | 4 | 2 | 1 | 3 | 2 | 2 | 2 |
|  | 21 |  |  | 4 | 4 | 3 | 5 | 4 | 4 |  | 4 | 5 | 3 | 4 | 4 | 4 | 2 | 2 | 1 | 2 | 3 | 2 |
|  | 22 |  |  | 4 | 4 | 3 | 5 | 4 | 4 |  | 4 | 4 | 3 | 5 | 4 | 4 | 2 | 1 | 3 | 2 | 2 | 2 |
|  | 23 |  |  | 3 | 3 | 3 | 2 | 4 | 3 |  | 3 | 3 | 3 | 2 | 4 | 3 | 2 | 2 | 1 | 2 | 3 | 2 |
|  | 24 |  |  | 4 | 3 | 4 | 4 | 5 | 4 |  | 4 | 3 | 4 | 4 | 5 | 4 | 2 | 1 | 3 | 2 | 2 | 2 |
|  | 25 |  |  | 3 | 3 | 3 | 2 | 4 | 3 |  | 3 | 3 | 3 | 2 | 4 | 3 | 2 | 2 | 1 | 2 | 3 | 2 |
|  | 26 |  |  | 4 | 5 | 3 | 4 | 4 | 4 |  | 4 | 5 | 3 | 4 | 4 | 4 | 2 | 1 | 3 | 2 | 2 | 2 |
|  | 27 |  |  | 4 | 4 | 3 | 5 | 4 | 4 |  | 4 | 4 | 3 | 5 | 4 | 4 | 2 | 2 | 1 | 2 | 3 | 2 |
|  | 28 |  |  | 3 | 3 | 3 | 2 | 4 | 3 |  | 3 | 3 | 3 | 2 | 4 | 3 | 2 | 1 | 3 | 2 | 2 | 2 |
|  | 29 |  |  | 5 | 4 | 5 | 4 | 5 | 5 |  | 5 | 4 | 5 | 4 | 5 | 5 | 2 | 2 | 1 | 2 | 3 | 2 |
|  | 30 |  |  | 4 | 3 | 4 | 4 | 5 | 4 |  | 4 | 3 | 4 | 4 | 5 | 4 | 2 | 1 | 3 | 2 | 2 | 2 |
|  | 31 |  |  | 5 | 4 | 5 | 4 | 5 | 5 |  | 5 | 4 | 5 | 4 | 5 | 5 | 2 | 2 | 1 | 2 | 3 | 2 |
|  | 32 |  |  | 4 | 3 | 4 | 4 | 5 | 4 |  | 4 | 3 | 4 | 4 | 5 | 4 | 2 | 1 | 3 | 2 | 2 | 2 |
|  | 33 |  |  | 3 | 2 | 4 | 3 | 3 | 3 |  | 3 | 2 | 4 | 3 | 3 | 3 | 2 | 2 | 1 | 2 | 3 | 2 |
|  | 34 |  |  | 3 | 3 | 3 | 2 | 4 | 3 |  | 3 | 3 | 3 | 2 | 4 | 3 | 2 | 1 | 3 | 2 | 2 | 2 |
|  | 35 |  |  | 5 | 4 | 5 | 4 | 5 | 5 |  | 5 | 4 | 5 | 4 | 5 | 5 | 2 | 2 | 1 | 2 | 3 | 2 |
|  | 36 |  |  | 4 | 5 | 3 | 4 | 4 | 4 |  | 4 | 5 | 3 | 4 | 4 | 4 | 2 | 1 | 3 | 2 | 2 | 2 |
|  | 37 |  |  | 4 | 4 | 3 | 5 | 4 | 4 |  | 4 | 4 | 3 | 5 | 4 | 4 | 2 | 1 | 3 | 2 | 2 | 2 |
|  | 38 |  |  | 4 | 5 | 3 | 4 | 4 | 4 |  | 4 | 5 | 3 | 4 | 4 | 4 | 2 | 2 | 1 | 2 | 3 | 2 |
|  | 39 |  |  | 4 | 5 | 3 | 4 | 4 | 4 |  | 4 | 4 | 3 | 5 | 4 | 4 | 2 | 1 | 3 | 2 | 2 | 2 |
|  | 40 |  |  | 4 | 4 | 3 | 5 | 4 | 4 |  | 4 | 5 | 3 | 4 | 4 | 4 | 2 | 2 | 1 | 2 | 3 | 2 |
|  | 41 |  |  | 4 | 5 | 3 | 4 | 4 | 4 |  | 4 | 4 | 3 | 5 | 4 | 4 | 2 | 1 | 3 | 2 | 2 | 2 |
|  | 42 |  |  | 4 | 4 | 3 | 5 | 4 | 4 |  | 4 | 4 | 3 | 5 | 4 | 4 | 2 | 2 | 1 | 2 | 3 | 2 |
|  | 43 |  |  | 3 | 3 | 3 | 2 | 4 | 3 |  | 3 | 3 | 3 | 2 | 4 | 3 | 2 | 1 | 3 | 2 | 2 | 2 |
|  | 44 |  |  | 4 | 3 | 4 | 4 | 5 | 4 |  | 4 | 3 | 4 | 4 | 5 | 4 | 2 | 1 | 3 | 2 | 2 | 2 |
|  | 45 |  |  | 3 | 3 | 3 | 2 | 4 | 3 |  | 3 | 3 | 3 | 2 | 4 | 3 | 2 | 2 | 1 | 2 | 3 | 2 |
|  | 46 |  |  | 2 | 2 | 1 | 2 | 3 | 2 |  | 2 | 2 | 1 | 2 | 3 | 2 | 2 | 1 | 3 | 2 | 2 | 2 |
|  | 47 |  |  | 1 | 1 | 1 | 1 | 2 | 1 |  | 1 | 1 | 1 | 1 | 2 | 1 | 2 | 2 | 1 | 2 | 3 | 2 |
|  | 48 |  |  | 4 | 3 | 4 | 4 | 5 | 4 |  | 4 | 3 | 4 | 4 | 5 | 4 | 2 | 1 | 3 | 2 | 2 | 2 |
|  | 49 |  |  | 3 | 2 | 4 | 3 | 3 | 3 |  | 3 | 2 | 4 | 3 | 3 | 3 | 2 | 2 | 1 | 2 | 3 | 2 |
|  | 50 |  |  | 3 | 3 | 3 | 2 | 4 | 3 |  | 3 | 3 | 3 | 2 | 4 | 3 | 2 | 1 | 3 | 2 | 2 | 2 |
|  | 1 |  | Failure to diagnose the patient quickly | 5 | 4 | 5 | 4 | 5 | 5 | Number Of Corrective Maintenance | 4 | 3 | 4 | 4 | 5 | 4 | 2 | 2 | 1 | 2 | 3 | 2 |
|  | 2 |  |  | 3 | 3 | 3 | 2 | 4 | 3 |  | 3 | 3 | 3 | 2 | 4 | 3 | 3 | 2 | 4 | 3 | 3 | 3 |
|  | 3 |  |  | 5 | 4 | 5 | 4 | 5 | 5 |  | 5 | 4 | 5 | 4 | 5 | 5 | 2 | 1 | 3 | 2 | 2 | 2 |
|  | 4 |  |  | 4 | 5 | 3 | 4 | 4 | 4 |  | 4 | 5 | 3 | 4 | 4 | 4 | 2 | 2 | 1 | 2 | 3 | 2 |
|  | 5 |  |  | 4 | 4 | 3 | 5 | 4 | 4 |  | 4 | 4 | 3 | 5 | 4 | 4 | 2 | 2 | 1 | 2 | 3 | 2 |
|  | 6 |  |  | 3 | 3 | 3 | 2 | 4 | 3 |  | 3 | 2 | 4 | 3 | 3 | 3 | 3 | 2 | 4 | 3 | 3 | 3 |
|  | 7 |  |  | 4 | 3 | 4 | 4 | 5 | 4 |  | 3 | 2 | 4 | 3 | 3 | 3 | 3 | 2 | 4 | 3 | 3 | 3 |
|  | 8 |  |  | 2 | 2 | 1 | 2 | 3 | 2 |  | 3 | 3 | 3 | 2 | 4 | 3 | 3 | 3 | 3 | 2 | 4 | 3 |
|  | 9 |  |  | 1 | 1 | 1 | 1 | 2 | 1 |  | 1 | 1 | 1 | 1 | 2 | 1 | 2 | 2 | 1 | 2 | 3 | 2 |
|  | 10 |  |  | 5 | 4 | 5 | 4 | 5 | 5 |  | 5 | 4 | 5 | 4 | 5 | 5 | 3 | 2 | 4 | 3 | 3 | 3 |
|  | 11 |  |  | 1 | 1 | 1 | 1 | 2 | 1 |  | 1 | 1 | 1 | 1 | 2 | 1 | 3 | 3 | 3 | 2 | 4 | 3 |
|  | 12 |  |  | 4 | 3 | 4 | 4 | 5 | 4 |  | 4 | 3 | 4 | 4 | 5 | 4 | 2 | 1 | 3 | 2 | 2 | 2 |
|  | 13 |  |  | 5 | 4 | 5 | 4 | 5 | 5 |  | 5 | 4 | 5 | 4 | 5 | 5 | 2 | 2 | 1 | 2 | 3 | 2 |
|  | 14 |  |  | 4 | 3 | 4 | 4 | 5 | 4 |  | 4 | 3 | 4 | 4 | 5 | 4 | 2 | 2 | 1 | 2 | 3 | 2 |
|  | 15 |  |  | 1 | 1 | 1 | 1 | 2 | 1 |  | 1 | 1 | 1 | 1 | 2 | 1 | 2 | 1 | 3 | 2 | 2 | 2 |
|  | 16 |  |  | 3 | 3 | 3 | 2 | 4 | 3 |  | 3 | 2 | 4 | 3 | 3 | 3 | 2 | 2 | 1 | 2 | 3 | 2 |
|  | 17 |  |  | 2 | 1 | 3 | 2 | 2 | 2 |  | 3 | 3 | 3 | 2 | 4 | 3 | 3 | 2 | 4 | 3 | 3 | 3 |
|  | 18 |  |  | 3 | 3 | 3 | 2 | 4 | 3 |  | 2 | 2 | 1 | 2 | 3 | 2 | 3 | 3 | 3 | 2 | 4 | 3 |
|  | 19 |  |  | 1 | 1 | 1 | 1 | 2 | 1 |  | 1 | 1 | 1 | 1 | 2 | 1 | 3 | 2 | 4 | 3 | 3 | 3 |
|  | 20 |  |  | 3 | 3 | 3 | 2 | 4 | 3 |  | 3 | 2 | 4 | 3 | 3 | 3 | 3 | 2 | 4 | 3 | 3 | 3 |
|  | 21 |  |  | 2 | 1 | 3 | 2 | 2 | 2 |  | 3 | 3 | 3 | 2 | 4 | 3 | 3 | 3 | 3 | 2 | 4 | 3 |
|  | 22 |  |  | 2 | 1 | 3 | 2 | 2 | 2 |  | 3 | 2 | 4 | 3 | 3 | 3 | 2 | 1 | 3 | 2 | 2 | 2 |
|  | 23 |  |  | 2 | 2 | 1 | 2 | 3 | 2 |  | 3 | 3 | 3 | 2 | 4 | 3 | 2 | 2 | 1 | 2 | 3 | 2 |
|  | 24 |  |  | 3 | 3 | 3 | 2 | 4 | 3 |  | 2 | 2 | 1 | 2 | 3 | 2 | 2 | 1 | 3 | 2 | 2 | 2 |
|  | 25 |  |  | 2 | 1 | 3 | 2 | 2 | 2 |  | 3 | 2 | 4 | 3 | 3 | 3 | 2 | 2 | 1 | 2 | 3 | 2 |
|  | 26 |  |  | 2 | 2 | 1 | 2 | 3 | 2 |  | 3 | 3 | 3 | 2 | 4 | 3 | 2 | 1 | 3 | 2 | 2 | 2 |
|  | 27 |  |  | 3 | 3 | 3 | 2 | 4 | 3 |  | 3 | 2 | 4 | 3 | 3 | 3 | 2 | 2 | 1 | 2 | 3 | 2 |
|  | 28 |  |  | 2 | 2 | 1 | 2 | 3 | 2 |  | 3 | 3 | 3 | 2 | 4 | 3 | 2 | 1 | 3 | 2 | 2 | 2 |
|  | 29 |  |  | 4 | 3 | 4 | 4 | 5 | 4 |  | 4 | 3 | 4 | 4 | 5 | 4 | 2 | 2 | 1 | 2 | 3 | 2 |
|  | 30 |  |  | 5 | 4 | 5 | 4 | 5 | 5 |  | 5 | 4 | 5 | 4 | 5 | 5 | 3 | 2 | 4 | 3 | 3 | 3 |
|  | 31 |  |  | 5 | 4 | 5 | 4 | 5 | 5 |  | 5 | 4 | 5 | 4 | 5 | 5 | 3 | 3 | 3 | 2 | 4 | 3 |
|  | 32 |  |  | 4 | 3 | 4 | 4 | 5 | 4 |  | 4 | 3 | 4 | 4 | 5 | 4 | 3 | 3 | 3 | 2 | 4 | 3 |
|  | 33 |  |  | 3 | 3 | 3 | 2 | 4 | 3 |  | 3 | 3 | 3 | 2 | 4 | 3 | 3 | 2 | 4 | 3 | 3 | 3 |
|  | 34 |  |  | 5 | 4 | 5 | 4 | 5 | 5 |  | 5 | 4 | 5 | 4 | 5 | 5 | 3 | 3 | 3 | 2 | 4 | 3 |
|  | 35 |  |  | 5 | 4 | 5 | 4 | 5 | 5 |  | 5 | 4 | 5 | 4 | 5 | 5 | 2 | 2 | 1 | 2 | 3 | 2 |
|  | 36 |  |  | 3 | 3 | 3 | 2 | 4 | 3 |  | 3 | 3 | 3 | 2 | 4 | 3 | 3 | 2 | 4 | 3 | 3 | 3 |
|  | 37 |  |  | 4 | 3 | 4 | 4 | 5 | 4 |  | 4 | 3 | 4 | 4 | 5 | 4 | 3 | 3 | 3 | 2 | 4 | 3 |
|  | 38 |  |  | 3 | 3 | 3 | 2 | 4 | 3 |  | 3 | 2 | 4 | 3 | 3 | 3 | 2 | 2 | 1 | 2 | 3 | 2 |
|  | 39 |  |  | 2 | 1 | 3 | 2 | 2 | 2 |  | 3 | 3 | 3 | 2 | 4 | 3 | 3 | 2 | 4 | 3 | 3 | 3 |
|  | 40 |  |  | 2 | 2 | 1 | 2 | 3 | 2 |  | 2 | 1 | 3 | 2 | 2 | 2 | 3 | 3 | 3 | 2 | 4 | 3 |
|  | 41 |  |  | 3 | 2 | 4 | 3 | 3 | 3 |  | 3 | 3 | 3 | 2 | 4 | 3 | 3 | 2 | 4 | 3 | 3 | 3 |
|  | 42 |  |  | 3 | 3 | 3 | 2 | 4 | 3 |  | 3 | 3 | 3 | 2 | 4 | 3 | 3 | 3 | 3 | 2 | 4 | 3 |
|  | 43 |  |  | 3 | 2 | 4 | 3 | 3 | 3 |  | 3 | 2 | 4 | 3 | 3 | 3 | 2 | 1 | 3 | 2 | 2 | 2 |
|  | 44 |  |  | 3 | 3 | 3 | 2 | 4 | 3 |  | 3 | 3 | 3 | 2 | 4 | 3 | 3 | 2 | 4 | 3 | 3 | 3 |
|  | 45 |  |  | 4 | 3 | 4 | 4 | 5 | 4 |  | 4 | 3 | 4 | 4 | 5 | 4 | 3 | 3 | 3 | 2 | 4 | 3 |
|  | 46 |  |  | 1 | 1 | 1 | 1 | 2 | 1 |  | 1 | 1 | 1 | 1 | 2 | 1 | 3 | 2 | 4 | 3 | 3 | 3 |
|  | 47 |  |  | 1 | 1 | 1 | 1 | 2 | 1 |  | 1 | 1 | 1 | 1 | 2 | 1 | 3 | 3 | 3 | 2 | 4 | 3 |
|  | 48 |  |  | 4 | 3 | 4 | 4 | 5 | 4 |  | 3 | 3 | 3 | 2 | 4 | 3 | 3 | 2 | 4 | 3 | 3 | 3 |
|  | 49 |  |  | 1 | 1 | 1 | 1 | 2 | 1 |  | 1 | 1 | 1 | 1 | 2 | 1 | 3 | 3 | 3 | 2 | 4 | 3 |
|  | 50 |  |  | 5 | 4 | 5 | 4 | 5 | 5 |  | 5 | 4 | 5 | 4 | 5 | 5 | 2 | 1 | 3 | 2 | 2 | 2 |
|  | 1 | The results  of the tests  are not accurate | Misdiagnosis of the  patient's condition | 3 | 3 | 3 | 2 | 4 | 3 | Response time for visit in Hour | 3 | 3 | 3 | 2 | 4 | 3 | 2 | 2 | 1 | 2 | 3 | 2 |
|  | 2 |  |  | 5 | 4 | 5 | 4 | 5 | 5 |  | 5 | 4 | 5 | 4 | 5 | 5 | 1 | 1 | 1 | 1 | 2 | 1 |
|  | 3 |  |  | 4 | 3 | 4 | 4 | 5 | 4 |  | 4 | 3 | 4 | 4 | 5 | 4 | 2 | 1 | 3 | 2 | 2 | 2 |
|  | 4 |  |  | 5 | 4 | 5 | 4 | 5 | 5 |  | 5 | 4 | 5 | 4 | 5 | 5 | 1 | 1 | 1 | 1 | 2 | 1 |
|  | 5 |  |  | 3 | 3 | 3 | 2 | 4 | 3 |  | 3 | 3 | 3 | 2 | 4 | 3 | 2 | 1 | 3 | 2 | 2 | 2 |
|  | 6 |  |  | 4 | 3 | 4 | 4 | 5 | 4 |  | 4 | 3 | 4 | 4 | 5 | 4 | 2 | 2 | 1 | 2 | 3 | 2 |
|  | 7 |  |  | 5 | 4 | 5 | 4 | 5 | 5 |  | 5 | 4 | 5 | 4 | 5 | 5 | 2 | 2 | 1 | 2 | 3 | 2 |
|  | 8 |  |  | 4 | 3 | 4 | 4 | 5 | 4 |  | 4 | 3 | 4 | 4 | 5 | 4 | 2 | 1 | 3 | 2 | 2 | 2 |
|  | 9 |  |  | 1 | 1 | 1 | 1 | 2 | 1 |  | 1 | 1 | 1 | 1 | 2 | 1 | 2 | 2 | 1 | 2 | 3 | 2 |
|  | 10 |  |  | 2 | 1 | 3 | 2 | 2 | 2 |  | 2 | 1 | 3 | 2 | 2 | 2 | 2 | 1 | 3 | 2 | 2 | 2 |
|  | 11 |  |  | 2 | 2 | 1 | 2 | 3 | 2 |  | 2 | 2 | 1 | 2 | 3 | 2 | 2 | 1 | 3 | 2 | 2 | 2 |
|  | 12 |  |  | 4 | 3 | 4 | 4 | 5 | 4 |  | 4 | 3 | 4 | 4 | 5 | 4 | 2 | 2 | 1 | 2 | 3 | 2 |
|  | 13 |  |  | 3 | 3 | 3 | 2 | 4 | 3 |  | 3 | 3 | 3 | 2 | 4 | 3 | 2 | 1 | 3 | 2 | 2 | 2 |
|  | 14 |  |  | 2 | 1 | 3 | 2 | 2 | 2 |  | 2 | 1 | 3 | 2 | 2 | 2 | 2 | 2 | 1 | 2 | 3 | 2 |
|  | 15 |  |  | 2 | 2 | 1 | 2 | 3 | 2 |  | 2 | 2 | 1 | 2 | 3 | 2 | 2 | 1 | 3 | 2 | 2 | 2 |
|  | 16 |  |  | 2 | 2 | 1 | 2 | 3 | 2 |  | 2 | 2 | 1 | 2 | 3 | 2 | 2 | 2 | 1 | 2 | 3 | 2 |
|  | 17 |  |  | 5 | 4 | 5 | 4 | 5 | 5 |  | 5 | 4 | 5 | 4 | 5 | 5 | 2 | 1 | 3 | 2 | 2 | 2 |
|  | 18 |  |  | 4 | 5 | 3 | 4 | 4 | 4 |  | 4 | 5 | 3 | 4 | 4 | 4 | 2 | 2 | 1 | 2 | 3 | 2 |
|  | 19 |  |  | 4 | 4 | 3 | 5 | 4 | 4 |  | 4 | 4 | 3 | 5 | 4 | 4 | 2 | 1 | 3 | 2 | 2 | 2 |
|  | 20 |  |  | 5 | 4 | 5 | 4 | 5 | 5 |  | 5 | 4 | 5 | 4 | 5 | 5 | 2 | 2 | 1 | 2 | 3 | 2 |
|  | 21 |  |  | 4 | 5 | 3 | 4 | 4 | 4 |  | 4 | 5 | 3 | 4 | 4 | 4 | 2 | 1 | 3 | 2 | 2 | 2 |
|  | 22 |  |  | 4 | 4 | 3 | 5 | 4 | 4 |  | 4 | 4 | 3 | 5 | 4 | 4 | 2 | 2 | 1 | 2 | 3 | 2 |
|  | 23 |  |  | 5 | 4 | 5 | 4 | 5 | 5 |  | 5 | 4 | 5 | 4 | 5 | 5 | 2 | 1 | 3 | 2 | 2 | 2 |
|  | 24 |  |  | 3 | 3 | 3 | 2 | 4 | 3 |  | 3 | 3 | 3 | 2 | 4 | 3 | 2 | 2 | 1 | 2 | 3 | 2 |
|  | 25 |  |  | 4 | 3 | 4 | 4 | 5 | 4 |  | 4 | 3 | 4 | 4 | 5 | 4 | 2 | 1 | 3 | 2 | 2 | 2 |
|  | 26 |  |  | 5 | 4 | 5 | 4 | 5 | 5 |  | 5 | 4 | 5 | 4 | 5 | 5 | 2 | 2 | 1 | 2 | 3 | 2 |
|  | 27 |  |  | 4 | 5 | 3 | 4 | 4 | 4 |  | 4 | 5 | 3 | 4 | 4 | 4 | 2 | 1 | 3 | 2 | 2 | 2 |
|  | 28 |  |  | 4 | 4 | 3 | 5 | 4 | 4 |  | 4 | 4 | 3 | 5 | 4 | 4 | 2 | 2 | 1 | 2 | 3 | 2 |
|  | 29 |  |  | 4 | 5 | 3 | 4 | 4 | 4 |  | 4 | 5 | 3 | 4 | 4 | 4 | 2 | 2 | 1 | 2 | 3 | 2 |
|  | 30 |  |  | 3 | 3 | 3 | 2 | 4 | 3 |  | 3 | 3 | 3 | 2 | 4 | 3 | 2 | 1 | 3 | 2 | 2 | 2 |
|  | 31 |  |  | 4 | 5 | 3 | 4 | 4 | 4 |  | 4 | 5 | 3 | 4 | 4 | 4 | 2 | 2 | 1 | 2 | 3 | 2 |
|  | 32 |  |  | 4 | 4 | 3 | 5 | 4 | 4 |  | 4 | 4 | 3 | 5 | 4 | 4 | 2 | 1 | 3 | 2 | 2 | 2 |
|  | 33 |  |  | 3 | 3 | 3 | 2 | 4 | 3 |  | 3 | 3 | 3 | 2 | 4 | 3 | 2 | 2 | 1 | 2 | 3 | 2 |
|  | 34 |  |  | 5 | 4 | 5 | 4 | 5 | 5 |  | 5 | 4 | 5 | 4 | 5 | 5 | 2 | 1 | 3 | 2 | 2 | 2 |
|  | 35 |  |  | 5 | 5 | 4 | 4 | 5 | 5 |  | 5 | 5 | 4 | 4 | 5 | 5 | 2 | 2 | 1 | 2 | 3 | 2 |
|  | 36 |  |  | 5 | 4 | 5 | 4 | 5 | 5 |  | 5 | 4 | 5 | 4 | 5 | 5 | 2 | 2 | 1 | 2 | 3 | 2 |
|  | 37 |  |  | 4 | 5 | 3 | 4 | 4 | 4 |  | 5 | 5 | 4 | 4 | 5 | 5 | 2 | 1 | 3 | 2 | 2 | 2 |
|  | 38 |  |  | 4 | 4 | 3 | 5 | 4 | 4 |  | 5 | 4 | 5 | 4 | 5 | 5 | 2 | 2 | 1 | 2 | 3 | 2 |
|  | 39 |  |  | 5 | 4 | 5 | 4 | 5 | 5 |  | 5 | 5 | 4 | 4 | 5 | 5 | 2 | 1 | 3 | 2 | 2 | 2 |
|  | 40 |  |  | 4 | 4 | 3 | 5 | 4 | 4 |  | 4 | 3 | 4 | 4 | 5 | 4 | 2 | 2 | 1 | 2 | 3 | 2 |
|  | 41 |  |  | 5 | 4 | 5 | 4 | 5 | 5 |  | 5 | 4 | 5 | 4 | 5 | 5 | 2 | 1 | 3 | 2 | 2 | 2 |
|  | 42 |  |  | 5 | 5 | 4 | 4 | 5 | 5 |  | 5 | 5 | 4 | 4 | 5 | 5 | 2 | 2 | 1 | 2 | 3 | 2 |
|  | 43 |  |  | 4 | 5 | 3 | 4 | 4 | 4 |  | 5 | 4 | 5 | 4 | 5 | 5 | 2 | 1 | 3 | 2 | 2 | 2 |
|  | 44 |  |  | 4 | 4 | 3 | 5 | 4 | 4 |  | 5 | 5 | 4 | 4 | 5 | 5 | 2 | 1 | 3 | 2 | 2 | 2 |
|  | 45 |  |  | 5 | 4 | 5 | 4 | 5 | 5 |  | 5 | 4 | 5 | 4 | 5 | 5 | 2 | 2 | 1 | 2 | 3 | 2 |
|  | 46 |  |  | 5 | 5 | 4 | 4 | 5 | 5 |  | 5 | 5 | 4 | 4 | 5 | 5 | 2 | 1 | 3 | 2 | 2 | 2 |
|  | 47 |  |  | 3 | 3 | 3 | 2 | 4 | 3 |  | 3 | 3 | 3 | 2 | 4 | 3 | 2 | 2 | 1 | 2 | 3 | 2 |
|  | 48 |  |  | 5 | 4 | 5 | 4 | 5 | 5 |  | 5 | 4 | 5 | 4 | 5 | 5 | 2 | 1 | 3 | 2 | 2 | 2 |
|  | 49 |  |  | 5 | 4 | 5 | 4 | 5 | 5 |  | 5 | 5 | 4 | 4 | 5 | 5 | 2 | 2 | 1 | 2 | 3 | 2 |
|  | 50 |  |  | 5 | 5 | 4 | 4 | 5 | 5 |  | 5 | 4 | 5 | 4 | 5 | 5 | 2 | 2 | 1 | 2 | 3 | 2 |

Survey for centrifuge devices.

| Experts ratings for S, P, and D for centrifuge devices | | | | | | | | | | | | | | | | | | | | | | |
| --- | --- | --- | --- | --- | --- | --- | --- | --- | --- | --- | --- | --- | --- | --- | --- | --- | --- | --- | --- | --- | --- | --- |
|  |  |  |  | Experts Severity | | | | |  | Experts Probability | | | | | Experts Detection | | | | |  |  |  |
| Function | Device no. | Failure mode | Effects of Failure | E1 | E2 | E3 | E4 | E5 | Average | Causes of Failure | E1 | E2 | E3 | E4 | E5 | Average | E1 | E2 | E3 | E4 | E5 | Average |
| Separate fluids, gases,or liquid samples based on density | 1 | Some devices are not working | pressure on working devices | 1 | 1 | 2 | 1 | 2 | 1 | number of complaints | 5 | 4 | 5 | 4 | 5 | 5 | 2 | 1 | 3 | 2 | 2 | 2 |
|  | 2 |  |  | 1 | 2 | 1 | 1 | 2 | 1 |  | 3 | 2 | 3 | 4 | 3 | 3 | 2 | 3 | 2 | 1 | 2 | 2 |
|  | 3 |  |  | 1 | 1 | 2 | 1 | 2 | 1 |  | 5 | 4 | 5 | 4 | 5 | 5 | 2 | 2 | 1 | 2 | 3 | 2 |
|  | 4 |  |  | 1 | 2 | 1 | 1 | 2 | 1 |  | 4 | 3 | 4 | 5 | 4 | 4 | 2 | 2 | 1 | 2 | 3 | 2 |
|  | 5 |  |  | 2 | 2 | 1 | 2 | 3 | 2 |  | 5 | 4 | 5 | 4 | 5 | 5 | 1 | 2 | 1 | 1 | 2 | 1 |
|  | 6 |  |  | 2 | 1 | 2 | 2 | 3 | 2 |  | 4 | 3 | 4 | 5 | 4 | 4 | 1 | 1 | 1 | 1 | 2 | 1 |
|  | 7 |  |  | 3 | 2 | 3 | 4 | 3 | 3 |  | 3 | 2 | 3 | 4 | 3 | 3 | 1 | 2 | 1 | 1 | 2 | 1 |
|  | 8 |  |  | 1 | 1 | 2 | 1 | 2 | 1 |  | 3 | 2 | 3 | 3 | 3 | 3 | 1 | 1 | 1 | 1 | 2 | 1 |
|  | 9 |  |  | 3 | 2 | 3 | 4 | 3 | 3 |  | 1 | 1 | 2 | 1 | 2 | 1 | 1 | 1 | 1 | 1 | 2 | 1 |
|  | 10 |  |  | 1 | 1 | 2 | 1 | 2 | 1 |  | 5 | 4 | 5 | 4 | 5 | 5 | 2 | 3 | 2 | 1 | 2 | 2 |
|  | 11 |  |  | 1 | 2 | 1 | 1 | 2 | 1 |  | 1 | 1 | 2 | 1 | 2 | 1 | 1 | 2 | 1 | 1 | 2 | 1 |
|  | 12 |  |  | 1 | 1 | 2 | 1 | 2 | 1 |  | 4 | 3 | 4 | 5 | 4 | 4 | 2 | 1 | 3 | 2 | 2 | 2 |
|  | 13 |  |  | 1 | 2 | 1 | 1 | 2 | 1 |  | 5 | 4 | 5 | 5 | 4 | 5 | 2 | 3 | 2 | 1 | 2 | 2 |
|  | 14 |  |  | 1 | 1 | 2 | 1 | 2 | 1 |  | 5 | 4 | 5 | 4 | 5 | 5 | 2 | 2 | 1 | 2 | 3 | 2 |
|  | 15 |  |  | 1 | 2 | 1 | 1 | 2 | 1 |  | 1 | 1 | 2 | 1 | 2 | 1 | 2 | 2 | 1 | 2 | 3 | 2 |
|  | 16 |  |  | 1 | 1 | 2 | 1 | 2 | 1 |  | 3 | 2 | 3 | 4 | 3 | 3 | 2 | 3 | 2 | 1 | 2 | 2 |
|  | 17 |  |  | 3 | 2 | 3 | 4 | 3 | 3 |  | 3 | 2 | 3 | 3 | 3 | 3 | 1 | 2 | 1 | 1 | 2 | 1 |
|  | 18 |  |  | 2 | 1 | 2 | 2 | 3 | 2 |  | 2 | 1 | 2 | 2 | 3 | 2 | 1 | 1 | 1 | 1 | 2 | 1 |
|  | 19 |  |  | 1 | 1 | 2 | 1 | 2 | 1 |  | 1 | 2 | 1 | 1 | 2 | 1 | 1 | 1 | 1 | 1 | 2 | 1 |
|  | 20 |  |  | 1 | 2 | 1 | 1 | 2 | 1 |  | 3 | 2 | 3 | 4 | 3 | 3 | 2 | 1 | 3 | 2 | 2 | 2 |
|  | 21 |  |  | 1 | 1 | 2 | 1 | 2 | 1 |  | 3 | 2 | 3 | 3 | 3 | 3 | 2 | 3 | 2 | 1 | 2 | 2 |
|  | 22 |  |  | 1 | 2 | 1 | 1 | 2 | 1 |  | 3 | 2 | 3 | 4 | 3 | 3 | 2 | 2 | 1 | 2 | 3 | 2 |
|  | 23 |  |  | 1 | 1 | 2 | 1 | 2 | 1 |  | 3 | 2 | 3 | 3 | 3 | 3 | 2 | 1 | 3 | 2 | 2 | 2 |
|  | 24 |  |  | 1 | 2 | 1 | 1 | 2 | 1 |  | 2 | 1 | 2 | 2 | 3 | 2 | 2 | 3 | 2 | 1 | 2 | 2 |
|  | 25 |  |  | 1 | 1 | 2 | 1 | 2 | 1 |  | 3 | 2 | 3 | 4 | 3 | 3 | 2 | 2 | 1 | 2 | 3 | 2 |
|  | 26 |  |  | 1 | 1 | 2 | 1 | 2 | 1 |  | 3 | 2 | 3 | 4 | 3 | 3 | 2 | 1 | 3 | 2 | 2 | 2 |
|  | 27 |  |  | 1 | 2 | 1 | 1 | 2 | 1 |  | 3 | 2 | 3 | 4 | 3 | 3 | 2 | 3 | 2 | 1 | 2 | 2 |
|  | 28 |  |  | 1 | 1 | 2 | 1 | 2 | 1 |  | 3 | 2 | 3 | 4 | 3 | 3 | 2 | 2 | 1 | 2 | 3 | 2 |
|  | 29 |  |  | 1 | 2 | 1 | 1 | 2 | 1 |  | 4 | 3 | 4 | 5 | 4 | 4 | 2 | 1 | 3 | 2 | 2 | 2 |
|  | 30 |  |  | 1 | 1 | 2 | 1 | 2 | 1 |  | 5 | 4 | 5 | 4 | 5 | 5 | 2 | 3 | 2 | 1 | 2 | 2 |
|  | 31 |  |  | 1 | 2 | 1 | 1 | 2 | 1 |  | 5 | 4 | 5 | 4 | 5 | 5 | 2 | 2 | 1 | 2 | 3 | 2 |
|  | 32 |  |  | 1 | 1 | 2 | 1 | 2 | 1 |  | 4 | 3 | 4 | 5 | 4 | 4 | 2 | 1 | 3 | 2 | 2 | 2 |
|  | 33 |  |  | 1 | 2 | 1 | 1 | 2 | 1 |  | 3 | 2 | 3 | 4 | 3 | 3 | 2 | 3 | 2 | 1 | 2 | 2 |
|  | 34 |  |  | 1 | 1 | 2 | 1 | 2 | 1 |  | 5 | 4 | 5 | 4 | 5 | 5 | 2 | 2 | 1 | 2 | 3 | 2 |
|  | 35 |  |  | 1 | 2 | 1 | 1 | 2 | 1 |  | 5 | 4 | 5 | 4 | 5 | 5 | 2 | 1 | 3 | 2 | 2 | 2 |
|  | 36 |  |  | 1 | 1 | 2 | 1 | 2 | 1 |  | 3 | 2 | 3 | 4 | 3 | 3 | 2 | 3 | 2 | 1 | 2 | 2 |
|  | 37 |  |  | 1 | 2 | 1 | 1 | 2 | 1 |  | 4 | 3 | 4 | 5 | 4 | 4 | 2 | 2 | 1 | 2 | 3 | 2 |
|  | 38 |  |  | 1 | 1 | 2 | 1 | 2 | 1 |  | 3 | 2 | 3 | 4 | 3 | 3 | 2 | 1 | 3 | 2 | 2 | 2 |
|  | 39 |  |  | 1 | 2 | 1 | 1 | 2 | 1 |  | 3 | 2 | 3 | 3 | 3 | 3 | 2 | 3 | 2 | 1 | 2 | 2 |
|  | 40 |  |  | 1 | 1 | 2 | 1 | 2 | 1 |  | 2 | 1 | 2 | 2 | 3 | 2 | 2 | 2 | 1 | 2 | 3 | 2 |
|  | 41 |  |  | 1 | 2 | 1 | 1 | 2 | 1 |  | 3 | 2 | 3 | 4 | 3 | 3 | 2 | 1 | 3 | 2 | 2 | 2 |
|  | 42 |  |  | 1 | 1 | 2 | 1 | 2 | 1 |  | 3 | 2 | 3 | 3 | 3 | 3 | 2 | 3 | 2 | 1 | 2 | 2 |
|  | 43 |  |  | 1 | 2 | 1 | 1 | 2 | 1 |  | 3 | 2 | 3 | 4 | 3 | 3 | 2 | 2 | 1 | 2 | 3 | 2 |
|  | 44 |  |  | 1 | 1 | 2 | 1 | 2 | 1 |  | 3 | 2 | 3 | 3 | 3 | 3 | 2 | 1 | 3 | 2 | 2 | 2 |
|  | 45 |  |  | 1 | 2 | 1 | 1 | 2 | 1 |  | 5 | 4 | 5 | 4 | 5 | 5 | 2 | 3 | 2 | 1 | 2 | 2 |
|  | 46 |  |  | 1 | 1 | 2 | 1 | 2 | 1 |  | 1 | 1 | 2 | 1 | 2 | 1 | 2 | 1 | 3 | 2 | 2 | 2 |
|  | 47 |  |  | 1 | 2 | 1 | 1 | 2 | 1 |  | 1 | 2 | 1 | 1 | 2 | 1 | 2 | 3 | 2 | 1 | 2 | 2 |
|  | 48 |  |  | 1 | 1 | 2 | 1 | 2 | 1 |  | 4 | 3 | 4 | 5 | 4 | 4 | 2 | 2 | 1 | 2 | 3 | 2 |
|  | 49 |  |  | 2 | 1 | 2 | 2 | 3 | 2 |  | 1 | 1 | 2 | 1 | 2 | 1 | 1 | 2 | 1 | 1 | 2 | 1 |
|  | 50 |  |  | 1 | 1 | 2 | 1 | 2 | 1 |  | 5 | 4 | 5 | 4 | 5 | 5 | 1 | 2 | 1 | 1 | 2 | 1 |
|  | 1 | Delayed test result | Patient dissatisfaction | 2 | 1 | 2 | 2 | 3 | 2 | Number Of Preventive Maintenance | 3 | 2 | 3 | 4 | 3 | 3 | 1 | 1 | 1 | 1 | 2 | 1 |
|  | 2 |  |  | 3 | 2 | 3 | 4 | 3 | 3 |  | 3 | 2 | 3 | 3 | 3 | 3 | 1 | 2 | 1 | 1 | 2 | 1 |
|  | 3 |  |  | 1 | 1 | 2 | 1 | 2 | 1 |  | 3 | 2 | 3 | 4 | 3 | 3 | 1 | 2 | 1 | 1 | 2 | 1 |
|  | 4 |  |  | 4 | 3 | 4 | 5 | 4 | 4 |  | 3 | 2 | 3 | 3 | 3 | 3 | 1 | 1 | 1 | 1 | 2 | 1 |
|  | 5 |  |  | 5 | 4 | 5 | 4 | 5 | 5 |  | 3 | 2 | 3 | 4 | 3 | 3 | 1 | 2 | 1 | 1 | 2 | 1 |
|  | 6 |  |  | 3 | 2 | 3 | 4 | 3 | 3 |  | 3 | 2 | 3 | 3 | 3 | 3 | 1 | 2 | 1 | 1 | 2 | 1 |
|  | 7 |  |  | 2 | 1 | 2 | 2 | 3 | 2 |  | 3 | 2 | 3 | 4 | 3 | 3 | 1 | 1 | 1 | 1 | 2 | 1 |
|  | 8 |  |  | 3 | 2 | 3 | 4 | 3 | 3 |  | 3 | 2 | 3 | 3 | 3 | 3 | 1 | 2 | 1 | 1 | 2 | 1 |
|  | 9 |  |  | 1 | 1 | 2 | 1 | 2 | 1 |  | 3 | 2 | 3 | 4 | 3 | 3 | 1 | 2 | 1 | 1 | 2 | 1 |
|  | 10 |  |  | 4 | 3 | 4 | 5 | 4 | 4 |  | 3 | 2 | 3 | 3 | 3 | 3 | 1 | 1 | 1 | 1 | 2 | 1 |
|  | 11 |  |  | 5 | 4 | 5 | 4 | 5 | 5 |  | 3 | 2 | 3 | 4 | 3 | 3 | 1 | 2 | 1 | 1 | 2 | 1 |
|  | 12 |  |  | 3 | 2 | 3 | 4 | 3 | 3 |  | 3 | 2 | 3 | 3 | 3 | 3 | 1 | 2 | 1 | 1 | 2 | 1 |
|  | 13 |  |  | 3 | 2 | 3 | 3 | 3 | 3 |  | 3 | 2 | 3 | 4 | 3 | 3 | 1 | 1 | 1 | 1 | 2 | 1 |
|  | 14 |  |  | 4 | 3 | 4 | 5 | 4 | 4 |  | 3 | 2 | 3 | 3 | 3 | 3 | 1 | 2 | 1 | 1 | 2 | 1 |
|  | 15 |  |  | 3 | 2 | 3 | 4 | 3 | 3 |  | 3 | 2 | 3 | 4 | 3 | 3 | 1 | 2 | 1 | 1 | 2 | 1 |
|  | 16 |  |  | 2 | 1 | 2 | 2 | 3 | 2 |  | 3 | 2 | 3 | 3 | 3 | 3 | 1 | 1 | 1 | 1 | 2 | 1 |
|  | 17 |  |  | 3 | 2 | 3 | 4 | 3 | 3 |  | 3 | 2 | 3 | 4 | 3 | 3 | 1 | 2 | 1 | 1 | 2 | 1 |
|  | 18 |  |  | 1 | 1 | 2 | 1 | 2 | 1 |  | 3 | 2 | 3 | 3 | 3 | 3 | 1 | 1 | 1 | 1 | 2 | 1 |
|  | 19 |  |  | 3 | 2 | 3 | 4 | 3 | 3 |  | 3 | 2 | 3 | 4 | 3 | 3 | 1 | 2 | 1 | 1 | 2 | 1 |
|  | 20 |  |  | 4 | 3 | 4 | 5 | 4 | 4 |  | 3 | 2 | 3 | 3 | 3 | 3 | 1 | 2 | 1 | 1 | 2 | 1 |
|  | 21 |  |  | 2 | 1 | 2 | 2 | 3 | 2 |  | 3 | 2 | 3 | 4 | 3 | 3 | 1 | 1 | 1 | 1 | 2 | 1 |
|  | 22 |  |  | 3 | 2 | 3 | 4 | 3 | 3 |  | 3 | 2 | 3 | 3 | 3 | 3 | 1 | 2 | 1 | 1 | 2 | 1 |
|  | 23 |  |  | 2 | 1 | 2 | 2 | 3 | 2 |  | 3 | 2 | 3 | 4 | 3 | 3 | 1 | 2 | 1 | 1 | 2 | 1 |
|  | 24 |  |  | 3 | 2 | 3 | 4 | 3 | 3 |  | 3 | 2 | 3 | 3 | 3 | 3 | 1 | 1 | 1 | 1 | 2 | 1 |
|  | 25 |  |  | 3 | 2 | 3 | 3 | 3 | 3 |  | 2 | 1 | 2 | 2 | 3 | 2 | 1 | 2 | 1 | 1 | 2 | 1 |
|  | 26 |  |  | 4 | 3 | 4 | 5 | 4 | 4 |  | 2 | 1 | 2 | 2 | 3 | 2 | 1 | 2 | 1 | 1 | 2 | 1 |
|  | 27 |  |  | 3 | 2 | 3 | 4 | 3 | 3 |  | 3 | 2 | 3 | 4 | 3 | 3 | 1 | 1 | 1 | 1 | 2 | 1 |
|  | 28 |  |  | 1 | 1 | 2 | 1 | 2 | 1 |  | 2 | 2 | 1 | 2 | 3 | 2 | 1 | 2 | 1 | 1 | 2 | 1 |
|  | 29 |  |  | 2 | 1 | 2 | 2 | 3 | 2 |  | 2 | 1 | 2 | 2 | 3 | 2 | 1 | 2 | 1 | 1 | 2 | 1 |
|  | 30 |  |  | 3 | 2 | 3 | 4 | 3 | 3 |  | 2 | 2 | 1 | 2 | 3 | 2 | 1 | 1 | 1 | 1 | 2 | 1 |
|  | 31 |  |  | 4 | 3 | 4 | 5 | 4 | 4 |  | 2 | 1 | 2 | 2 | 3 | 2 | 1 | 2 | 1 | 1 | 2 | 1 |
|  | 32 |  |  | 5 | 4 | 5 | 4 | 5 | 5 |  | 2 | 2 | 1 | 2 | 3 | 2 | 1 | 2 | 1 | 1 | 2 | 1 |
|  | 33 |  |  | 2 | 2 | 1 | 2 | 3 | 2 |  | 2 | 1 | 2 | 2 | 3 | 2 | 1 | 1 | 1 | 1 | 2 | 1 |
|  | 34 |  |  | 2 | 1 | 2 | 2 | 3 | 2 |  | 2 | 2 | 1 | 2 | 3 | 2 | 1 | 2 | 1 | 1 | 2 | 1 |
|  | 35 |  |  | 2 | 2 | 1 | 2 | 3 | 2 |  | 2 | 1 | 2 | 2 | 3 | 2 | 1 | 2 | 1 | 1 | 2 | 1 |
|  | 36 |  |  | 2 | 2 | 1 | 2 | 3 | 2 |  | 2 | 2 | 1 | 2 | 3 | 2 | 1 | 1 | 1 | 1 | 2 | 1 |
|  | 37 |  |  | 2 | 1 | 2 | 2 | 3 | 2 |  | 2 | 1 | 2 | 2 | 3 | 2 | 1 | 2 | 1 | 1 | 2 | 1 |
|  | 38 |  |  | 3 | 2 | 3 | 4 | 3 | 3 |  | 3 | 2 | 3 | 4 | 3 | 3 | 1 | 2 | 1 | 1 | 2 | 1 |
|  | 39 |  |  | 3 | 2 | 3 | 4 | 3 | 3 |  | 3 | 2 | 3 | 3 | 3 | 3 | 1 | 1 | 1 | 1 | 2 | 1 |
|  | 40 |  |  | 3 | 2 | 3 | 3 | 3 | 3 |  | 3 | 2 | 3 | 4 | 3 | 3 | 1 | 2 | 1 | 1 | 2 | 1 |
|  | 41 |  |  | 3 | 2 | 3 | 4 | 3 | 3 |  | 3 | 2 | 3 | 4 | 3 | 3 | 1 | 2 | 1 | 1 | 2 | 1 |
|  | 42 |  |  | 3 | 2 | 3 | 3 | 3 | 3 |  | 3 | 2 | 3 | 3 | 3 | 3 | 1 | 1 | 1 | 1 | 2 | 1 |
|  | 43 |  |  | 2 | 1 | 2 | 2 | 3 | 2 |  | 3 | 2 | 3 | 4 | 3 | 3 | 1 | 2 | 1 | 1 | 2 | 1 |
|  | 44 |  |  | 3 | 2 | 3 | 4 | 3 | 3 |  | 3 | 2 | 3 | 4 | 3 | 3 | 1 | 2 | 1 | 1 | 2 | 1 |
|  | 45 |  |  | 3 | 2 | 3 | 3 | 3 | 3 |  | 3 | 2 | 3 | 3 | 3 | 3 | 1 | 1 | 1 | 1 | 2 | 1 |
|  | 46 |  |  | 5 | 4 | 5 | 4 | 5 | 5 |  | 2 | 2 | 1 | 2 | 3 | 2 | 1 | 2 | 1 | 1 | 2 | 1 |
|  | 47 |  |  | 2 | 1 | 2 | 2 | 3 | 2 |  | 2 | 1 | 2 | 2 | 3 | 2 | 1 | 2 | 1 | 1 | 2 | 1 |
|  | 48 |  |  | 5 | 4 | 5 | 4 | 5 | 5 |  | 3 | 2 | 3 | 4 | 3 | 3 | 1 | 1 | 1 | 1 | 2 | 1 |
|  | 49 |  |  | 4 | 3 | 4 | 5 | 4 | 4 |  | 3 | 2 | 3 | 4 | 3 | 3 | 1 | 2 | 1 | 1 | 2 | 1 |
|  | 50 |  |  | 2 | 1 | 2 | 2 | 3 | 2 |  | 3 | 2 | 3 | 3 | 3 | 3 | 1 | 2 | 1 | 1 | 2 | 1 |
|  | 1 |  | Failure to diagnose the patient quickly | 1 | 1 | 2 | 1 | 2 | 1 | Number Of Corrective Maintenance | 1 | 1 | 2 | 1 | 2 | 1 | 1 | 1 | 1 | 1 | 2 | 1 |
|  | 2 |  |  | 1 | 2 | 1 | 1 | 2 | 1 |  | 1 | 2 | 1 | 1 | 2 | 1 | 1 | 2 | 1 | 1 | 2 | 1 |
|  | 3 |  |  | 1 | 1 | 2 | 1 | 2 | 1 |  | 1 | 1 | 2 | 1 | 2 | 1 | 1 | 2 | 1 | 1 | 2 | 1 |
|  | 4 |  |  | 1 | 1 | 2 | 1 | 2 | 1 |  | 1 | 1 | 2 | 1 | 2 | 1 | 1 | 1 | 1 | 1 | 2 | 1 |
|  | 5 |  |  | 1 | 2 | 1 | 1 | 2 | 1 |  | 1 | 2 | 1 | 1 | 2 | 1 | 2 | 2 | 1 | 2 | 3 | 2 |
|  | 6 |  |  | 2 | 1 | 2 | 2 | 3 | 2 |  | 2 | 1 | 2 | 2 | 3 | 2 | 2 | 1 | 3 | 2 | 2 | 2 |
|  | 7 |  |  | 3 | 2 | 3 | 4 | 3 | 3 |  | 3 | 2 | 3 | 4 | 3 | 3 | 2 | 2 | 1 | 2 | 3 | 2 |
|  | 8 |  |  | 1 | 1 | 2 | 1 | 2 | 1 |  | 2 | 1 | 2 | 2 | 3 | 2 | 2 | 1 | 3 | 2 | 2 | 2 |
|  | 9 |  |  | 3 | 2 | 3 | 4 | 3 | 3 |  | 3 | 2 | 3 | 4 | 3 | 3 | 2 | 3 | 2 | 1 | 2 | 2 |
|  | 10 |  |  | 1 | 1 | 2 | 1 | 2 | 1 |  | 1 | 1 | 2 | 1 | 2 | 1 | 1 | 2 | 1 | 1 | 2 | 1 |
|  | 11 |  |  | 1 | 2 | 1 | 1 | 2 | 1 |  | 2 | 1 | 2 | 2 | 3 | 2 | 2 | 2 | 1 | 2 | 3 | 2 |
|  | 12 |  |  | 1 | 1 | 2 | 1 | 2 | 1 |  | 1 | 1 | 2 | 1 | 2 | 1 | 1 | 2 | 1 | 1 | 2 | 1 |
|  | 13 |  |  | 1 | 2 | 1 | 1 | 2 | 1 |  | 1 | 2 | 1 | 1 | 2 | 1 | 1 | 2 | 1 | 1 | 2 | 1 |
|  | 14 |  |  | 1 | 1 | 2 | 1 | 2 | 1 |  | 1 | 1 | 2 | 1 | 2 | 1 | 1 | 1 | 1 | 1 | 2 | 1 |
|  | 15 |  |  | 1 | 1 | 2 | 1 | 2 | 1 |  | 1 | 1 | 2 | 1 | 2 | 1 | 1 | 2 | 1 | 1 | 2 | 1 |
|  | 16 |  |  | 1 | 2 | 1 | 1 | 2 | 1 |  | 1 | 2 | 1 | 1 | 2 | 1 | 1 | 1 | 1 | 1 | 2 | 1 |
|  | 17 |  |  | 3 | 2 | 3 | 4 | 3 | 3 |  | 3 | 2 | 3 | 4 | 3 | 3 | 2 | 2 | 1 | 2 | 3 | 2 |
|  | 18 |  |  | 2 | 1 | 2 | 2 | 3 | 2 |  | 2 | 2 | 1 | 2 | 3 | 2 | 2 | 1 | 3 | 2 | 2 | 2 |
|  | 19 |  |  | 1 | 1 | 2 | 1 | 2 | 1 |  | 2 | 1 | 2 | 2 | 3 | 2 | 2 | 2 | 1 | 2 | 3 | 2 |
|  | 20 |  |  | 1 | 2 | 1 | 1 | 2 | 1 |  | 1 | 1 | 2 | 1 | 2 | 1 | 1 | 2 | 1 | 1 | 2 | 1 |
|  | 21 |  |  | 1 | 1 | 2 | 1 | 2 | 1 |  | 1 | 2 | 1 | 1 | 2 | 1 | 1 | 2 | 1 | 1 | 2 | 1 |
|  | 22 |  |  | 1 | 2 | 1 | 1 | 2 | 1 |  | 1 | 1 | 2 | 1 | 2 | 1 | 1 | 1 | 1 | 1 | 2 | 1 |
|  | 23 |  |  | 1 | 1 | 2 | 1 | 2 | 1 |  | 1 | 2 | 1 | 1 | 2 | 1 | 1 | 2 | 1 | 1 | 2 | 1 |
|  | 24 |  |  | 1 | 2 | 1 | 1 | 2 | 1 |  | 1 | 1 | 2 | 1 | 2 | 1 | 1 | 2 | 1 | 1 | 2 | 1 |
|  | 25 |  |  | 1 | 1 | 2 | 1 | 2 | 1 |  | 1 | 2 | 1 | 1 | 2 | 1 | 1 | 1 | 1 | 1 | 2 | 1 |
|  | 26 |  |  | 1 | 2 | 1 | 1 | 2 | 1 |  | 1 | 1 | 2 | 1 | 2 | 1 | 1 | 2 | 1 | 1 | 2 | 1 |
|  | 27 |  |  | 1 | 1 | 2 | 1 | 2 | 1 |  | 1 | 2 | 1 | 1 | 2 | 1 | 1 | 2 | 1 | 1 | 2 | 1 |
|  | 28 |  |  | 1 | 2 | 1 | 1 | 2 | 1 |  | 1 | 1 | 2 | 1 | 2 | 1 | 1 | 1 | 1 | 1 | 2 | 1 |
|  | 29 |  |  | 1 | 1 | 2 | 1 | 2 | 1 |  | 1 | 2 | 1 | 1 | 2 | 1 | 1 | 2 | 1 | 1 | 2 | 1 |
|  | 30 |  |  | 1 | 2 | 1 | 1 | 2 | 1 |  | 1 | 2 | 1 | 1 | 2 | 1 | 1 | 2 | 1 | 1 | 2 | 1 |
|  | 31 |  |  | 1 | 1 | 2 | 1 | 2 | 1 |  | 1 | 1 | 2 | 1 | 2 | 1 | 1 | 1 | 1 | 1 | 2 | 1 |
|  | 32 |  |  | 1 | 2 | 1 | 1 | 2 | 1 |  | 1 | 2 | 1 | 1 | 2 | 1 | 1 | 2 | 1 | 1 | 2 | 1 |
|  | 33 |  |  | 1 | 1 | 2 | 1 | 2 | 1 |  | 1 | 1 | 2 | 1 | 2 | 1 | 1 | 2 | 1 | 1 | 2 | 1 |
|  | 34 |  |  | 1 | 2 | 1 | 1 | 2 | 1 |  | 1 | 2 | 1 | 1 | 2 | 1 | 1 | 1 | 1 | 1 | 2 | 1 |
|  | 35 |  |  | 1 | 2 | 1 | 1 | 2 | 1 |  | 1 | 1 | 2 | 1 | 2 | 1 | 1 | 2 | 1 | 1 | 2 | 1 |
|  | 36 |  |  | 1 | 2 | 1 | 1 | 2 | 1 |  | 1 | 2 | 1 | 1 | 2 | 1 | 1 | 2 | 1 | 1 | 2 | 1 |
|  | 37 |  |  | 1 | 2 | 1 | 1 | 2 | 1 |  | 1 | 2 | 1 | 1 | 2 | 1 | 1 | 1 | 1 | 1 | 2 | 1 |
|  | 38 |  |  | 1 | 1 | 2 | 1 | 2 | 1 |  | 1 | 2 | 1 | 1 | 2 | 1 | 1 | 2 | 1 | 1 | 2 | 1 |
|  | 39 |  |  | 1 | 1 | 2 | 1 | 2 | 1 |  | 1 | 2 | 1 | 1 | 2 | 1 | 1 | 2 | 1 | 1 | 2 | 1 |
|  | 40 |  |  | 1 | 1 | 2 | 1 | 2 | 1 |  | 1 | 1 | 2 | 1 | 2 | 1 | 1 | 1 | 1 | 1 | 2 | 1 |
|  | 41 |  |  | 1 | 1 | 2 | 1 | 2 | 1 |  | 1 | 1 | 2 | 1 | 2 | 1 | 1 | 2 | 1 | 1 | 2 | 1 |
|  | 42 |  |  | 1 | 2 | 1 | 1 | 2 | 1 |  | 1 | 2 | 1 | 1 | 2 | 1 | 1 | 2 | 1 | 1 | 2 | 1 |
|  | 43 |  |  | 1 | 1 | 2 | 1 | 2 | 1 |  | 1 | 1 | 2 | 1 | 2 | 1 | 1 | 1 | 1 | 1 | 2 | 1 |
|  | 44 |  |  | 1 | 2 | 1 | 1 | 2 | 1 |  | 1 | 2 | 1 | 1 | 2 | 1 | 1 | 2 | 1 | 1 | 2 | 1 |
|  | 45 |  |  | 1 | 1 | 2 | 1 | 2 | 1 |  | 1 | 1 | 2 | 1 | 2 | 1 | 1 | 1 | 1 | 1 | 2 | 1 |
|  | 46 |  |  | 1 | 2 | 1 | 1 | 2 | 1 |  | 1 | 2 | 1 | 1 | 2 | 1 | 1 | 2 | 1 | 1 | 2 | 1 |
|  | 47 |  |  | 1 | 1 | 2 | 1 | 2 | 1 |  | 1 | 1 | 2 | 1 | 2 | 1 | 1 | 2 | 1 | 1 | 2 | 1 |
|  | 48 |  |  | 1 | 2 | 1 | 1 | 2 | 1 |  | 1 | 2 | 1 | 1 | 2 | 1 | 1 | 1 | 1 | 1 | 2 | 1 |
|  | 49 |  |  | 2 | 2 | 1 | 2 | 3 | 2 |  | 2 | 2 | 1 | 2 | 3 | 2 | 2 | 3 | 2 | 1 | 2 | 2 |
|  | 50 |  |  | 1 | 2 | 1 | 1 | 2 | 1 |  | 2 | 1 | 2 | 2 | 3 | 2 | 2 | 1 | 3 | 2 | 2 | 2 |
|  | 1 | The results  of the tests  are not accurate | Misdiagnosis of the  patient's condition | 2 | 2 | 1 | 2 | 3 | 2 | MeanResponse time for visit in Hour | 1 | 1 | 2 | 1 | 2 | 1 | 1 | 2 | 1 | 1 | 2 | 1 |
|  | 2 |  |  | 4 | 3 | 4 | 5 | 4 | 4 |  | 1 | 2 | 1 | 1 | 2 | 1 | 3 | 2 | 3 | 4 | 3 | 3 |
|  | 3 |  |  | 3 | 2 | 3 | 4 | 3 | 3 |  | 1 | 2 | 1 | 1 | 2 | 1 | 2 | 2 | 3 | 4 | 3 | 2 |
|  | 4 |  |  | 5 | 4 | 5 | 4 | 5 | 5 |  | 1 | 1 | 2 | 1 | 2 | 1 | 3 | 2 | 3 | 4 | 3 | 3 |
|  | 5 |  |  | 1 | 1 | 2 | 1 | 2 | 1 |  | 1 | 2 | 1 | 1 | 2 | 1 | 1 | 2 | 1 | 1 | 2 | 1 |
|  | 6 |  |  | 1 | 2 | 1 | 1 | 2 | 1 |  | 1 | 2 | 1 | 1 | 2 | 1 | 1 | 2 | 1 | 1 | 2 | 1 |
|  | 7 |  |  | 3 | 2 | 3 | 4 | 3 | 3 |  | 3 | 2 | 3 | 4 | 3 | 3 | 1 | 1 | 1 | 1 | 2 | 1 |
|  | 8 |  |  | 5 | 4 | 5 | 4 | 5 | 5 |  | 5 | 4 | 5 | 4 | 5 | 5 | 2 | 1 | 3 | 2 | 2 | 2 |
|  | 9 |  |  | 2 | 2 | 1 | 2 | 3 | 2 |  | 3 | 2 | 3 | 4 | 3 | 3 | 2 | 3 | 2 | 1 | 2 | 2 |
|  | 10 |  |  | 4 | 3 | 4 | 5 | 4 | 4 |  | 4 | 3 | 4 | 5 | 4 | 4 | 2 | 2 | 1 | 2 | 3 | 2 |
|  | 11 |  |  | 4 | 3 | 4 | 4 | 5 | 4 |  | 4 | 3 | 4 | 4 | 5 | 4 | 1 | 2 | 1 | 1 | 2 | 1 |
|  | 12 |  |  | 4 | 4 | 3 | 5 | 4 | 4 |  | 4 | 4 | 3 | 5 | 4 | 4 | 3 | 2 | 3 | 4 | 3 | 3 |
|  | 13 |  |  | 5 | 4 | 5 | 4 | 5 | 5 |  | 5 | 4 | 5 | 4 | 5 | 5 | 3 | 2 | 3 | 4 | 3 | 3 |
|  | 14 |  |  | 4 | 3 | 4 | 5 | 4 | 4 |  | 3 | 2 | 3 | 4 | 3 | 3 | 3 | 2 | 3 | 4 | 3 | 3 |
|  | 15 |  |  | 2 | 2 | 1 | 2 | 3 | 2 |  | 2 | 2 | 1 | 2 | 3 | 2 | 1 | 2 | 1 | 1 | 2 | 1 |
|  | 16 |  |  | 5 | 4 | 5 | 4 | 5 | 5 |  | 4 | 3 | 4 | 5 | 4 | 4 | 2 | 1 | 3 | 2 | 2 | 2 |
|  | 17 |  |  | 4 | 3 | 4 | 5 | 4 | 4 |  | 5 | 4 | 5 | 4 | 5 | 5 | 2 | 1 | 3 | 2 | 2 | 2 |
|  | 18 |  |  | 3 | 2 | 3 | 4 | 3 | 3 |  | 4 | 3 | 4 | 5 | 4 | 4 | 3 | 3 | 2 | 1 | 2 | 3 |
|  | 19 |  |  | 3 | 2 | 3 | 3 | 3 | 3 |  | 3 | 2 | 3 | 4 | 3 | 3 | 2 | 2 | 1 | 2 | 3 | 2 |
|  | 20 |  |  | 4 | 3 | 4 | 5 | 4 | 4 |  | 4 | 3 | 4 | 5 | 4 | 4 | 3 | 2 | 3 | 4 | 3 | 3 |
|  | 21 |  |  | 4 | 3 | 4 | 4 | 5 | 4 |  | 3 | 2 | 3 | 4 | 3 | 3 | 2 | 1 | 3 | 2 | 2 | 2 |
|  | 22 |  |  | 4 | 4 | 3 | 5 | 4 | 4 |  | 5 | 4 | 5 | 4 | 5 | 5 | 2 | 3 | 2 | 1 | 2 | 2 |
|  | 23 |  |  | 3 | 2 | 3 | 4 | 3 | 3 |  | 4 | 3 | 4 | 5 | 4 | 4 | 2 | 2 | 1 | 2 | 3 | 2 |
|  | 24 |  |  | 5 | 4 | 5 | 4 | 5 | 5 |  | 5 | 4 | 5 | 4 | 5 | 5 | 1 | 2 | 1 | 1 | 2 | 1 |
|  | 25 |  |  | 4 | 3 | 4 | 4 | 5 | 4 |  | 4 | 3 | 4 | 4 | 5 | 4 | 1 | 1 | 1 | 1 | 2 | 1 |
|  | 26 |  |  | 4 | 4 | 3 | 5 | 4 | 4 |  | 4 | 4 | 3 | 5 | 4 | 4 | 2 | 1 | 3 | 2 | 2 | 2 |
|  | 27 |  |  | 3 | 2 | 3 | 4 | 3 | 3 |  | 3 | 2 | 3 | 4 | 3 | 3 | 2 | 3 | 2 | 1 | 2 | 2 |
|  | 28 |  |  | 2 | 2 | 1 | 2 | 3 | 2 |  | 2 | 2 | 1 | 2 | 3 | 2 | 2 | 2 | 1 | 2 | 3 | 2 |
|  | 29 |  |  | 4 | 3 | 4 | 5 | 4 | 4 |  | 4 | 3 | 4 | 5 | 4 | 4 | 3 | 2 | 3 | 4 | 3 | 3 |
|  | 30 |  |  | 2 | 2 | 1 | 2 | 3 | 2 |  | 1 | 1 | 1 | 1 | 2 | 1 | 1 | 2 | 1 | 1 | 2 | 1 |
|  | 31 |  |  | 2 | 1 | 2 | 2 | 3 | 2 |  | 2 | 2 | 1 | 2 | 3 | 2 | 2 | 3 | 2 | 1 | 2 | 2 |
|  | 32 |  |  | 4 | 3 | 4 | 5 | 4 | 4 |  | 4 | 3 | 4 | 5 | 4 | 4 | 3 | 2 | 3 | 4 | 3 | 3 |
|  | 33 |  |  | 1 | 1 | 1 | 1 | 2 | 1 |  | 1 | 1 | 1 | 1 | 2 | 1 | 2 | 3 | 2 | 1 | 2 | 2 |
|  | 34 |  |  | 5 | 4 | 5 | 4 | 5 | 5 |  | 4 | 3 | 4 | 4 | 5 | 4 | 4 | 4 | 3 | 5 | 4 | 4 |
|  | 35 |  |  | 3 | 2 | 3 | 4 | 3 | 3 |  | 4 | 4 | 3 | 5 | 4 | 4 | 2 | 1 | 3 | 2 | 2 | 2 |
|  | 36 |  |  | 5 | 4 | 5 | 4 | 5 | 5 |  | 4 | 4 | 3 | 5 | 4 | 4 | 2 | 3 | 2 | 1 | 2 | 2 |
|  | 37 |  |  | 4 | 3 | 4 | 5 | 4 | 4 |  | 4 | 3 | 4 | 4 | 5 | 4 | 3 | 2 | 3 | 4 | 3 | 3 |
|  | 38 |  |  | 4 | 3 | 4 | 4 | 5 | 4 |  | 4 | 4 | 3 | 5 | 4 | 4 | 2 | 1 | 3 | 2 | 2 | 2 |
|  | 39 |  |  | 4 | 4 | 3 | 5 | 4 | 4 |  | 4 | 3 | 4 | 4 | 5 | 4 | 2 | 3 | 2 | 1 | 2 | 2 |
|  | 40 |  |  | 3 | 2 | 3 | 4 | 3 | 3 |  | 4 | 4 | 3 | 5 | 4 | 4 | 2 | 2 | 1 | 2 | 3 | 2 |
|  | 41 |  |  | 5 | 4 | 5 | 4 | 5 | 5 |  | 5 | 4 | 5 | 4 | 5 | 5 | 2 | 1 | 3 | 2 | 2 | 2 |
|  | 42 |  |  | 3 | 2 | 3 | 4 | 3 | 3 |  | 4 | 3 | 4 | 4 | 5 | 4 | 2 | 3 | 2 | 1 | 2 | 2 |
|  | 43 |  |  | 4 | 3 | 4 | 5 | 4 | 4 |  | 4 | 4 | 3 | 5 | 4 | 4 | 2 | 2 | 1 | 2 | 3 | 2 |
|  | 44 |  |  | 4 | 3 | 4 | 4 | 5 | 4 |  | 5 | 4 | 5 | 4 | 5 | 5 | 2 | 1 | 3 | 2 | 2 | 2 |
|  | 45 |  |  | 4 | 4 | 3 | 5 | 4 | 4 |  | 4 | 3 | 4 | 5 | 4 | 4 | 2 | 3 | 2 | 1 | 2 | 2 |
|  | 46 |  |  | 5 | 4 | 5 | 4 | 5 | 5 |  | 5 | 4 | 5 | 4 | 5 | 5 | 2 | 2 | 1 | 2 | 3 | 2 |
|  | 47 |  |  | 5 | 4 | 5 | 5 | 4 | 5 |  | 5 | 4 | 5 | 5 | 4 | 5 | 1 | 2 | 1 | 1 | 2 | 1 |
|  | 48 |  |  | 4 | 3 | 4 | 5 | 4 | 4 |  | 5 | 4 | 5 | 4 | 5 | 5 | 2 | 1 | 3 | 2 | 2 | 2 |
|  | 49 |  |  | 4 | 3 | 4 | 4 | 5 | 4 |  | 4 | 3 | 4 | 5 | 4 | 4 | 2 | 3 | 2 | 1 | 2 | 2 |
|  | 50 |  |  | 4 | 4 | 3 | 5 | 4 | 4 |  | 5 | 4 | 5 | 4 | 5 | 5 | 1 | 2 | 1 | 1 | 2 | 1 |
